# Supplementary material for: Universal and divergent P-stereogenic building with camphor-derived 2,3-diols
Source: Commun Chem. 2023 Jun 27;6:133. doi: 10.1038/s42004-023-00935-0 (PMC10300088; doi:10.1038/s42004-023-00935-0)
Supplement: Supplementary file 2 — Supplementary Information [file 42004_2023_935_MOESM2_ESM.pdf]

Supplementary Information

Universal and divergent P-stereogenic building with camphor-derived 2,3-diols

Yulong Zhang, Peichao Zhao, Shengnan Sun, Qian Wu, Enxue Shi*, and Junhua Xiao*

The corresponding authors: State Key Laboratory of NBC Protection for Civilian, Beijing 102205, China

E-mail: exshi@sina.com; xiao.junhua@pku.edu.cn

Table of Contents

Supplementary Methods 1

General Experimental 1

General procedure for synthesis of CAMDOL **1a-e** 1

General procedure for synthesis of substrates **3a-c** 3

Initial exploration with CAMDOL-phosphonates 4

General procedure for synthesis of intermediates **4b-c** 5

General procedure for lithium-halogen exchange 7

General procedure for synthesis of phosphinous acid-borane **5aa-ak** 7

General procedure for synthesis of phosphinous acid-borane **5ba-br** 23

General procedure for synthesis of phosphinous acid-borane **5ca-cc** 48

General procedure for synthesis of P(III)-ligand PAMP 54

General procedure for synthesis of phosphinites-borane **6** 55

General procedure for synthesis of phosphinates **7** 57

General procedure for synthesis of phosphinothioates **8** 60

General procedure for synthesis of phosphines **9** 63

General procedure for synthesis of phosphine oxides **10** 65

General procedure for synthesis of phosphine sulfides **11** 67

General procedure for synthesis of Secondary Phosphine Oxide **12** 70

X-ray crystal structures 75

X-ray crystal structure of CAMDOL **1e** 75

X-ray crystal structure of camphor epoxide 76

X-ray crystal structure of product **5bo** 77

Supplementary References 78

Supplementary Methods

General Experimental

Tetrahydrofuran (THF), *N,N*-dimethylformamide (DMF), dicloromethane (DCM), acetonitrile (MeCN) and methanol (MeOH) were obtained by passing the previously degassed solvents through an activated alumina column. DABCO was purchased from Chem-Impex.All reagents were purchased at the highest commercial quality and used without further purification unless otherwise stated. Yields refer to chromatographically and spectroscopically (^{1}H NMR) homogeneous material, unless otherwise stated. Reactions weremonitored by thin layer chromatography (TLC), GC/MS, GC/FID, or LC/MS. TLC was performed using 0.25 mm E. Merck silica plates (60F-254), using short-wave UV light as the visualizing agent, andphosphomolybdic acid, *p*-anisaldehyde, or $KMnO_4$ and heat as developing agents. NMR spectra were recorded on Bruker DRX-300 instruments and are calibrated using residual undeuterated solvent ($CHCl_3$, CH_2Cl_2, DMSO, MeOH, acetone at 7.26, 5.32, 2.50, 3.31 and 2.05 ppm for ^{1}H NMR, respectively, and 77.16, 53.84, 39.52, 49.00 and 29.84 ppm for ^{13}C NMR, respectively). The following abbreviations were used to explain multiplicities: s = singlet, d = doublet, t = triplet, q =quartet, m = multiplet, br = broad. Column chromatography was performed using E. Merck silica gel (60, particle size 0.043–0.063 mm), and preparative TLC (pTLC) was performed on Merck silica plates (60F-254). High-resolution mass spectra (HRMS) were recorded on an Agilent LC/Xevo G2-XS QTOF mass spectrometer by electrospray ionization time of flight reflectron experiments. Melting points were recorded on a Fisher-Johns 12-144 melting point apparatus and are uncorrected. The enantiomeric ratios were determined with Waters UPC2 SFC equipped with a photodiode array detector or an Agilent Technologies 1220 Infinity II LC HPLC. Optical rotation data was recorded on an Anton Paar 100 Modular Circular Polarimeter.

General procedure for synthesis of CAMDOL 1a-e

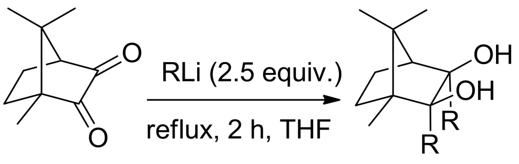

To a flamed-dried 250 mL round-bottom flask was charged (-)-camphorquinone (1 equiv.). The flask was evacuated and back filled with argon, after which anhydrous THF (100 mL, 0.1 M) was introduced via a cannula. Organolithium reagent (2.5 equiv.) was then added dropwise and the resulting solution was allowed to stir at reflux temperature until TLC showed complete consumption of starting material. and the solution was allowed to come back to room temperature. The reaction was quenched with slow addition of saturated aqueous NH_4Cl solution (40 mL) and diluted with water (80 mL) and EtOAc (150mL). The two layers were separated, and the aqueous layer was washed twice with EtOAc (2x 80 mL). The combined organic layers were washed with saturated aqueous brine (50 mL), and dried over anhydrous Na_2SO_4. The mixture was then filtered, concentrated *in vacuo*, and purified by simple filtration to afford the desired product.

(-)-2,3-endo-Dimethyl camphor-2,3-diol (1a):

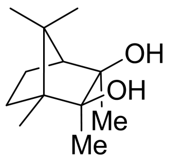

87% yield, white solid, **m.p.** = 108–110 °C, R_f = 0.53 (PE/EA= 10:1), $[\alpha]_D^{25}$ = 127.3° (c = 1.00 in $CHCl_3$); ^1H NMR (300 MHz, $CDCl_3$) δ 2.98 (s, 2H), 1.58 (d, *J* = 22.0 Hz, 2H), 1.43–1.08 (m, 12H), 0.90–0.73 (m, 6H); ^{13}C NMR (75 MHz, $CDCl_3$) δ 82.36, 80.51, 56.82, 53.21, 48.39, 31.02, 24.33, 23.47, 23.32, 22.93, 21.47, 10.83; HRMS (ESI-MS) $[M+H]^+$: found 199.1628; calculated for $C_{12}H_{23}O_2$: 199.1620.

(-)-2,3-endo-Diethyl camphor-2,3-diol (1b):

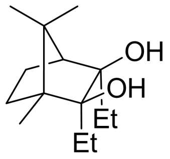

84% yield, white solid, **m.p.** = 99–102 °C, R_f = 0.62 (PE/EA= 10:1), $[\alpha]_D^{25}$ =107.4° (c = 1.00 in $CHCl_3$); ^1H NMR (300 MHz, $CDCl_3$) δ 2.68 (s, 1H), 2.53 (s, 1H), 2.01–1.81 (m, 1H), 1.81–1.12 (m, 12H), 1.03–0.74 (m, 12H); 13C NMR (75 MHz, $CDCl_3$) δ 83.79, 83.44, 53.60, 52.80, 48.30, 29.82, 28.77, 27.14, 23.37, 23.25, 22.68, 11.68, 9.52, 8.97; HRMS (ESI-MS) $[M+H]^+$: found 227.1945; calculated for $C_{14}H_{27}O_2$: 227.1933.

(-)-2,3-endo-Dibutyl camphor-2,3-diol (1c):

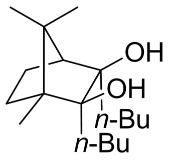

80% yield, white solid, **m.p.** = 115–119 °C, R_f = 0.50 (PE/EA= 10:1), $[\alpha]_D^{25}$ = 84.3° (c = 1.00 in $CHCl_3$); ^1H NMR (300 MHz, $CDCl_3$) δ 2.72 (s, 1H), 2.66 (s, 1H), 1.88–1.75 (m, 1H), 1.70 (d, J = 4.6 Hz, 2H), 1.59–1.28 (m, 10H), 1.26 (s, 6H), 0.89 (q, *J* = 4.6, 2.7 Hz, 8H), 0.81 (s, 3H); ^{13}C NMR (75 MHz, $CDCl_3$) δ 83.59 (d, *J* = 11.4 Hz), 53.49 (d, *J* = 7.5 Hz), 48.43, 36.22, 34.97, 29.99, 27.09 (d, *J* = 23.4 Hz), 24.61–22.44 (m), 14.24 (d, *J* = 1.2 Hz), 11.80; HRMS (ESI-MS) $[M+H]^+$: found 283.2550; calculated for $C_{18}H_{35}O_2$: 283.2559.

(-)-2,3-endo-Dibenzyl camphor-2,3-diol (1d):

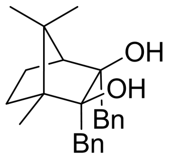

75% yield, white solid, **m.p.** = 104–107°C, R_f = 0.57 (PE/EA= 10:1), $[\alpha]_D^{25}$ = 134.2° (c = 1.00 in $CHCl_3$); ^1H NMR (300 MHz, $CDCl_3$) δ 7.50 (t, *J* = 4.9 Hz, 2H), 7.45–7.20 (m, 9H), 3.40 (dt, *J* = 13.6, 2.5 Hz, 1H), 3.33–3.24 (m, 1H), 3.17 (dt, *J* = 14.0, 2.6 Hz, 1H), 3.10–2.96 (m, 1H), 2.87 (dt, *J* = 13.5, 2.6 Hz, 1H), 2.71–2.58 (m, 1H), 1.85 (p, *J* =10.0 Hz, 3H), 1.57–1.36 (m, 2H), 1.24 (t, *J* =2.4 Hz, 3H), 0.81 (t, *J* = 2.5 Hz, 3H), 0.56–0.42 (m, 3H); ^{13}C NMR (75 MHz, $CDCl_3$) δ 138.66, 138.48, 131.26, 130.63, 128.36, 128.10, 126.58, 126.40, 84.07, 83.80, 77.37, 54.03, 53.10, 48.76, 41.26, 41.21, 30.22, 23.85, 23.50, 23.32, 11.54; HRMS (ESI-MS) $[M+H]^+$: found 351.2251;calculated for $C_{24}H_{31}O_2$: 351.2246.

(-)-2,3-endo-Diphenyl camphor-2,3-diol (1e) (CCDC 2268813):

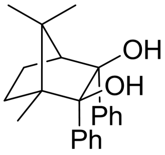

85% yield, white solid, **m.p.** = 114–117°C, R_f = 0.58 (PE/EA= 10:1), $[\alpha]_D^{25}$ = 104.2° (c = 1.00 in $CHCl_3$); ^1H NMR (300 MHz, $CDCl_3$) δ 7.17 (t, *J* = 26.3 Hz, 10H), 4.66 (s, 1H), 2.47 (d, *J* = 5.6 Hz, 2H), 2.08–1.88 (m, 1H), 1.82 (d, *J* = 7.1 Hz, 1H), 1.49 (s, 3H), 1.18 (d, *J* = 4.2 Hz, 2H), 1.05 (s, 3H), 0.83 (s, 3H); ^{13}C NMR (75 MHz, $CDCl_3$) δ 143.91, 142.53, 127.90, 127.41, 125.95, 88.37, 86.85, 54.52, 53.02, 49.92, 30.89, 24.41, 23.96, 23.75, 10.36; HRMS (ESI-MS) [M+H]+: found 323.2006; calculated for $C_{22}H_{27}O_2$: 323.1933.

General procedure for synthesis of substrates 3a-c

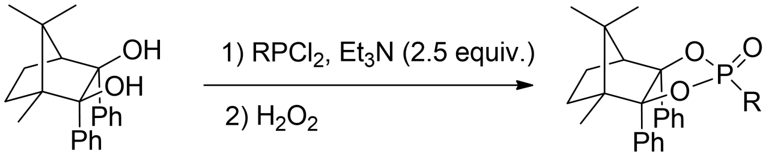

CAMDOL, 1e **3**

A Schlenk tube was dried under vacuum. After cooling, the tube was placed under argon atmosphere. Under an argon atmosphere, to a stirred solution of CAMDOL (**1e**, 1 equiv.) in dry THF was added triethylamine (2.5 equiv.). To the mixture at 0 °C was added phosphine dichloride (1.5 equiv.) and the mixture remained at 0 °C. After adding the phosphine dichloride, the cooling bath was removed and the solution was stirred at r.t. for 2 h. the solution was oxidized with conc. H_2O_2 with ^{31}P NMR analysis of a small aliquot showed complete consumption. To the resulting mixture was added saturated aqueous NH_4Cl solution (20 mL) and EtOAc (40 mL). The layers were separated, and the aqueous layer was washed with EtOAc (2 x 20 mL). The combined organic layers were washed with brine (20 mL), dried over anhydrous Na_2SO_4, filtered and concentrated. The residue was purified by silica gel chromatography to afford the desired product **3b-3c**. Substrates **3a** are generally used as crude intermediates without isolation in the following reactions.

2,3-Diphenyl camphor methylphosphonate 3b:

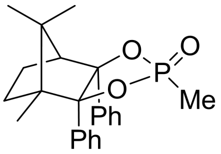

86% yield, white solid, **m.p.** = 138–140 °C, R_f = 0.48 (PE/EA= 1:1), $[\alpha]_D^{25}$ = 124.3° (c = 1.00 in $CHCl_3$); ^{1}H NMR (300 MHz, $CDCl_3$) δ 7.31–7.13 (dq, *J* = 17.8, 6.4, 5.9 Hz, 10H), 3.02 (d, *J* = 5.4 Hz, 1H), 2.14–2.02 (m, 1H), 1.79 (s, 3H), 1.54 (d, *J* = 18.0 Hz, 3H), 1.41–1.24 (m, 2H), 1.16 (s, 3H); ^{13}C NMR (75 MHz, $CDCl_3$) δ 138.60 (d, *J* = 3.0 Hz), 136.3 (d, *J* = 3.8 Hz), 130.07 (d, *J* = 18.0 Hz), 128.04 (d, *J* = 15.8 Hz), 127.54, 126.43, 99.92, 98.00, 55.45 (d, *J* = 3.8 Hz), 51.94 (d, *J* = 3.0 Hz), 48.32, 29.35, 26.29, 24.47, 21.57, 16.07, 14.30, 10.04; ^{31}P NMR (122 MHz, $CDCl_3$) δ 41.44; HRMS (ESI-MS) $[M+H]^+$: found 383.1695;calculated for $C_{23}H_{29}O_3P$:383.1698.

2,3-Diphenyl camphor phenylphosphonate 3c:

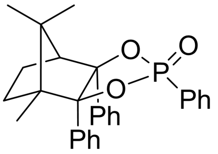

88% yield, white solid, **m.p.** = 112–115 °C, R_f = 0.38 (PE/EA= 1:1), $[\alpha]_D^{25}$ = 104.3° (c = 1.00 in $CHCl_3$); ^{1}H NMR (300 MHz, $CDCl_3$) δ 7.83 (dd, *J* = 13.4, 6.9 Hz, 2H), 7.61–7.36 (m, 6H), 7.25 (s, 7H), 2.96 (d, *J* = 5.3 Hz, 1H), 2.22–1.98 (m, 1H), 1.96–1.73 (m, 1H), 1.53–1.18 (m, 3H), 1.10 (s, 3H), 1.04 (s, 3H), 0.89 (s, 3H); ^{13}C NMR (75 MHz, $CDCl_3$) δ 137.46 (d, *J* = 69.8 Hz), 131.92 (d, *J* = 3.0 Hz), 130.36 (d, *J* = 9.75 Hz), 128.33(d, *J* = 15.0 Hz), 127.93, 127.51, 127.18, 100.40(d, *J* = 0.75 Hz), 98.76 (d, *J* = 2.3 Hz), 55.25 (d, *J* = 4.5 Hz), 51.60 (d, *J* = 5.3 Hz), 48.24, 29.58, 25.64, 24.69, 22.73, 10.01; ^{31}P NMR (122 MHz, $CDCl_3$) δ 31.82; HRMS (ESI-MS) $[M+H]^+$: found 445.1858; calculated for $C_{28}H_{30}O_3P$: 445.1854.

Initial explorations with CAMDOL-phosphonates

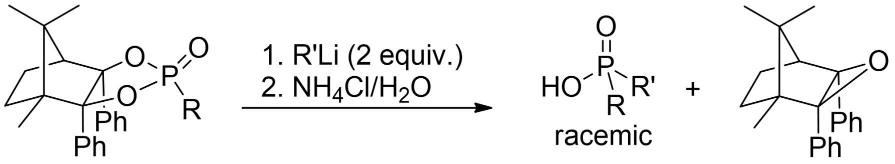

The organolithium reagents (2.0 mmol, 2.0 equiv.) in 5 mL of THF was cooled to 0 °C if not already at such temperature. A solution of the starting material (1.0 mmol, 1.0 equiv.) in 2 mL THF was prepared in a flame-dried flask under argon atmosphere, which was then added dropwise to the flask containing the organolithium reagents. The resulting mixture was stirred for 3 h while being kept at 0 °C. To the resulting mixture was added saturated aqueous NH_4Cl solution (20 mL) and EtOAc (40 mL). The layers were separated, and the aqueous layer was washed with EtOAc (2

x 20 mL). The combined organic layers were washed with brine (20 mL), dried over anhydrous Na_2SO_4, filtered and concentrated. Camphor epoxide, the by-product, would always crystallize out from the concentrated aqueous mixture at the end-point of the reaction.

Data of the camphor epoxide (CCDC 2233676):

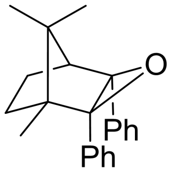

261.4 mg (88% yield), Glassy solid, **m.p.** = 94–98 °C, R_f = 0.53 (PE/EA= 10:1), $[\alpha]_D^{25}$ = 103.4° (c = 1.00 in $CHCl_3$); ^{1}H NMR (300 MHz, $CDCl_3$) δ 7.19 (dt, *J* = 5.1, 3.0 Hz, 3H), 7.14–7.01 (m, 5H), 6.98–6.83 (m, 2H), 2.51 (d, *J* = 4.0 Hz, 1H), 1.92 (ddt, *J* = 14.0, 7.6, 3.8 Hz, 1H), 1.71 (d, *J* = 4.3 Hz, 1H), 1.60 (ddd, *J* = 12.6, 9.0, 3.5 Hz, 1H), 1.39–1.30 (m, 1H), 1.28 (s, 3H), 0.85 (d, *J* = 2.5 Hz, 6H);^{13}C NMR (75 MHz, $CDCl_3$) δ 135.53, 132.80, 130.26, 128.03, 127.71, 127.59, 127.52, 127.09, 74.73, 67.68, 52.09, 48.95, 43.03, 30.68, 25.97, 23.57, 23.16, 11.57; HRMS (ESI-MS) $[M+H]^+$: found 305.1827; calculated for $C_{22}H_{25}O$: 305.1827.

General procedure for synthesis of intermediates 4b-c

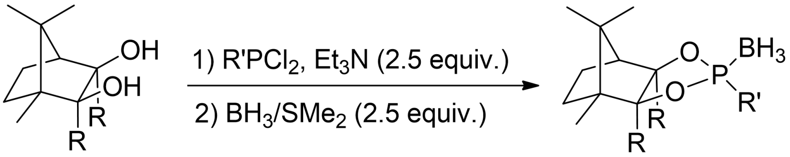

CAMDOL, 1 **4**

A Schlenk tube was dried under vacuum. After cooling under argon atmosphere, to a stirred solution of (-)-CAMDOL (**1**, 1 equiv.) in dry THF was added triethylamine (2.5 equiv.). To the mixture at 0 °C was added phosphine dichloride (1.5 equiv.) and the mixture remained at 0 °C. After ^{31}P NMR analysis of a small aliquot showed complete consumption of starting material, a solution of BH_3/SMe_2 (2.5 equiv.) in THF (100 mL) was added dropwise over 10 min and the solution wasstirred at 0 °C overnight (12 h). To the resulting mixture was added saturated aqueous NH_4Cl solution (20 mL) and EtOAc (40 mL). The layers were separated, and the aqueous layer was washed with EtOAc (2 x 20 mL). The combined organic layers were washed with brine (20 mL), dried over anhydrous Na_2SO_4, filtered and concentrated. The residue was purified by silica gel chromatography to afford the desired product **4be** and **4ce**. Compounds **4ca-4cd** are generally used as crude intermediates without isolation in the following reactions.

Diphenyl CAMDOL-phenylphosphonate borane 4ce:

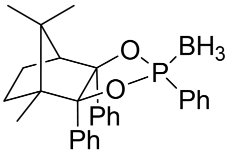

83% yield, white solid, **m.p.** = 108–110 °C, R_f = 0.50 (PE/EA= 10:1), $[\alpha]_D^{25}$ = 100.4° (c = 1.00 in $CHCl_3$); ^1H NMR (300 MHz, $CDCl_3$) δ 7.25–7.03 (m, 15H), 3.12 (d, *J* = 5.0 Hz, 1H), 2.02 (t, *J* = 12.7 Hz, 1H), 1.70 (s, 3H), 1.44 (dt, *J* = 14.3, 7.2 Hz, 1H), 1.26 (td, *J* = 18.9, 16.4, 9.0 Hz, 2H), 1.13 (s, 4H), 1.00 (s, 3H); ^{13}C NMR (75 MHz, $CDCl_3$) δ 136.29 (d, *J* = 30.3 Hz), 134.63 (d, *J* = 54.5 Hz), 131.04, 130.63, 130.12 (d, *J* = 11.0 Hz), 127.78 (d, *J* = 2.6 Hz), 127.65 (d, *J* = 9.9 Hz), 102.37 (d, *J* = 8.6 Hz), 100.20 (d, *J* = 8.7 Hz), 55.86 (d, *J* = 3.8 Hz), 52.46 (d, *J* = 4.4 Hz), 47.80, 29.89, 26.02, 24.01, 22.85 (d, *J* = 3.5 Hz), 9.99; ^{31}P NMR (122 MHz, $CDCl_3$) δ 150.14 (q, *J* = 85.1 Hz); HRMS (ESI-MS) $[M+H]^+$: found 441.2235; calculated for $C_{28}H_{32}BO_2P$: 441.2233.

Diphenyl CAMDOL-phenylphosphonate borane 4be:

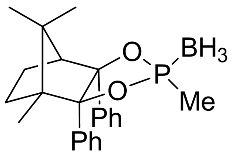

87% yield, white solid, **m.p.** = 132–135°C, R_f = 0.56 (PE/EA= 10:1), $[\alpha]_D^{25}$ = 125.4° (c = 1.00 in $CHCl_3$); ^1H NMR (300 MHz, $CDCl_3$) δ 7.64–6.82 (m, 10H), 3.07 (d, *J* = 4.8 Hz, 1H), 2.78 (q, *J* = 7.2 Hz, 2H), 2.19–1.87 (m, 1H), 1.56 (s, 3H), 1.41 (d, *J* = 7.3 Hz, 4H), 1.19 (t, *J* = 7.3 Hz, 3H), 1.11 (s, 3H), 0.86 (s, 3H); ^{13}C NMR (75 MHz, $CDCl_3$)δ 138.12 , 136.50 , 130.73 , 130.37 , 128.08 , 127.58 , 126.39 , 101.15 (d, *J* = 8.6 Hz), 99.33 (d, *J* = 8.7 Hz), 56.01 (d, *J* = 4.0 Hz), 52.61 (d, *J* = 3.9 Hz), 52.40 , 47.86 , 29.16 , 25.92 , 24.29 , 22.40 (d, *J* = 3.4 Hz), 19.87 (d, *J* = 29.6 Hz), 10.18 , 8.68; ^{31}P NMR (122 MHz, $CDCl_3$) δ 162.28 (q, *J* = 91.8 Hz); HRMS (ESI-MS) $[M+H]^+$: found 379.2077; calculated for $C_{23}H_{29}BO_2P$: 379.2076.

General procedure for lithium-halogen exchange

To a flame dried round-bottom flask under argon atmosphere was charged arylbromide (12 mmol, 1.2 equiv.) and THF (40 mL). The mixture was cooled to –78 °C and *n*BuLi solution (12 mmol, 1.2 equiv.) was added dropwise. The resulting mixture was allowed to stir for 30 min at –78 °C and used directly. The following organolithium reagents were synthesized using the above methods. Isopropyl and tertiarybutyl organolithium reagents (1.3 M) were purchased from Sigma-Aldrich.

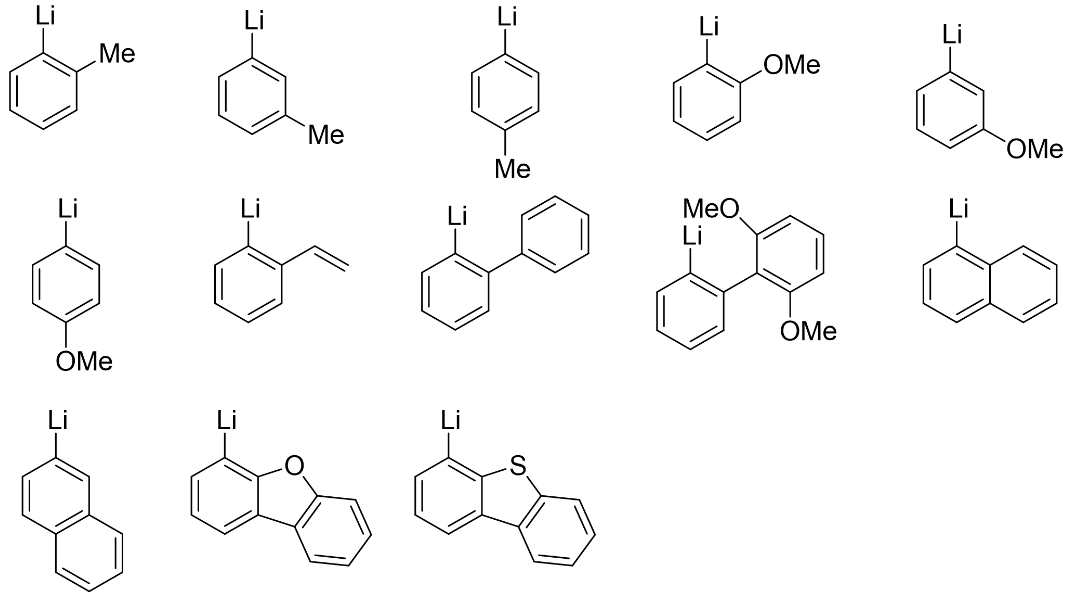

General procedure for synthesis of phosphinous acid-borane 5aa-aj

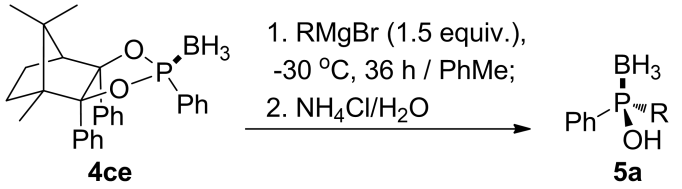

The Grignard reagents (1.5 mmol, 1.5 equiv.) in 5 mL of THF was cooled to –30 °C if not already at such temperature. A solution of the starting material (1.0 mmol, 1.0 equiv.) in 2 mL THF was prepared in a flame-dried flask under argon atmosphere, which was then added dropwise to the flask containing the Grignard reagents. The resulting mixture was stirred for 36 h while being kept at –30 °C. After ^{31}P NMR analysis of a small aliquot showed complete consumption of starting material, to the resulting mixture was added saturated aqueous NH_4Cl solution (20 mL) and EtOAc (40 mL). The layers were separated, and the aqueous layer was washed with EtOAc (2 x 20 mL). The combined organic layers were washed with brine (20 mL), dried over anhydrous Na_2SO_4, filtered and concentrated. The residue was purified by silica gel chromatography to afford the desired product.

Methylphenyphosphinous acid-borane 5aa:

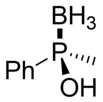

Enantiomeric excess: 97%. 134 mg (86% yield), yellow oil, R_f = 0.53 (PE/EA= 5:1), $[\alpha]_D^{25}$ = 24.3° (c = 1.00 in $CHCl_3$); ^1H NMR (300 MHz, $CDCl_3$) δ 7.87–7.71 (m, 2H), 7.51–7.48 (m, 3H), 4.63 (bs, 1H), 1.73 (d, *J* = 9.4 Hz, 3H), 1.17–0.18 (m, 3H); ^{13}C NMR (75 MHz, $CDCl_3$) δ 131.94 (d, *J* = 1.5 Hz), 129.95 (d, *J* = 11.5 Hz), 128.73 (d, *J* = 10.5 Hz), 17.55 (d, *J* = 44.3 Hz); ^{31}P NMR (122 MHz, $CDCl_3$) δ 101.81 (q, *J* = 204.9 Hz); HRMS (ESI-MS) $[M-H]^+$: found 153.0641; calculated for $C_7H_{11}BOP$: 153.0641.

Chiral HPLC: Chiralpak AS-RH column, Water/Acetonitrile= 75/25, flow rate = 1.0 mL/min, λ = 254 nm.

***RAC*-5aa**

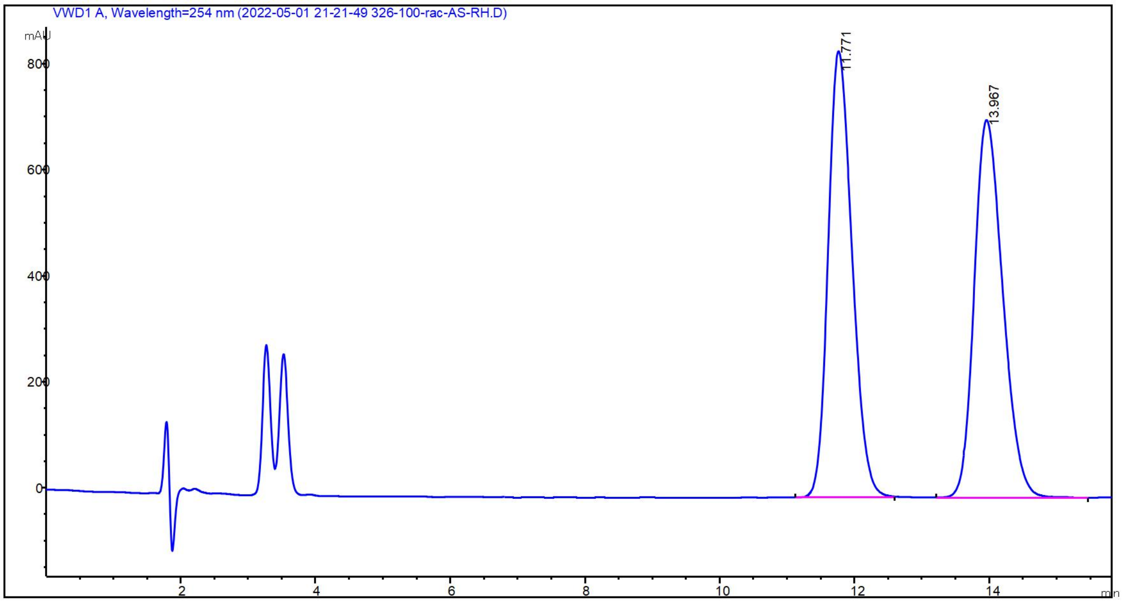

(R_p)-5aa

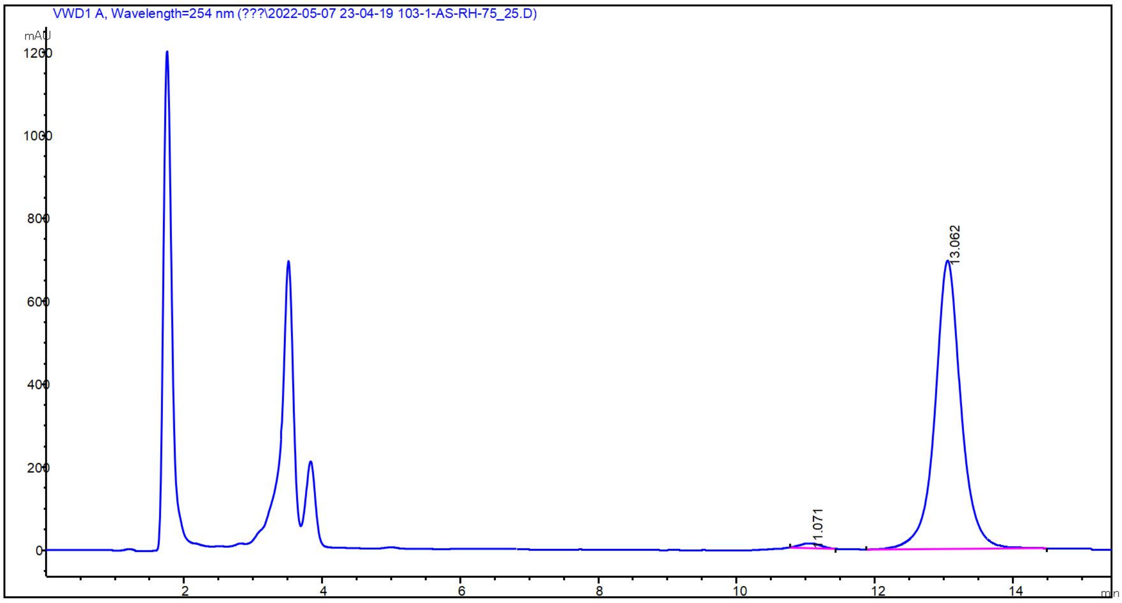

(S_p)-5aa

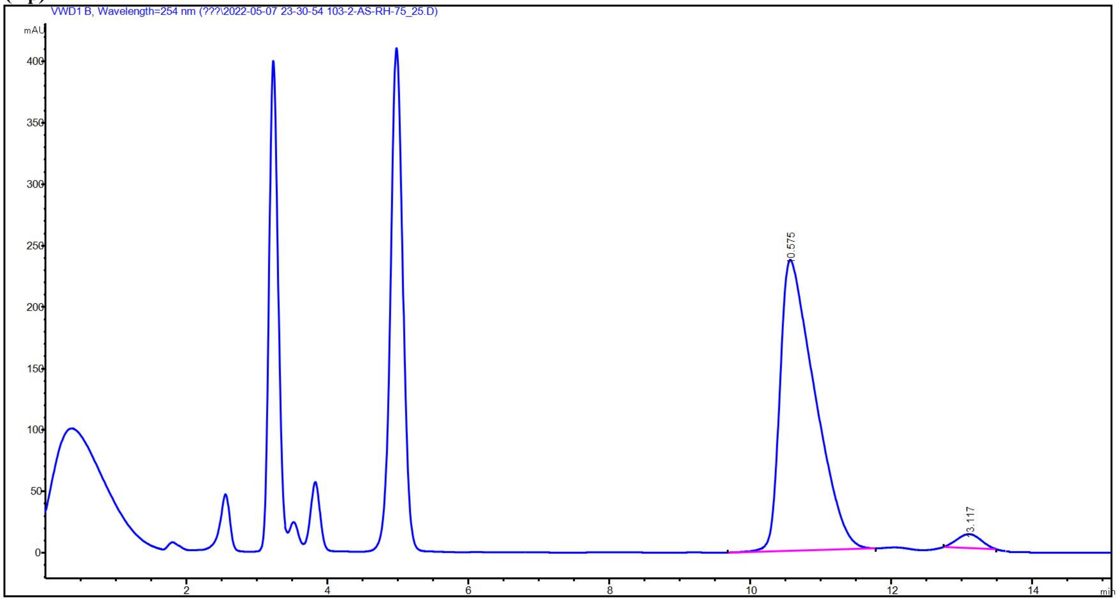

***RAC*-5aa**

Index	t_R/ (min)	Height / (mv)	Area / (mv.sec)	Area / (%)
1	11.77	841.3	20816.8	49.8
2	13.96	172.2	21013.6	50.2
Total				100.0

(R_p)-5aa

Index	t_R/ (min)	Height / (mv)	Area / (mv.sec)	Area / (%)
1	11.07	11.9	245.40	1.3
2	13.06	694.7	18216.5	98.7
Total				100.0

(S_p)-5aa

Index	t_R / (min)	Height / (mv)	Area /	Area / (%)
1	10.57	237	8110.4	96.9
2	13.11	11.4	270.1	3.1
Total			3997.14	100.0

Ethylphenyphosphinous acid-borane 5ab:

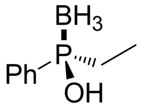

Enantiomeric excess: 94%. 141.4 mg (84% yield), yellow oil, R_f = 0.39 (PE/EA= 5:1), $[\alpha]_D^{25}$ = 5.4° (c = 1.00 in $CHCl_3$); ^1H NMR (300 MHz, $CDCl_3$) δ 7.93–7.62 (m, 2H), 7.49 (d, J = 7.8 Hz,

3H), 4.50 (bs, 1H), 1.96 (q, J = 14.3, 7.3 Hz, 2H), 1.08 (t, J = 15.9, 7.4 Hz, 3H), 0.99–0.18 (m, 3H); ^{13}C NMR (75 MHz, $CDCl_3$) δ 131.78 (d, J = 2.3 Hz), 130.30 (d, J = 11.3 Hz), 128.63 (d, J = 10.5 Hz), 24.43 (d, J = 44.3 Hz), 6.07 (d, J = 2.3 Hz); δ 131.94 (d, J = 1.5 Hz), 129.95 (d, J = 11.5 Hz), 128.73 (d, J = 10.5 Hz), 17.55 (d, J = 44.3 Hz); ^{31}P NMR (122 MHz, $CDCl_3$) δ 106.51 (q, J = 201.3 Hz); HRMS (ESI-MS) $[M-H]^+$: found 167.0797; calculated for $C_8H_{13}BOP$: 167.0797.

Chiral HPLC: Chiralpak OJ-RH column, Water/Acetonitrile= 60/40, flow rate = 1.0 mL/min, λ = 254 nm.

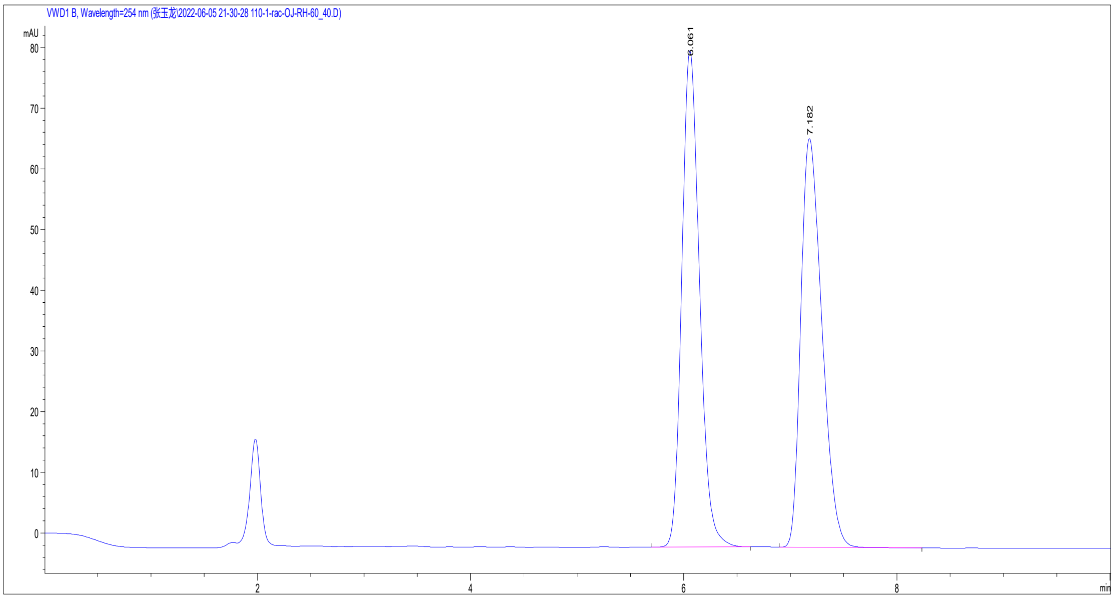

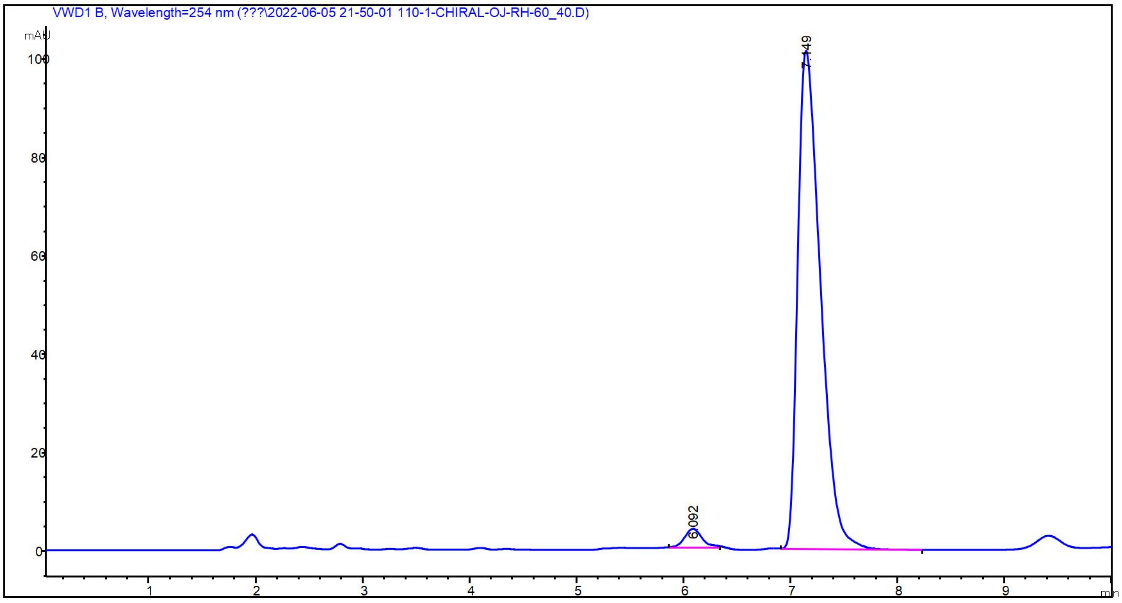

Index	t_R/ (min)	Height / (mv)	Area / (mv.sec)	Area / (%)
1	6.06	81.8	943.9	50.4
2	7.18	67.3	929.2	49.6
Total				100.0

Index	t_R/ (min)	Height / (mv)	Area / (mv.sec)	Area / (%)
1	6.09	3.8	42.4	2.7
2	7.14	101.3	1492.1	97.2
Total				100.0

Isopropylphenyphosphinous acid-borane 5ac:

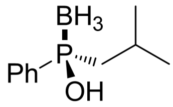

Enantiomeric excess: 90%. 180.0 mg (92% yield), yellow oil, R_f = 0.60 (PE/EA= 5:1), $[\alpha]_D^{25}$ = 13.4° (c = 1.00 in $CHCl_3$); ^1H NMR (300 MHz, $CDCl_3$)δ 7.85–7.67 (m, 2H), 7.48 (d, J = 6.8 Hz, 3H), 2.15–1.97 (m, 1H), 1.95–1.80 (m, 2H), 0.98 (dd, J = 13.4, 6.3 Hz, 6H). ^{13}C NMR (75 MHz, $CDCl_3$) δ 131.62 (d, J = 2 Hz), 130.10 (d, J = 11.5 Hz), 128.62 (d, J = 10.5 Hz), 40.55 (d, J = 40.5 Hz), 24.72 (d, J = 9.0 Hz), 24.28 (d, J = 6.8 Hz), 23.89; ^{31}P NMR (122 MHz, $CDCl_3$) δ 104.54 (q, J = 193.98 Hz); HRMS (ESI-MS) $[M-H]^+$: found 195.1190; calculated for $C_{10}H_{17}BOP$: 195.1188.

Chiral HPLC: Chiralpak OJ-RH column, Water/Acetonitrile= 60/40, flow rate = 1.0 mL/min, λ = 254 nm.

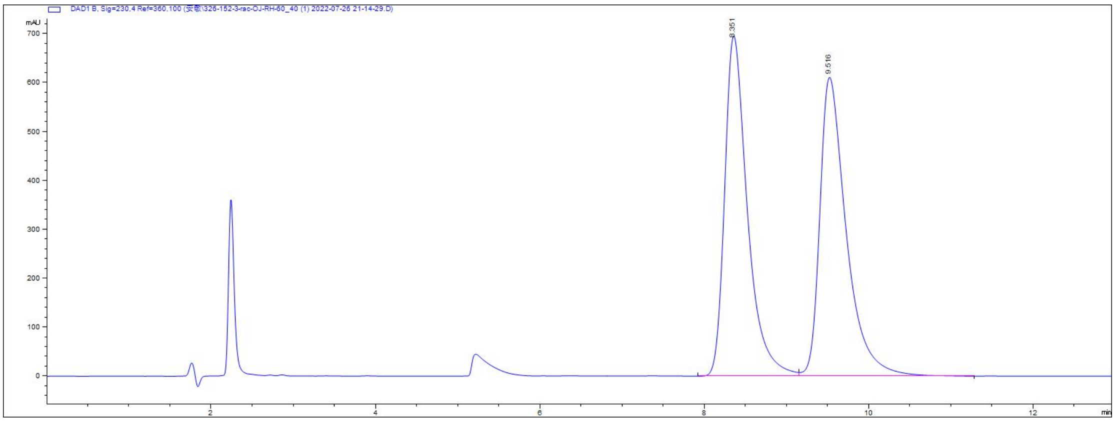

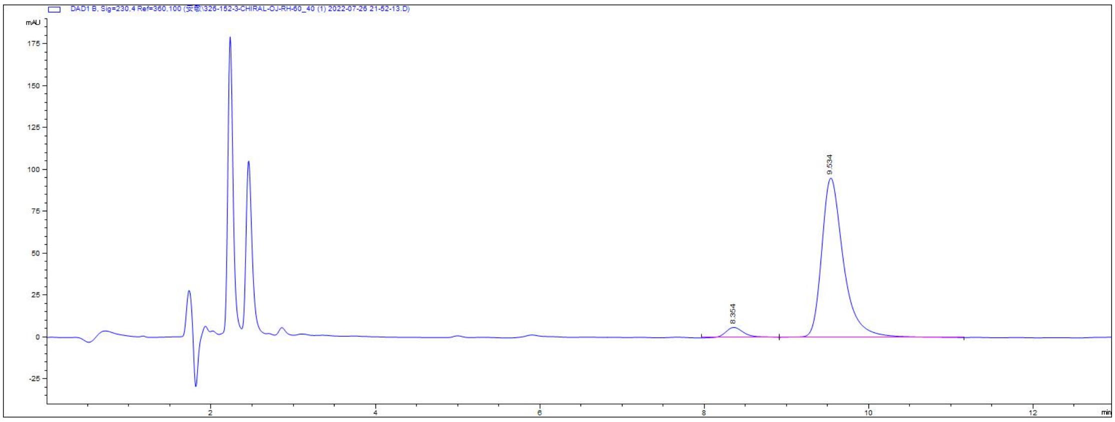

Index	t_R/ (min)	Height / (mv)	Area / (mv.sec)	Area / (%)
1	8.35	695.2	13376.7	49.5
2	9.51	610.4	13629.3	50.5

Total				100.0

Index	t_R/ (min)	Height / (mv)	Area / (mv.sec)	Area / (%)
1	8.35	6.1	96.0	5.1
2	9.53	95.2	175.7	94.9
Total				100.0

Allylphenyphosphinous acid-borane 5ad:

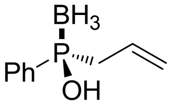

Enantiomeric excess: 91%. 162.0 mg (90% yield), yellow oil, R_f = 0.48 (PE/EA= 5:1), $[\alpha]_D^{25}$ = 28.3° (c = 1.00 in $CHCl_3$); ^1H NMR (300 MHz, $CDCl_3$) δ 7.91–7.64 (m, 2H), 7.54–7.44 (m, 3H), 5.86–5.51 (m, 1H), 5.10 (dd, J = 24.9, 13.5 Hz, 2H), 4.65 (bs, 1H), 2.97–2.56 (m, 2H), 1.35–0.51 (m, 3H), ^{13}C NMR (75 MHz, $CDCl_3$) δ 131.90 (d, J = 60 Hz), 131.89 (d, J = 3 Hz),130.47(d, J = 11.3 Hz),128.58 (d, J = 11.3 Hz), 127.7 (d, J = 6.8 Hz),120.84 (d, J = 11.25 Hz), 37.35 (d, J = 39.8 Hz); ^{31}P NMR (122 MHz, $CDCl_3$) δ 100.91 (q, J = 189.1 Hz); HRMS (ESI-MS) $[M-H]^+$: found 179.0878; calculated for $C_9H_{13}BOP$: 179.0875.

Chiral HPLC: Chiralpak OJ-RH column, Water/Acetonitrile= 60/40, flow rate = 1.0 mL/min, λ = 254 nm.

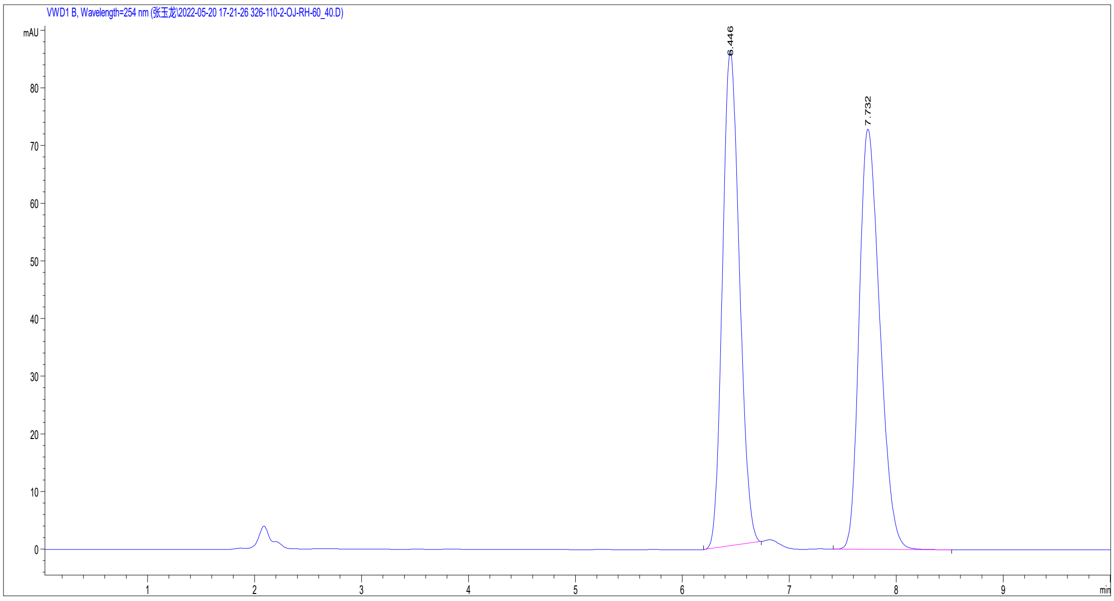

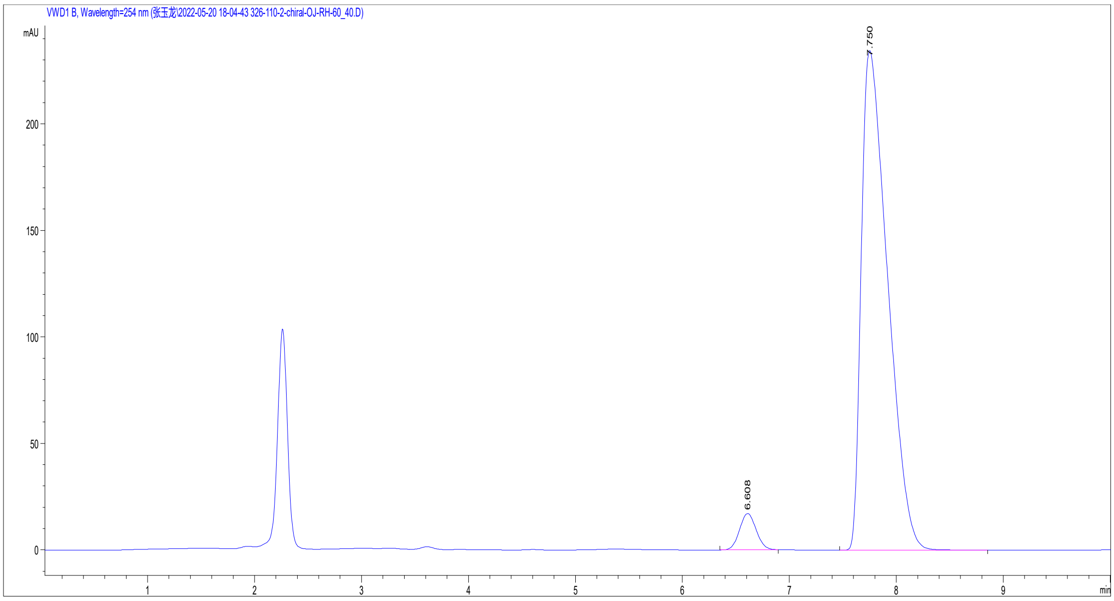

Index	t_R/ (min)	Height / (mv)	Area / (mv.sec)	Area / (%)
1	6.44	85.9	947.8	49.2
2	7.73	72.9	975.8	50.8
Total				100.0

Index	t_R/ (min)	Height / (mv)	Area / (mv.sec)	Area / (%)
1	6.60	17	183	4.4
2	7.77	234.6	3995.5	95.6
Total				100.0

Cyclopropylphenyphosphinous acid-borane 5ae:

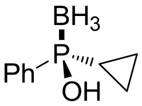

Enantiomeric excess: 93%. 144.0 mg (80% yield), colorless oil, R_f = 0.67 (PE/EA= 5:1), $[\alpha]_D^{25}$ = 38.4° (c = 1.00 in $CHCl_3$); ^{1}H NMR (300 MHz, $CDCl_3$) δ 7.90–7.62 (m, 2H), 7.51–7.47 (m, 3H), 1.20–1.04 (m, 2H), 1.02–0.82 (m, 4H), 0.80–0.24 (m, 2H); ^{13}C NMR (75 MHz, $CDCl_3$) δ 132.33 (d, J = 118.5 Hz), 131.56 (d, J = 2.25 Hz), 130.24 (d, J = 11.2 Hz), 128.54 (d, J = 10.5 Hz), 9.44 (d, J = 66.7 Hz), 8.99, 3.90 (d, J = 29.3 Hz); ^{31}P NMR (122 MHz, $CDCl_3$) δ 106.03 (q, J = 209.8 Hz); HRMS (ESI-MS) $[M-H]^+$: found 179.0873; calculated for $C_9H_{13}BOP$: 179.0875.

Chiral HPLC: Chiralpak OJ-RH column, Water/Acetonitrile= 60/40, flow rate = 1.0 mL/min, λ = 254 nm.

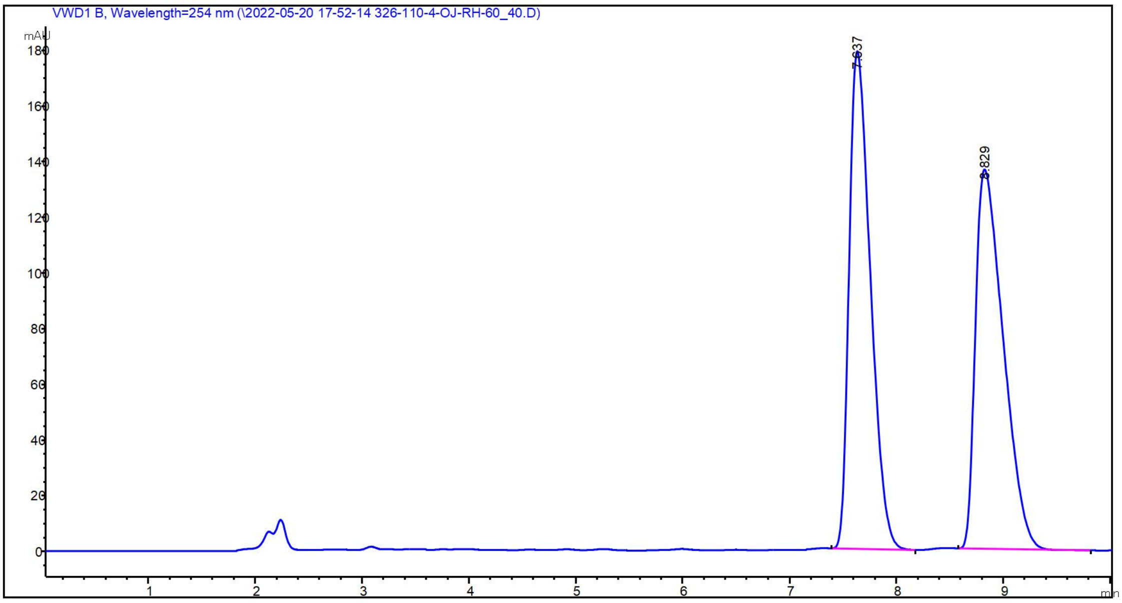

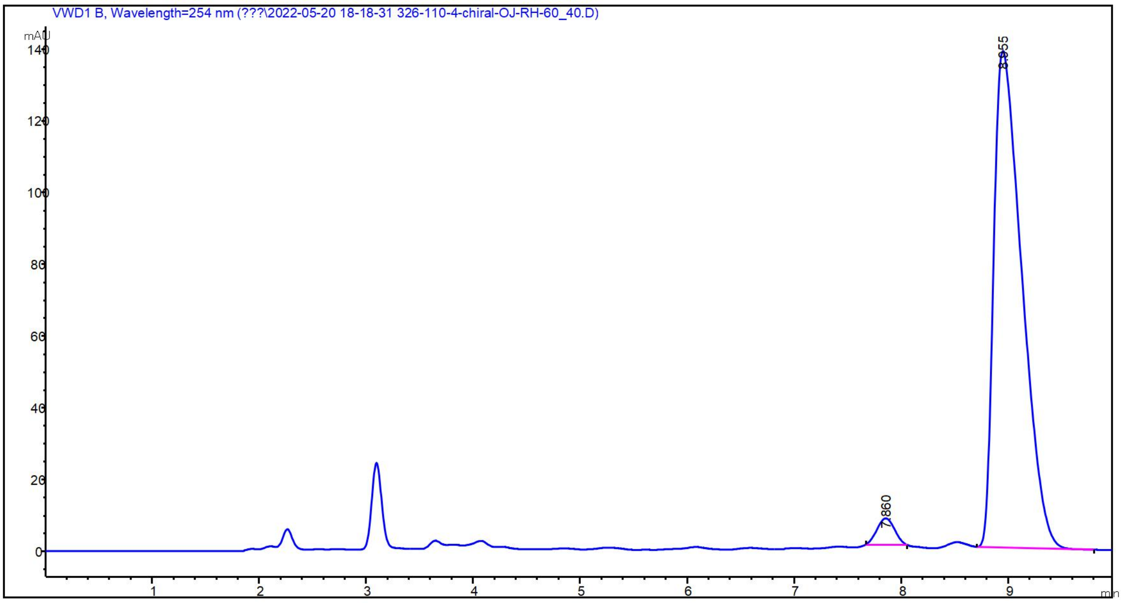

Index	t_R/ (min)	Height / (mv)	Area / (mv.sec)	Area / (%)
1	7.63	178.9	2427.2	50.09
2	8.82	136.4	2465.2	49.91
Total				100.0

Index	t_R/ (min)	Height / (mv)	Area / (mv.sec)	Area / (%)
1	7.86	7.3	80.8	3.2
2	8.95	138.4	2523.8	96.8
Total				100.0

Ethenylphenyphosphinous acid-borane 5af:

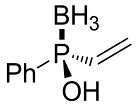

Enantiomeric excess: 95%. 154.4 mg (92% yield), colorless oil, R_f = 0.50 (PE/EA= 5:1), $[\alpha]_D^{25}$ = 9.3° (c = 1.00 in $CHCl_3$); ^1H NMR (300 MHz, $CDCl_3$) δ 7.91–7.67 (m, 2H), 7.55–7.26 (m,3H), 6.50–6.33 (m, 1H), 6.29–6.01 (m, 2H), 4.51 (bs, 1H), 1.17–0.25 (m, 3H); ^{13}C NMR (75 MHz, $CDCl_3$) δ 133.00 (d, J = 6.0 Hz), 132.34 (d, J = 11.2 Hz), 131.94 (d, J = 2.3 Hz), 131.49 (d, J = 6.0 Hz), 130.65 (d, J = 12 Hz), 128.70 (d, J = 6.0 Hz); ^{31}P NMR (122 MHz, $CDCl_3$) δ 92.88 (q, J = 201.3 Hz); HRMS (ESI-MS) $[M-H]^+$: found 165.0723; calculated for $C_8H_{11}BOP$: 165.0719.

Chiral HPLC: Chiralpak AS-RH column, Water/Acetonitrile= 65/35, flow rate = 1.0 mL/min, λ = 254 nm.

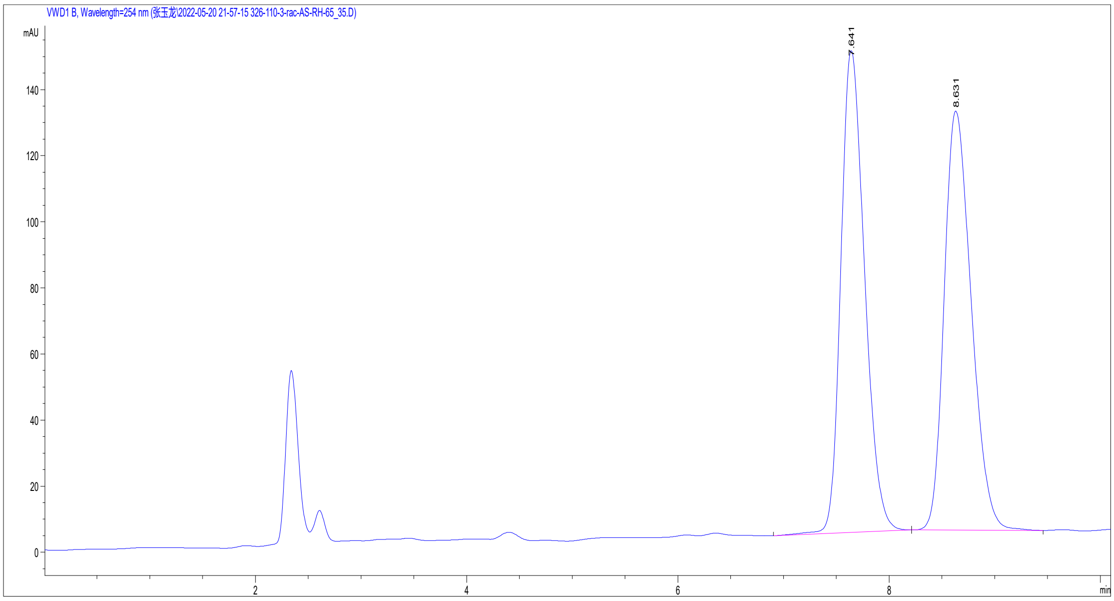

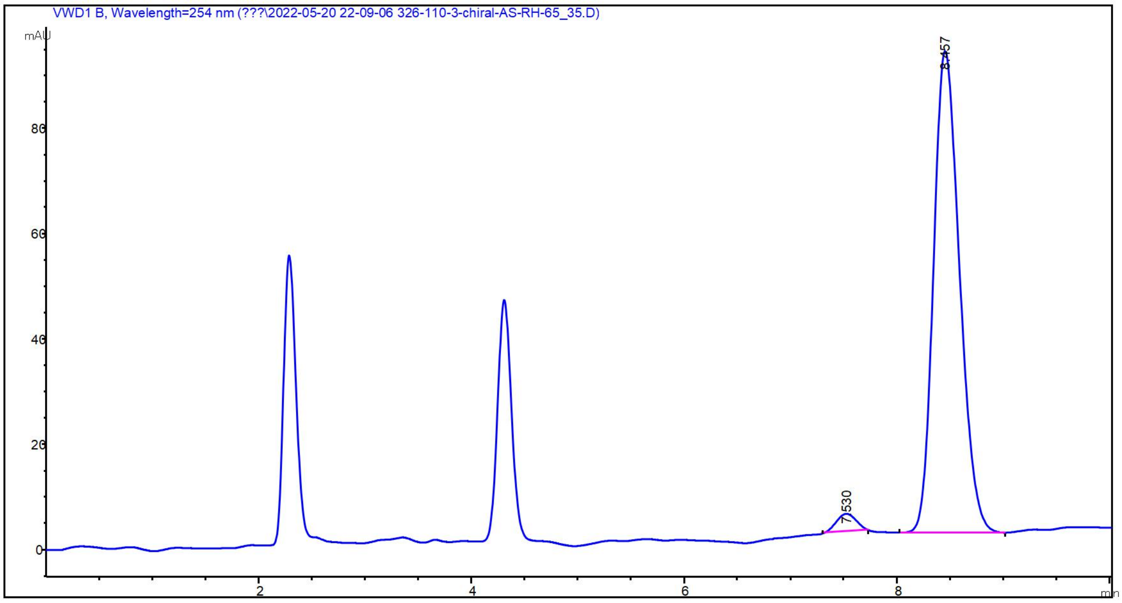

Index	t_R/ (min)	Height / (mv)	Area / (mv.sec)	Area / (%)
1	7.64	145.8	2276.3	50.2
2	8.63	126.8	2465.2	49.8
Total				100.0

Index	t_R/ (min)	Height / (mv)	Area / (mv.sec)	Area / (%)
1	7.53	40.6	40.6	2.5
2	8.45	1551.4	1551.4	97.5
Total				100.0

(iso-Butenyl)-phenyphosphinous acid-borane 5ag:

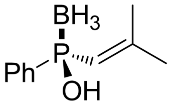

Enantiomeric excess: 91%. 178.4 mg (92% yield), colorless oil, R_f = 0.58 (PE/EA= 5:1), $[\alpha]_D^{25}$ = 8.3° (c = 1.00 in $CHCl_3$); ^1H NMR (300 MHz, $CDCl_3$) δ 7.94–7.63 (m, 2H), 7.47–7.45 (m, 3H), 5.68 (d, *J* = 15.6 Hz, 1H), 4.99 (bs, 1H), 1.93 (s, 6H), 1.34–0.39 (m, 3H); ^{13}C NMR (75 MHz, $CDCl_3$) δ 157.88 (d, *J* = 6.8 Hz), 134.36 (d, *J* = 65.3 Hz), 131.52 (d, *J* = 2.3 Hz), 130.42 (d, *J* = 11.3 Hz), 128.61 (d, *J* = 10.5 Hz), 117.31 (d, *J* = 66.8 Hz), 28.78 (d, *J* = 15.0 Hz), 21.93 (d, *J* = 7.5 Hz); ^{31}P NMR (122 MHz, $CDCl_3$) δ 89.76 (q, *J* = 192.8 Hz); HRMS (ESI-MS) $[M-H]^+$: found 194.1035; calculated for $C_{10}H_{15}BOP$: 194.1032.

Chiral HPLC: Chiralpak OJ-RH column, Water/Acetonitrile= 65/35, flow rate = 1.0 mL/min, λ = 254 nm.

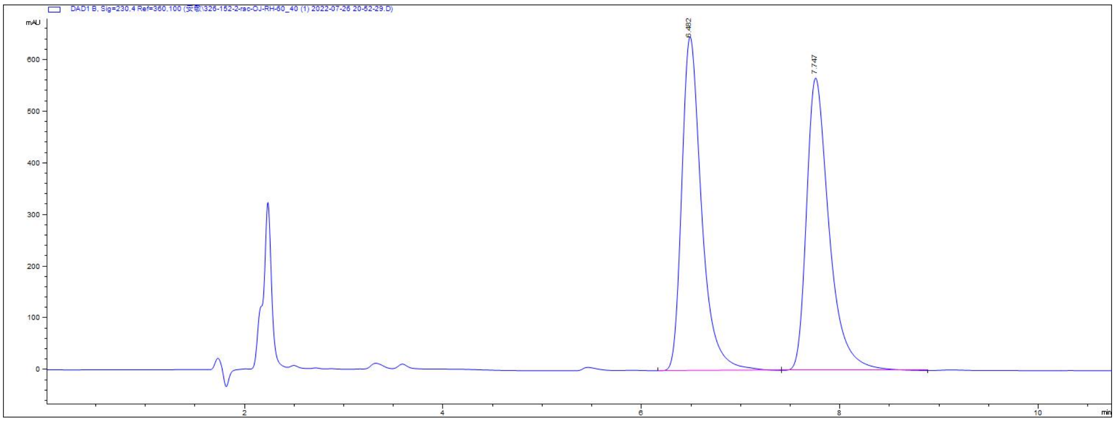

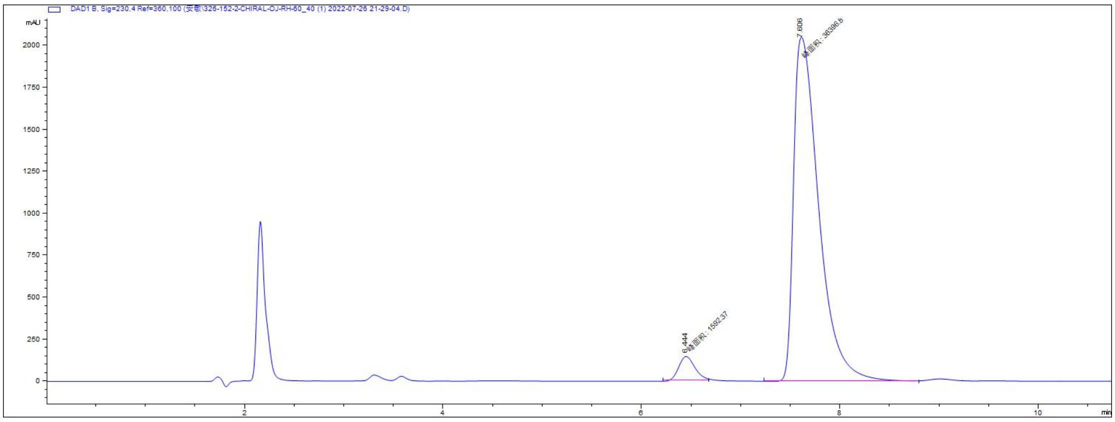

Index	t_R/ (min)	Height / (mv)	Area / (mv.sec)	Area / (%)
1	6.48	647.7	8996.4	50.0
2	7.74	567.0	8984.1	50.0
Total				100.0

Index	t_R/ (min)	Height / (mv)	Area / (mv.sec)	Area / (%)
1	6.44	145.8	1681.7	4.3
2	7.60	2058.6	36663.2	95.6
Total				100.0

Benzylphenyphosphinous acid-borane 5ah:

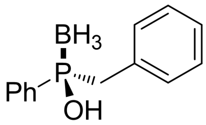

Enantiomeric excess: 95%. 193.2 mg (84% yield), colorless oil, R_f = 0.63 (PE/EA= 5:1), $[\alpha]_D^{25}$ = 17.3° (c = 1.00 in $CHCl_3$); ^1H NMR (300 MHz, $CDCl_3$) δ 7.66 – 7.55 (m, 2H), 7.49 (d, *J* = 6.9 Hz, 1H), 7.41 (t, *J* = 7.1 Hz, 2H), 7.21 (s, 3H), 7.02 (s, 2H), 3.30 (d, *J* = 10.1 Hz, 2H), 1.06–0.24 (m, 3H); ^{13}C NMR (75 MHz, $CDCl_3$) δ 132.23, 131.78 (d, *J* = 2.3 Hz), 131.42, 131.28 (d, *J* = 6.8 Hz), 130.47 (d, *J* = 11.3 Hz), 130.20 (d, *J* = 4.5 Hz), 128.64, 128.43 (d, *J* = 10.5 Hz), 128.25 (d, *J* = 3.8 Hz), 127.46, 126.96 (d, *J* = 3.0 Hz), 40.08 (d, *J* = 36.8 Hz); ^{31}P NMR (122 MHz, $CDCl_3$) δ 100.34 (q, *J* = 178.1 Hz); HRMS (ESI-MS) $[M-H]^+$: found 229.1035; calculated for $C_{13}H_{15}BOP$: 229.1032.

Chiral HPLC: Chiralpak AD-RH column, Water/Acetonitrile= 65/35, flow rate = 1.0 mL/min, λ = 254 nm.

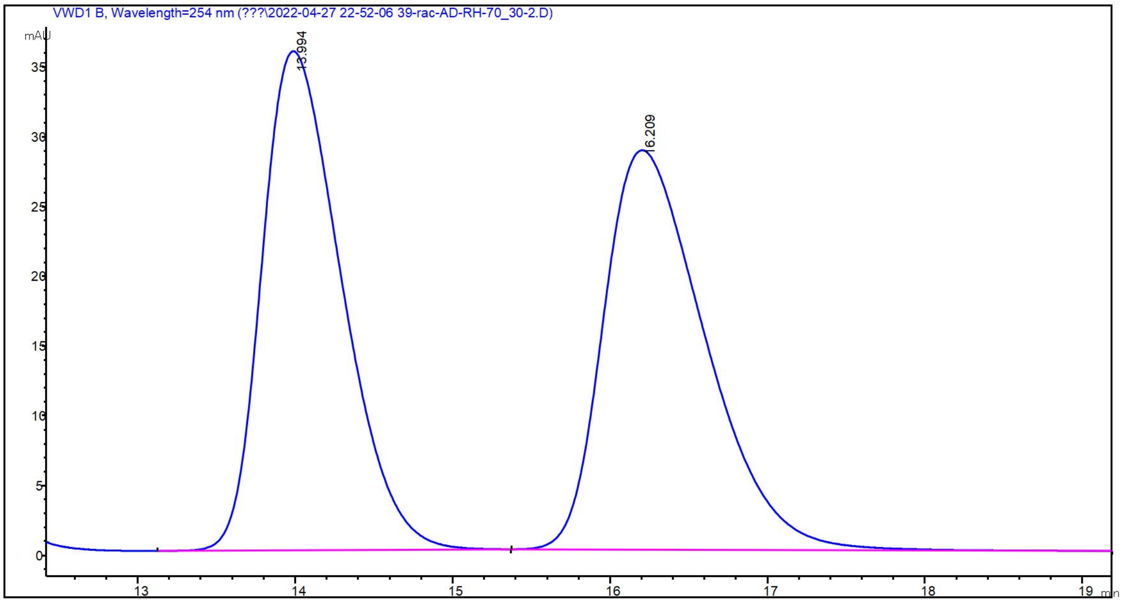

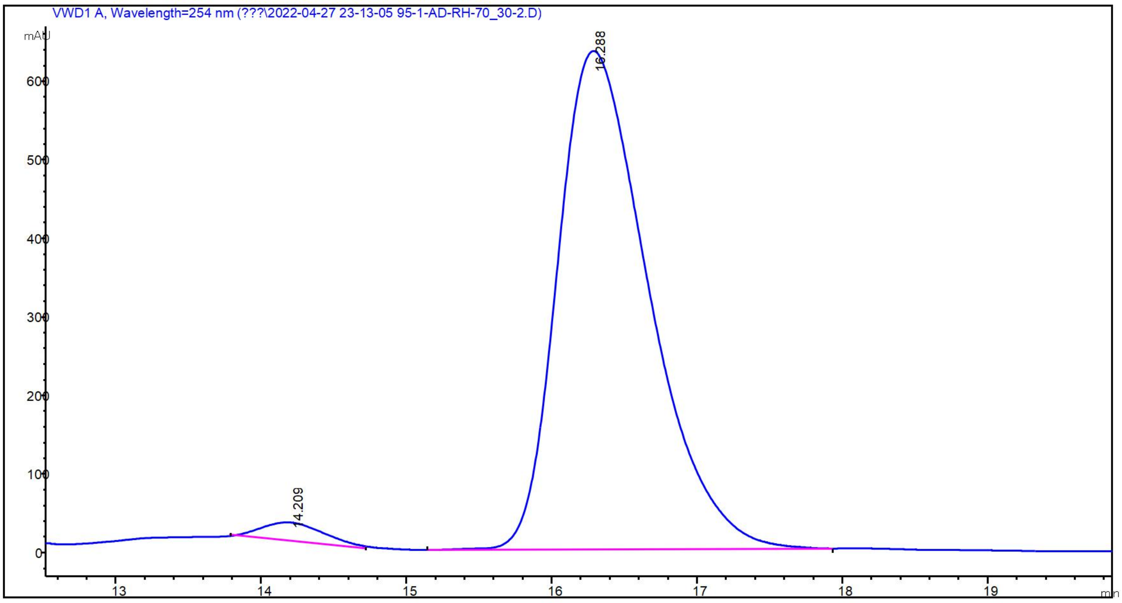

Index	t_R/ (min)	Height / (mv)	Area / (mv.sec)	Area / (%)
1	13.99	35.8	1256.7	49.4
2	16.20	28.6	1285.7	50.6
Total				100.0

Index	t_R/ (min)	Height / (mv)	Area / (mv.sec)	Area / (%)
1	14.20	23.2	687.5	2.4
2	16.28	634.3	27521.4	97.6
Total				100.0

Phenylethynylphenyphosphinous acid-borane 5ai:

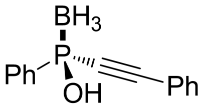

Enantiomeric excess: 80%. 133.4 mg (82% yield), colorless oil, R_f = 0.6 (PE/EA= 5:1), $[\alpha]_D^{25}$ = -30.4° (c = 1.00 in $CHCl_3$); ^1H NMR (600 MHz, $CDCl_3$) δ 8.04 – 7.94 (m, 2H), 7.88 (dd, J = 15.7, 7.2 Hz, 1H), 7.55 (dd, J = 11.2, 7.7 Hz, 3H), 7.51 (dd, J = 7.7, 2.6 Hz, 2H), 7.42 (t, J = 7.0 Hz, 1H), 7.40 – 7.32 (m, 2H); ^{13}C NMR (151 MHz MHz, $CDCl_3$) δ 133.33 (d, J = 2.9 Hz), 132.70 , 132.42 , 132.20 (d, J = 2.0 Hz), 131.18 , 130.74 (d, J = 12.8 Hz), 130.56 (d, J = 13.2 Hz), 130.45 , 129.08 (d, J = 14.6 Hz), 128.69 , 128.61 , 128.44; ^{31}P NMR (243 MHz, $CDCl_3$) δ 75.70 (q, J = 132.8 Hz); HRMS (ESI-MS) $[M-H]^+$: found 239.0878; calculated for $C_{14}H_{13}BOP$: 239.0875.

Chiral HPLC: Chiralpak OJ-RH column, Water/Acetonitrile= 60/40, flow rate = 1.0 mL/min, λ = 254 nm.

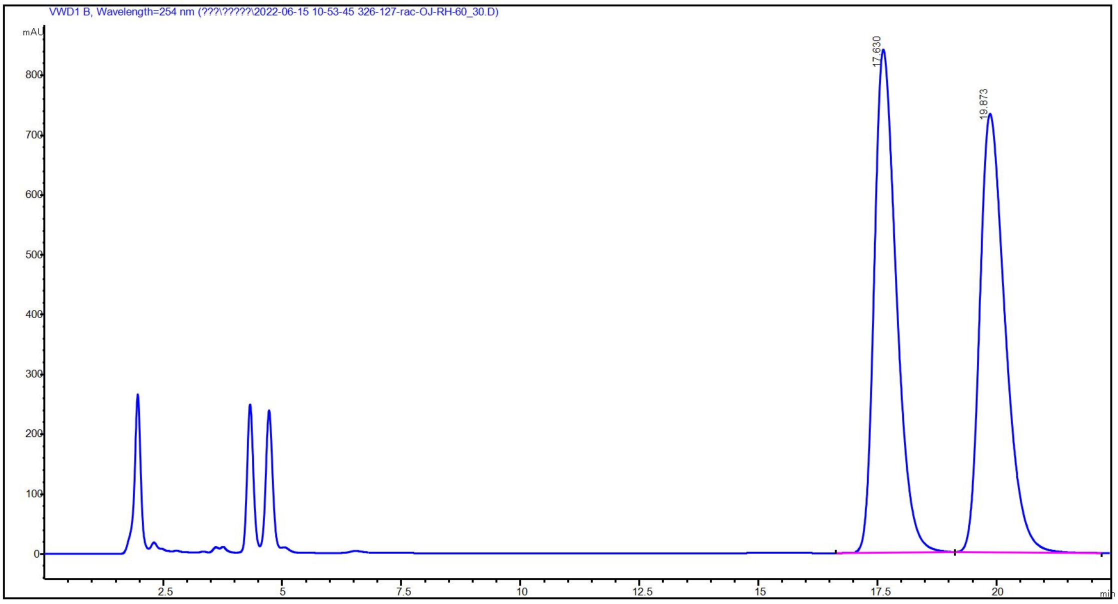

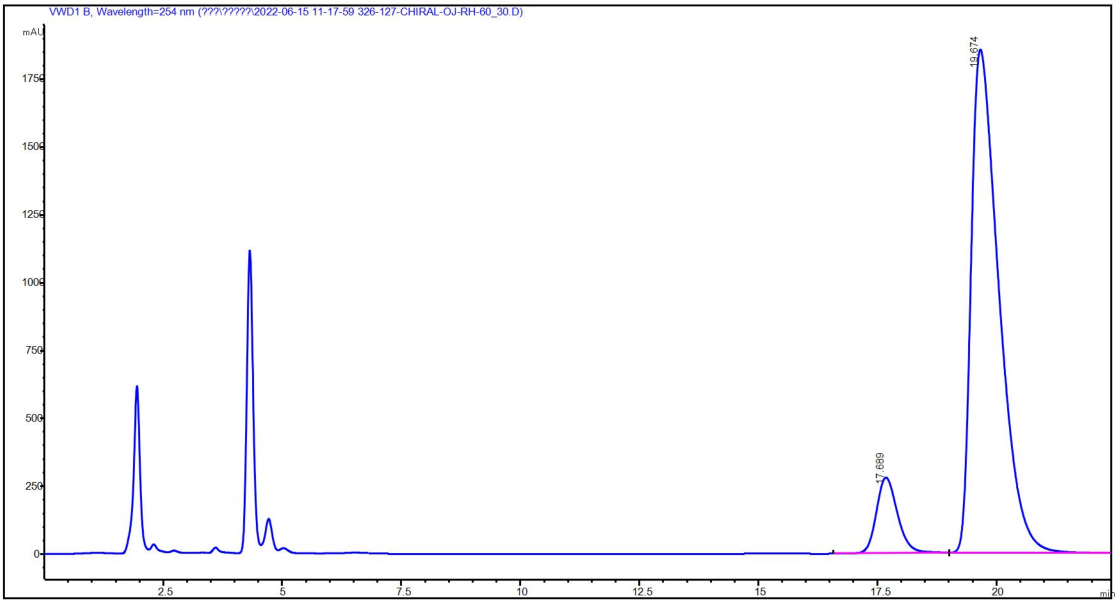

Index	t_R / (min)	Height / (mv)	Area /	Area / (%)
1	17.63	840.7	27078.9	50.0
2	19.87	732.6	27094.8	50.0
Total				100.0

Index	t_R / (min)	Height / (mv)	Area /	Area / (%)
1	17.68	278.2	8491.6	10.1
2	19.67	1853.5	75102.5	89.9
Total				100.0

***m*-Tolylphenyphosphinous acid-borane 5aj:**

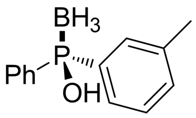

Enantiomeric excess: 75%. 133.4 mg (58% yield), white solid, **m.p.** = 145–147 °C, R_f = 0.42 (PE/EA= 5:1), $[\alpha]_D^{25}$ = -15.4° (c = 1.00 in $CHCl_3$); ^1H NMR (300 MHz, $CDCl_3$) δ 7.75–7.59 (m, 2H), 7.55–7.40 (m, 2H), 7.39–7.28 (m, 3H), 7.22 (s, 2H), 6.18 (bs, 1H), 2.27 (s, 3H), 1.38–0.64 (m, 3H); ^{13}C NMR (75 MHz, $CDCl_3$) δ 138.42 (d, J = 10.5 Hz), 133.54 (d, J = 31.5 Hz), 132.89, 132.57 (d, J = 2.3 Hz), 132.46, 131.65 (d, J = 2.3 Hz), 131.33 (d, J = 12.0 Hz), 130.94 (d, J = 12.0 Hz), 129.13, 128.95, 128.63 (d, J = 3.8Hz), 128.49 (d, J = 4.5 Hz), 128.14, 127.98, 21.52; ^{31}P NMR (122 MHz, $CDCl_3$) δ 93.89 (q, J = 87.8 Hz); HRMS (ESI-MS) $[M-H]^+$: found 229.1035; calculated for $C_{13}H_{15}BOP$: 229.1032.

Chiral HPLC: Chiralpak OJ-RH column, Water/Acetonitrile= 60/40, flow rate = 1.0 mL/min, λ = 254 nm.

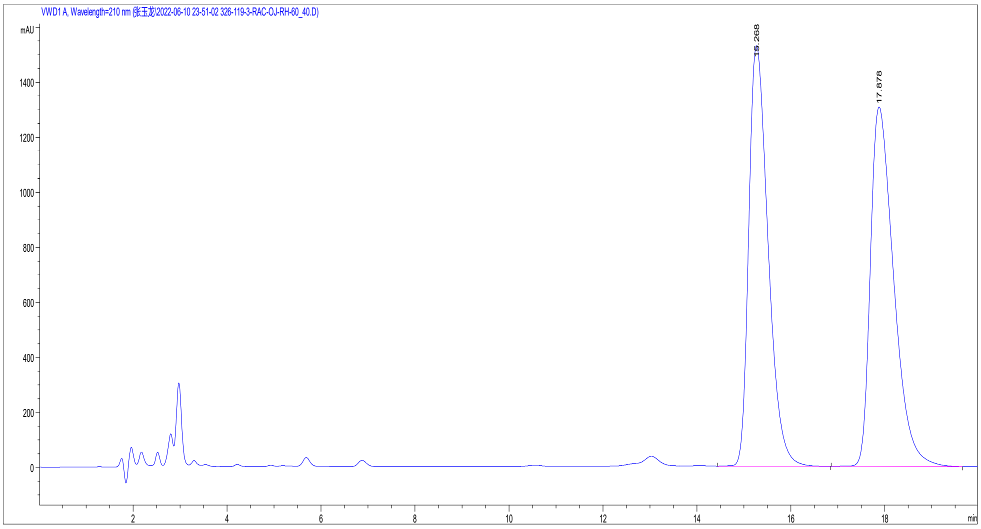

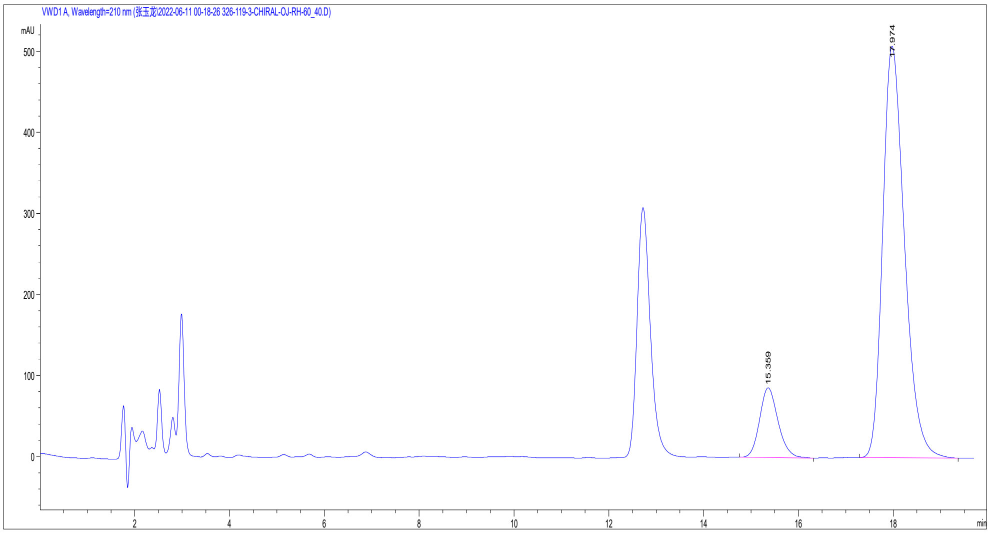

Index	t_R/ (min)	Height / (mv)	Area / (mv.sec)	Area / (%)
1	15.26	1529.3	43706.6	49.2
2	17.87	1306.8	450007.6	50.8
Total				100.0

Index	t_R/ (min)	Height / (mv)	Area / (mv.sec)	Area / (%)
1	15.35	86.2	2339.5	12.6
2	17.97	507.6	16218.2	87.4
Total				100.0

4-Methoxyphenylphenyphosphinous acid-borane 5ak:

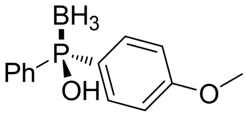

Enantiomeric excess: 83%. 196.8 mg (80% yield), white solid, **m.p.** = 103–108 °C, R_f = 0.48 (PE/EA= 5:1), $[\alpha]_D^{25}$ = -48.3° (c = 1.00 in $CHCl_3$); ^{1}H NMR (300 MHz, $CDCl_3$) δ 7.69 (q, *J* = 11.1, 10.7 Hz, 4H), 7.56–7.33 (m, 3H), 6.95 (d, *J* = 7.2 Hz, 2H), 5.31 (bs, 1H), 3.82 (s, 3H), 1.92–0.41 (m, 3H); ^{13}C NMR (75 MHz, $CDCl_3$) δ 162.34 (d, *J* = 2.3 Hz),133.15(d, *J* = 66.8 Hz), 133.14 (d, *J* = 13.5 Hz), 131.67 (d, *J* = 2.3 Hz), 130.80 (d, *J* = 12.0 Hz),128.62(d, *J* = 10.5 Hz),124.83 (d, *J* = 69.8 Hz), 115.7 (d, *J* = 91.5 Hz), 114.74 (d, *J* = 11.3 Hz),55.52; ^{31}P NMR (122 MHz, $CDCl_3$) δ 94.62 (q, *J* = 80.52 Hz); HRMS (ESI-MS) $[M\text{-}H]^+$: found 245.0909; calculated for $C_{13}H_{15}BO_2P$: 245.0904.

Chiral HPLC: Chiralpak AD-RH column, Water/Acetonitrile= 70/30, flow rate = 1.0 mL/min, λ = 254 nm.

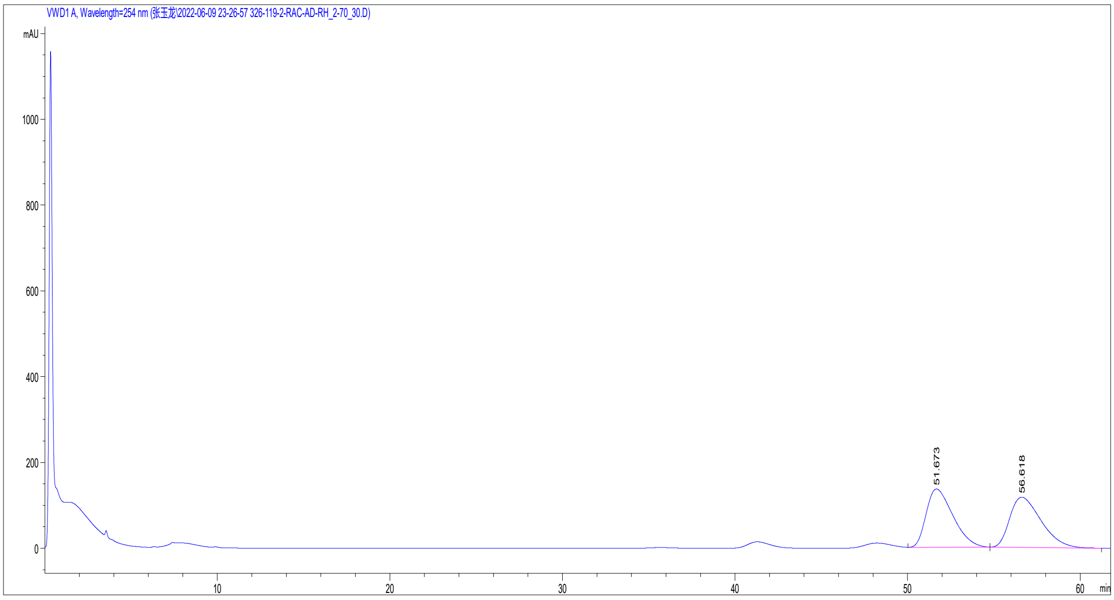

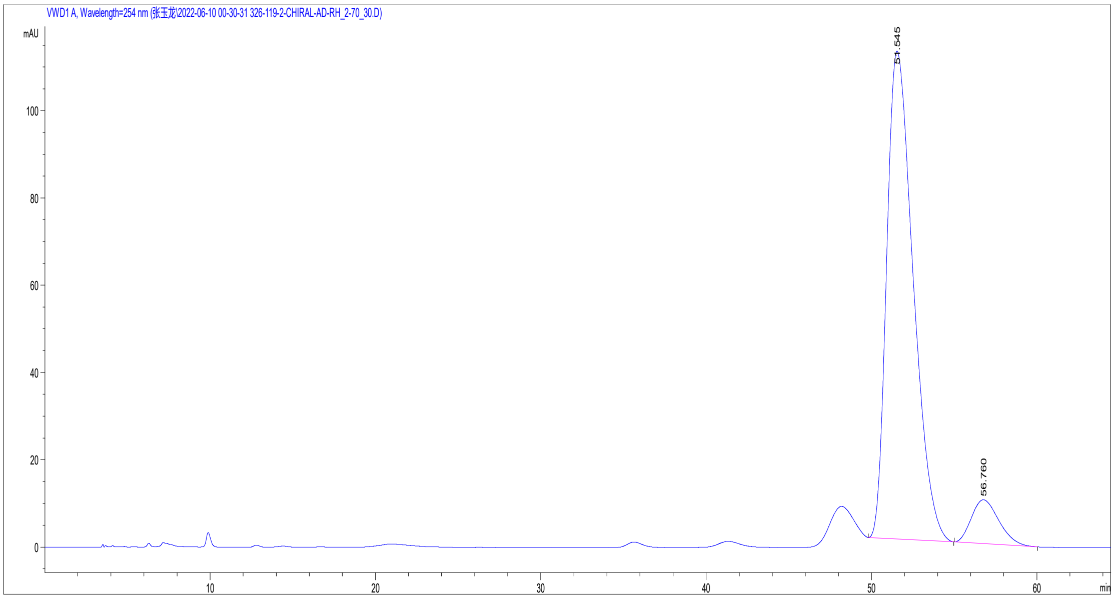

Index	t_R/ (min)	Height / (mv)	Area / (mv.sec)	Area / (%)
1	51.67	142.7	16606.6	49.8
2	56.68	129.2	17595.8	50.2

Total				100.0

Index	t_R/ (min)	Height / (mv)	Area / (mv.sec)	Area / (%)
1	51.54	111.8	12419.0	91.5
2	56.76	10	1159.7	8.5
Total				100.0

General procedure for synthesis of phosphinous acid-borane 5ba-br

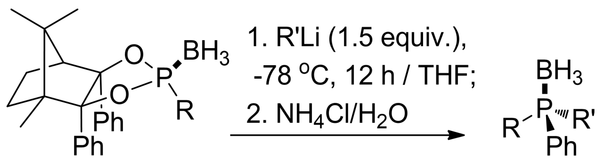

4ce; 4be **5b**

The organolithium reagent (1.5 mmol, 1.5 equiv.) in 14 mL of THF was cooled to -78 °C if not already at such temperature. A solution of the starting material (1.0 mmol, 1.0 equiv.) in 6 mL THF was prepared in a flame-dried flask under argon atmosphere, which was then added dropwise to the flask containing the organolithium reagent. The resulting mixture was stirred for 12 h while being kept at -78 °C. After ^{31}P NMR analysis of a small aliquot showed complete consumption of starting material, the reaction was warmed to room temperature. To the resulting mixture was added saturated aqueous NH_4Cl solution (20 mL) and EtOAc (40 mL). The layers were separated, and the aqueous layer was washed with EtOAc (2 x 20 mL). The combined organic layers were washed with brine (20 mL), dried over anhydrous Na_2SO_4, filtered and concentrated. The residue was purified by silica gel chromatography to afford the desired product.

Isopropylphenyphosphinous acid-borane 5ba:

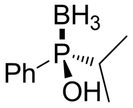

Enantiomeric excess: 97%. 152.9 mg (84% yield), Glassy solid, **m.p.** = 88–91 °C, R_f = 0.60 (PE/EA= 5:1), $[\alpha]_D^{25}$ = 27.8° (c = 1.00 in $CHCl_3$); ^{1}H NMR (300 MHz, $CDCl_3$) δ 7.74 (t, J = 8.4 Hz, 2H), 7.48 (q, J = 7.9 Hz, 3H), 2.23–1.96 (m, 1H), 1.18–1.00 (m,7H), 0.58–0.30 (m, 2H); ^{13}C NMR (75 MHz, $CDCl_3$) δ 131.60 (d, J = 2.3 Hz),130.76 (d, J = 10.5 Hz), 128.43 (d, J = 9.8 Hz), 29.33 (d, J = 42.7 Hz), 15.54, 15.33; ^{31}P NMR (122 MHz, $CDCl_3$) δ 109.6 (q, J = 200.1 Hz); HRMS (ESI-MS) $[M-H]^+$: found 182.1042; calculated for $C_9H_{15}BOP$: 181.1032.
Chiral HPLC: Chiralpak OJ-RH column, Water/Acetonitrile= 60/40, flow rate = 1.0 mL/min, λ = 254 nm.

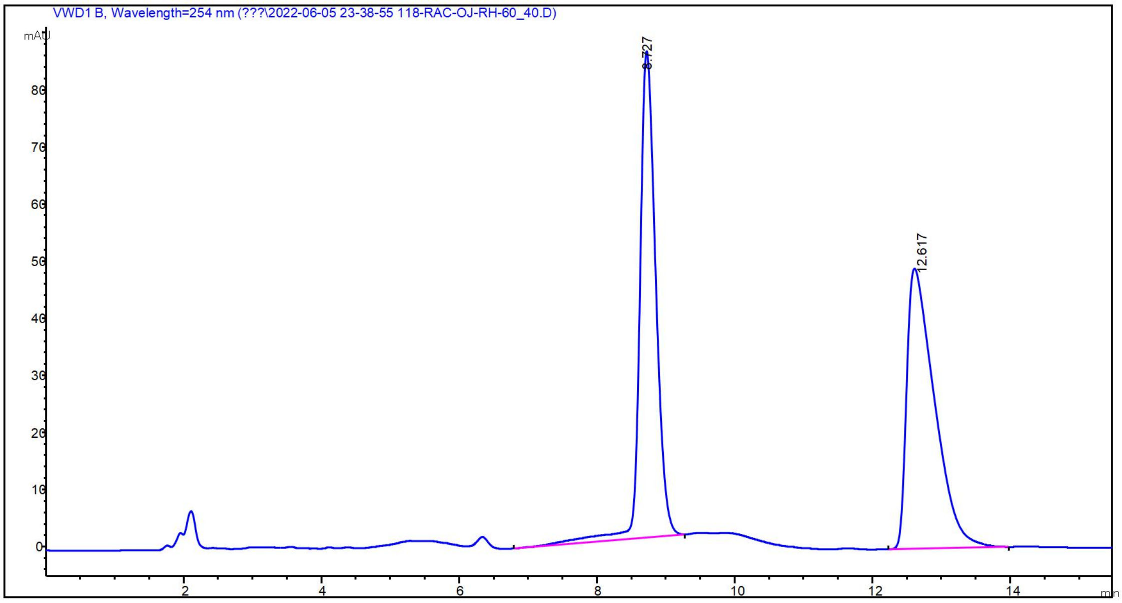

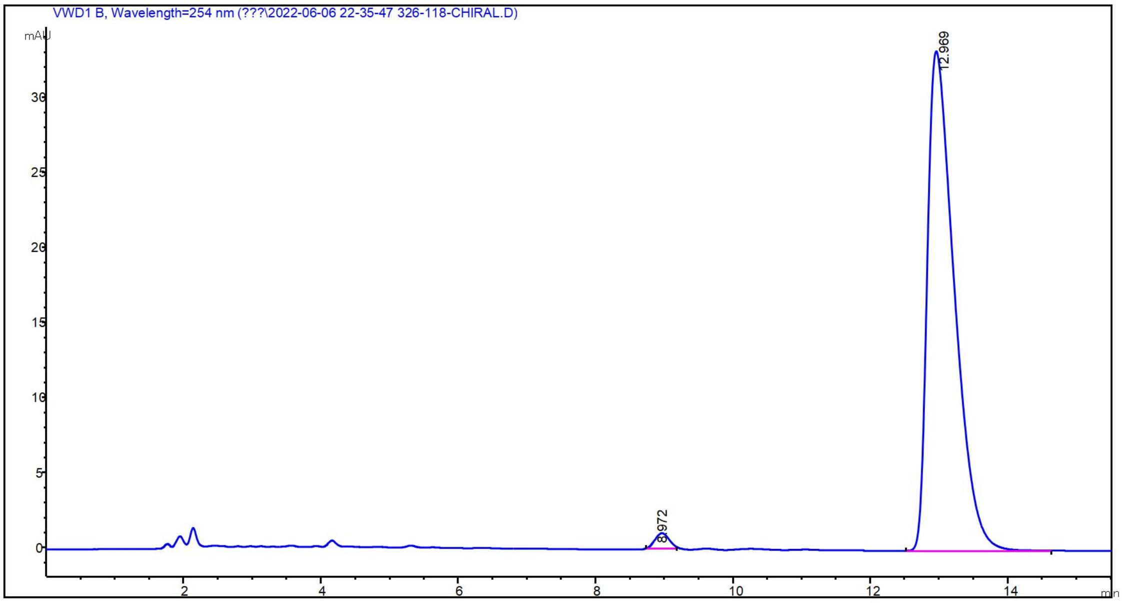

Index	t_R/ (min)	Height / (mv)	Area / (mv.sec)	Area / (%)
1	8.72	85.2	1381.7	50.1
2	12.61	49.1	1372.1	49.8
Total				100.0

Index	t_R/ (min)	Height / (mv)	Area / (mv.sec)	Area / (%)
1	8.97	0.99	13.8	1.5
2	12.96	33.3	904.4	98.5
Total				100.0

Tert-butylphenyphosphinous acid-borane 5bb:

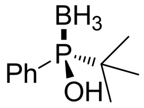

Enantiomeric excess: 93%. 174.4 mg (89% yield), Glassy solid, **m.p.** = 93–98 °C, R_f = 0.65 (PE/EA= 5:1), $[\alpha]_D^{25}$ = 8.4° (c = 1.00 in $CHCl_3$); ^1H NMR (300 MHz, $CDCl_3$) δ 7.65 (t, J = 8.3 Hz, 2H), 7.43–7.37 (m, 3H), 1.03 (d, J = 14.8 Hz, 9H); ^{13}C NMR (75 MHz, $CDCl_3$) δ 131.54, 131.42 (d, J = 2.3 Hz), 130.68, 129.94, 128.09 (d, J = 9.8 Hz), 31.81 (d, J = 41.3 Hz), 23.3 (d, J = 3.8 Hz); ^{31}P NMR (122 MHz, $CDCl_3$) δ 112.62 (q, J = 200.1 Hz); HRMS (ESI-MS) $[M-H]^+$: found 195.1190; calculated for $C_{10}H_{17}BOP$: 195.1188.

Chiral HPLC: Chiralpak AD-RH column, Water/Acetonitrile= 60/40, flow rate = 1.0 mL/min, λ = 254 nm.

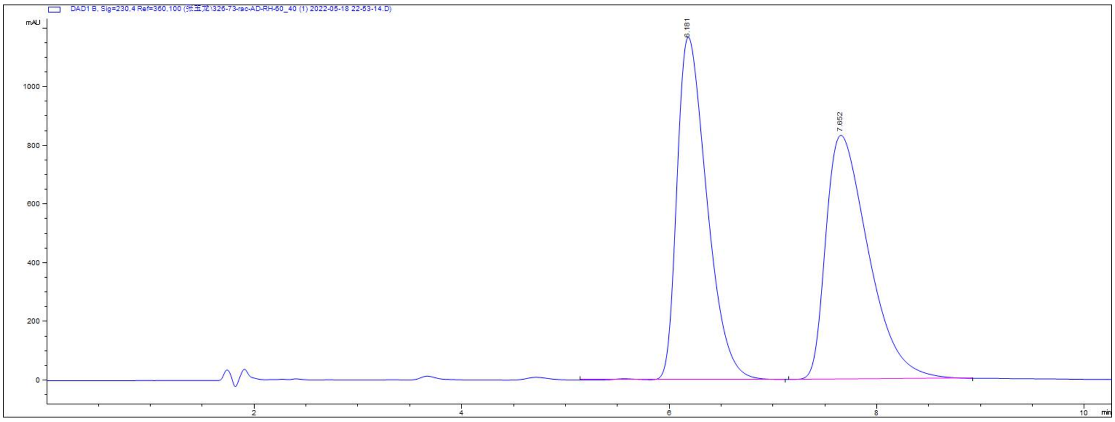

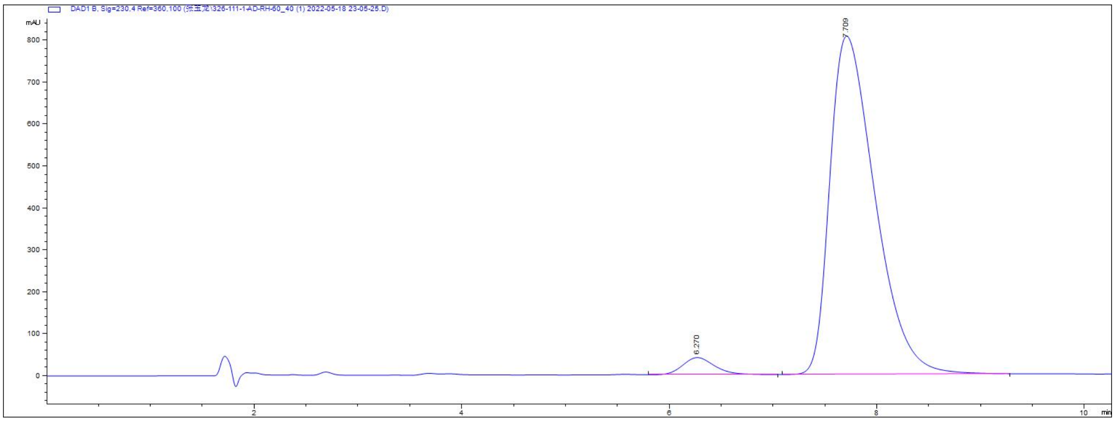

Index	t_R/ (min)	Height / (mv)	Area / (mv.sec)	Area / (%)
1	6.18	1170.1	23432.7	49.6
2	7.65	832.8	23717.4	50.4
Total				100.0

Index	t_R/ (min)	Height / (mv)	Area / (mv.sec)	Area / (%)
1	6.27	40.9	873.2	3.4
2	7.70	806.0	24319.2	96.6
Total				100.0

***o*-Tolylphenyphosphinous acid-borane 5bc:**

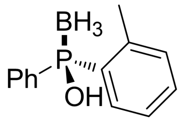

Enantiomeric excess: 97%. 213.9 mg (93% yield), white solid, **m.p.** = 113–118 °C, R_f = 0.38 (PE/EA= 5:1), $[\alpha]_D^{25}$ = -28.9° (c = 1.00 in $CHCl_3$); ^1H NMR (300 MHz, $CDCl_3$) δ 7.98 (dd, *J* = 13.0, 7.6 Hz, 1H), 7.75–7.59 (m, 2H), 7.47 (dd, *J* = 15.3, 7.1 Hz, 4H), 7.35 (t, *J* = 7.3 Hz, 1H), 7.25–7.14 (m, 1H), 4.75 (bs, 1H), 2.24 (s, 3H), 1.49–0.56 (m, 3H); ^{13}C NMR (75 MHz, $CDCl_3$) δ 141.62 (d, *J* = 9.0 Hz), 133.10 (d, *J* = 11.3 Hz), 132.93 (d, *J* = 14.3 Hz), 132.35 (d, *J* = 2.3 Hz), 131.80 (d, *J* = 2.3 Hz), 131.57 (d, *J* = 9.0 Hz), 130.88 (d, *J* = 12.0 Hz), 130.44 (d, *J* = 54.0 Hz), 128.68 (d, *J* = 10.5 Hz), 125.84 (d, *J* = 11.3 Hz), 21.40 (d, *J* = 4.5 Hz); ^{31}P NMR (122 MHz, $CDCl_3$) δ 97.44 (q, *J* = 181.8 Hz); HRMS (ESI-MS) $[M-H]^+$: found 229.1036; calculated for $C_{13}H_{15}BOP$: 229.1032.

Chiral HPLC: Chiralpak OJ-RH column, Water/Acetonitrile= 60/40, flow rate = 1.0 mL/min, λ = 254 nm.

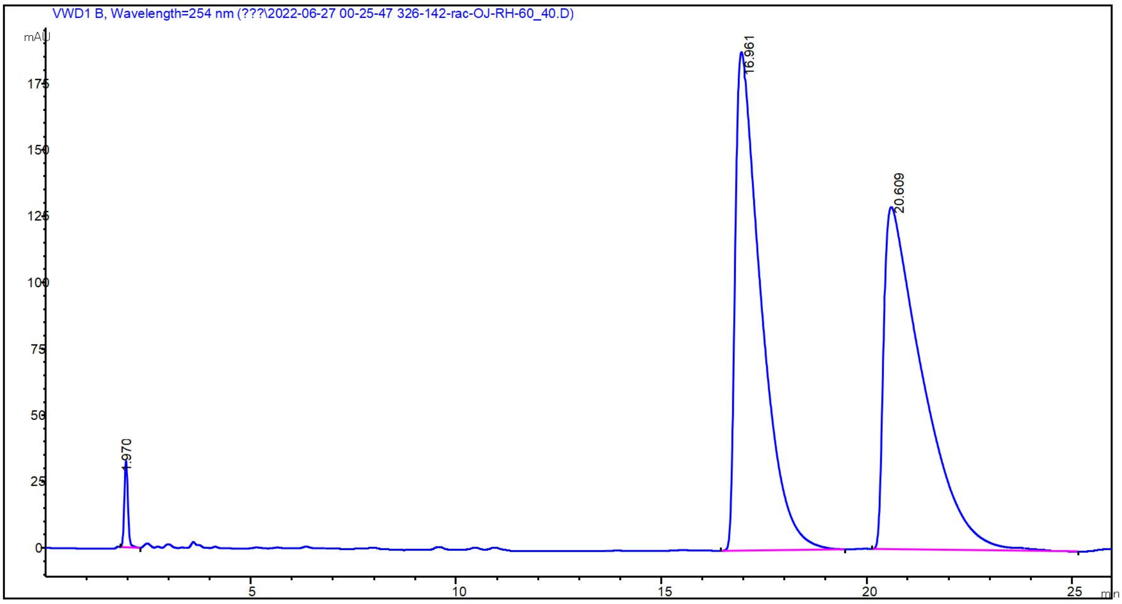

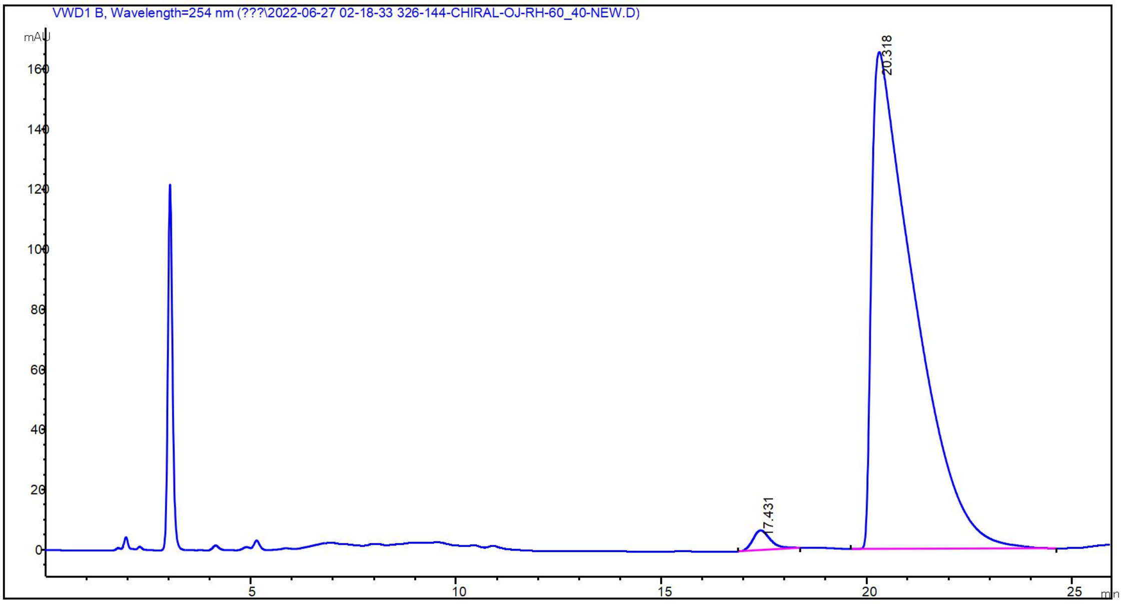

Index	t_R/ (min)	Height / (mv)	Area / (mv.sec)	Area / (%)
1	16.96	187.8	8229.0	49.2
2	20.60	128.9	8500.1	50.8
Total				100.0

Index	t_R/ (min)	Height / (mv)	Area / (mv.sec)	Area / (%)
1	17.43	6.5	189.9	1.5
2	20.31	165.5	11982.7	98.5
Total				100.0

***m*-Tolylphenyphosphinous acid-borane 5bd:**

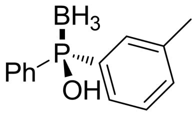

Enantiomeric excess: 98%. 198.9 mg (93% yield), white solid, **m.p.** = 149–153 °C, R_f = 0.42 (PE/EA= 5:1), $[\alpha]_D^{25}$ = -30.4° (c = 1.00 in $CHCl_3$); ^{1}H NMR (300 MHz, $CDCl_3$) δ 7.75–7.59 (m, 2H), 7.55–7.40 (m, 2H), 7.39–7.28 (m, 3H), 7.22 (s, 2H), 6.18 (bs, 1H), 2.27 (s, 3H), 1.38–0.64 (m, 3H); ^{13}C NMR (75 MHz, $CDCl_3$) δ 138.42 (d, *J* = 10.5 Hz), 133.54 (d, *J* = 31.5 Hz), 132.89, 132.57 (d, *J* = 2.3 Hz), 132.46, 131.65 (d, *J* = 2.3 Hz), 131.33 (d, *J* = 12.0 Hz), 130.94 (d, *J* = 12.0 Hz), 129.13, 128.95, 128.63 (d, *J* = 3.8Hz), 128.49 (d, *J* = 4.5 Hz), 128.14, 127.98, 21.52; ^{31}P NMR (122 MHz, $CDCl_3$) δ 93.89 (q, *J* = 87.8 Hz); HRMS (ESI-MS) $[M-H]^+$: found 229.1035; calculated for $C_{13}H_{15}BOP$: 229.1032.

Chiral HPLC: Chiralpak OJ-RH column, Water/Acetonitrile= 60/40, flow rate = 1.0 mL/min, λ = 254 nm.

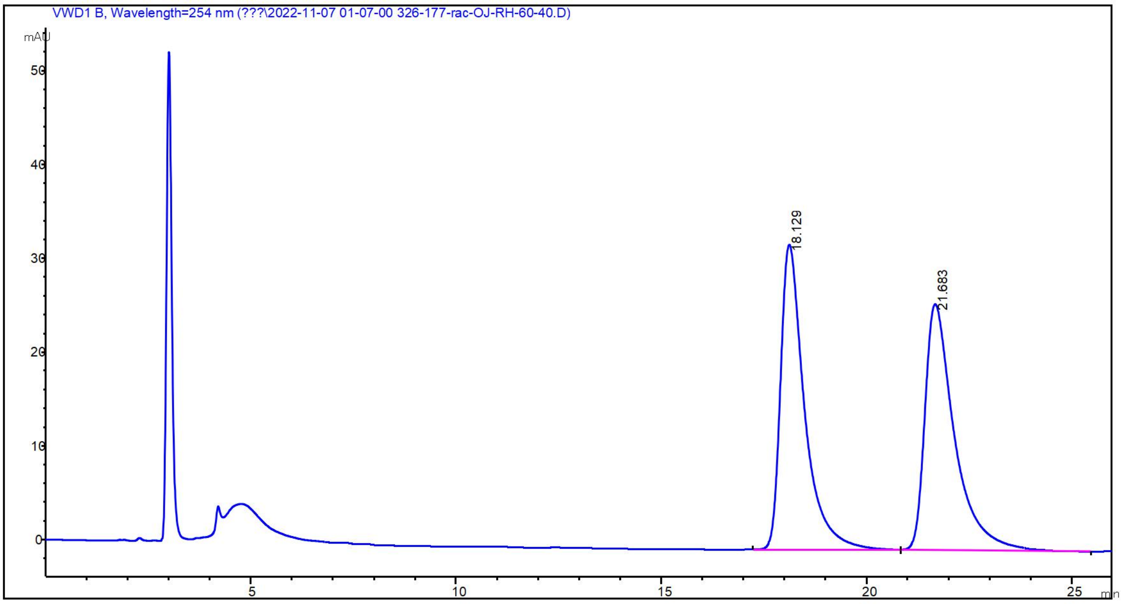

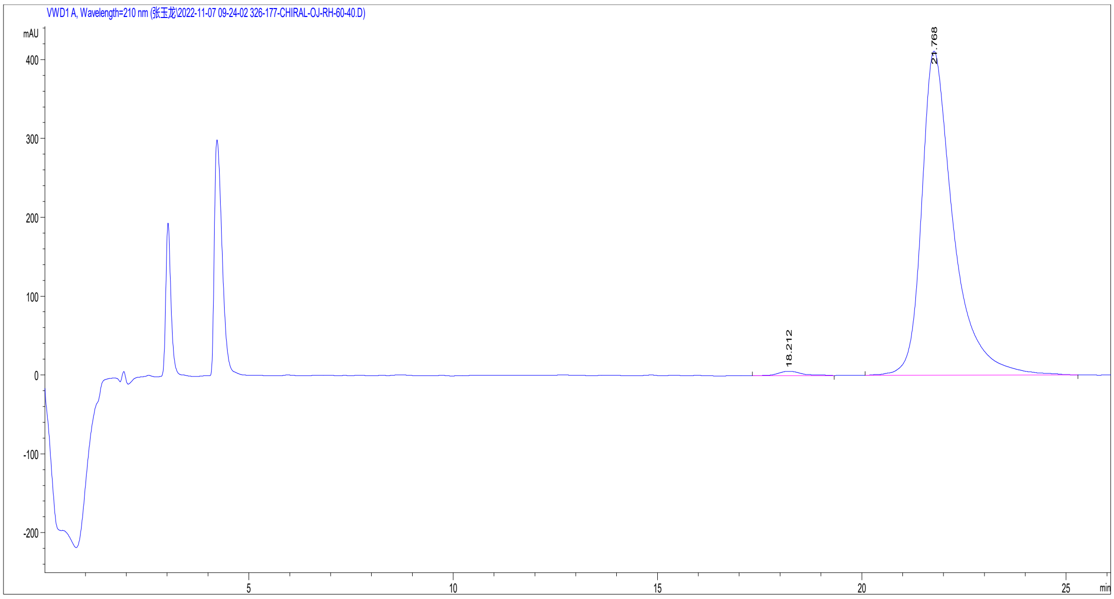

Index	t_R/ (min)	Height / (mv)	Area / (mv.sec)	Area / (%)
1	18.12	32.5	1318.0	50.0
2	21.68	26.2	1315.6	50.0
Total				100.0

Index	t_R/ (min)	Height / (mv)	Area / (mv.sec)	Area / (%)
1	18.21	5.5	220.0	1.0
2	21.76	411	22227.9	99.0
Total				100.0

p-Tolylphenyphosphinous acid-borane 5be:

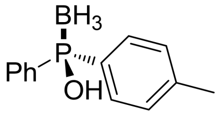

Enantiomeric excess: 99%. 213.9 mg (93% yield), white solid, **m.p.** = 113–118 °C, R_f = 0.38 (PE/EA= 5:1), $[\alpha]_D^{25}$ = -28.9° (c = 1.00 in $CHCl_3$); ^1H NMR (300 MHz, $CDCl_3$) δ 7.78–7.66 (m, 2H), 7.66–7.54 (m, 2H), 7.47 (dd, J = 18.2, 7.3 Hz, 3H), 7.25 (d, J = 7.0 Hz, 2H), 4.83 (bs, 1H), 2.37 (s, 3H), 1.71–0.49 (m, 3H); ^{13}C NMR (75 MHz, $CDCl_3$) δ 142.49 (d, J = 2.3 Hz), 132.91 (d, J = 66.0 Hz), 131.75 (d, J = 2.3 Hz), 131.07 (d, J =12.8 Hz), 130.98(d, J = 0.8 Hz), 130.81, 129.60 (d, J = 13.5 Hz), 129.44 (d, J = 10.5 Hz), 128.79, 128.63 (d, J = 11.3 Hz), 21.66; ^{31}P NMR (122 MHz, $CDCl_3$) δ 95.50 (q, J =181.78 Hz); HRMS (ESI-MS) $[M-H]^+$: found229.1038; calculated for $C_{13}H_{15}BOP$: 229.1032.

Chiral HPLC: Chiralpak OJ-RH column, Water/Acetonitrile= 60/40, flow rate = 1.0 mL/min, λ = 254 nm.

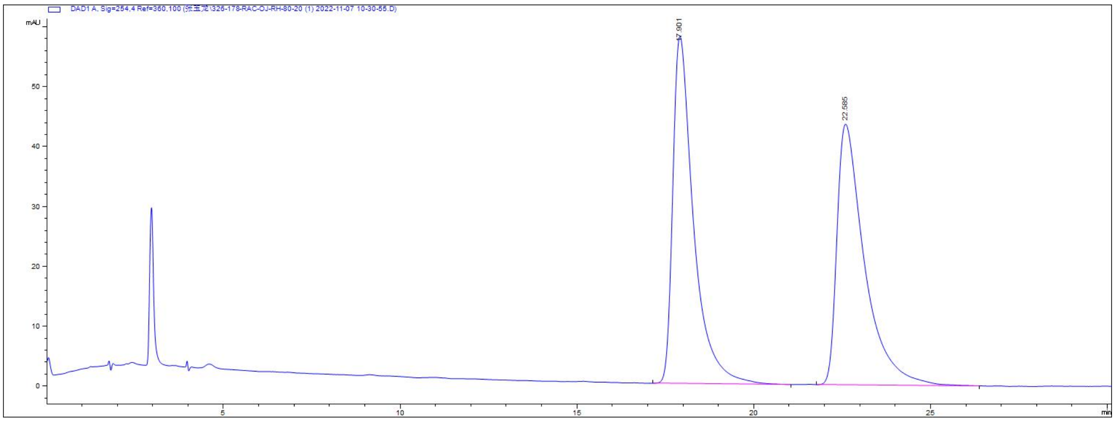

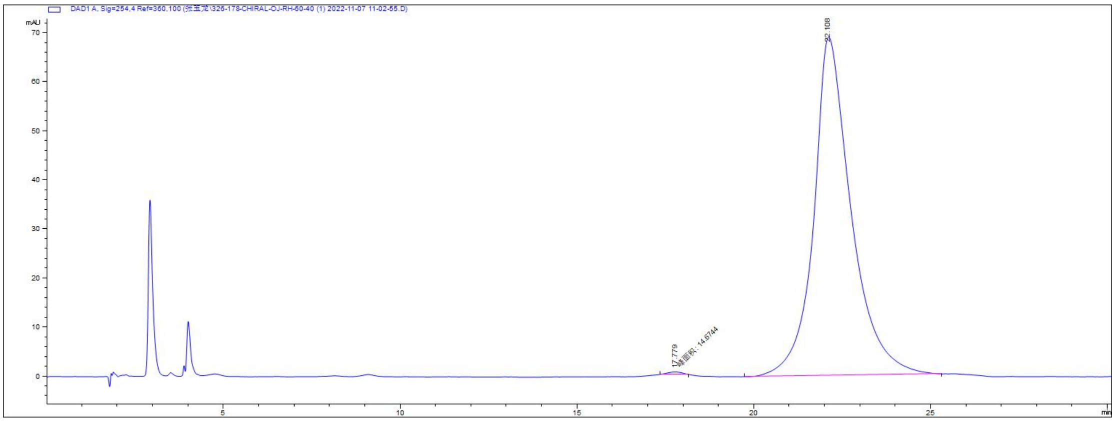

Index	t_R/ (min)	Height / (mv)	Area / (mv.sec)	Area / (%)
1	17.90	58	2372.4	50.1
2	22.58	43.5	2362.0	49.9
Total				100.0

Index	t_R/ (min)	Height / (mv)	Area / (mv.sec)	Area / (%)
1	17.77	0.52	14.7	0.3
2	22.10	69.1	5098.3	99.7
Total				100.0

***o*-Methoxyphenylphenyphosphinous acid-borane 5bf:**

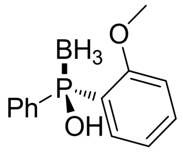

Enantiomeric excess: 99%. 210.1 mg (86% yield), white solid, **m.p.** = 98–102 °C, R_f = 0.45 (PE/EA= 5:1), $[\alpha]_D^{25}$ = -83.7° (c = 1.00 in $CHCl_3$); ^{1}H NMR (300 MHz, $CDCl_3$) δ 7.87 (dd, *J* = 13.4, 7.5 Hz, 1H), 7.62 (dd, *J* = 11.0, 7.1 Hz, 2H), 7.53 (d, *J* = 7.8 Hz, 1H), 7.42 (q, *J* = 8.0, 6.3 Hz, 3H), 7.13 (t, *J* = 7.4 Hz, 1H), 6.95 (dd, *J* = 8.3, 3.2 Hz, 1H), 5.45 (bs, 1H), 3.77 (s, 3H), 1.70–0.30 (m, 3H); ^{13}C NMR (75 MHz, $CDCl_3$) δ 160.63, 134.42 (d, *J* = 3.0 Hz),134.32 (d, *J* = 7.5 Hz), 134.23, 133.44, 131.33 (d, *J* = 2.3 Hz), 130.18 (d, *J* = 12.0 Hz), 128.42 (d, *J* = 10.5 Hz), 121.77 (d, *J* = 12.8 Hz), 119.61 (d, *J* = 56.3 Hz), 111.42 (d, *J* = 4.5 Hz), 56.09; ^{31}P NMR (122 MHz, $CDCl_3$) δ 96.95 (q, *J* =200.1 Hz); HRMS (ESI-MS) $[M-H]^+$: found 245.0499; calculated for $C_{13}H_{15}BO_2P$: 245.0497.

Chiral HPLC: Chiralpak OJ-RH column, Water/Acetonitrile= 60/40, flow rate = 1.0 mL/min, λ = 254 nm.

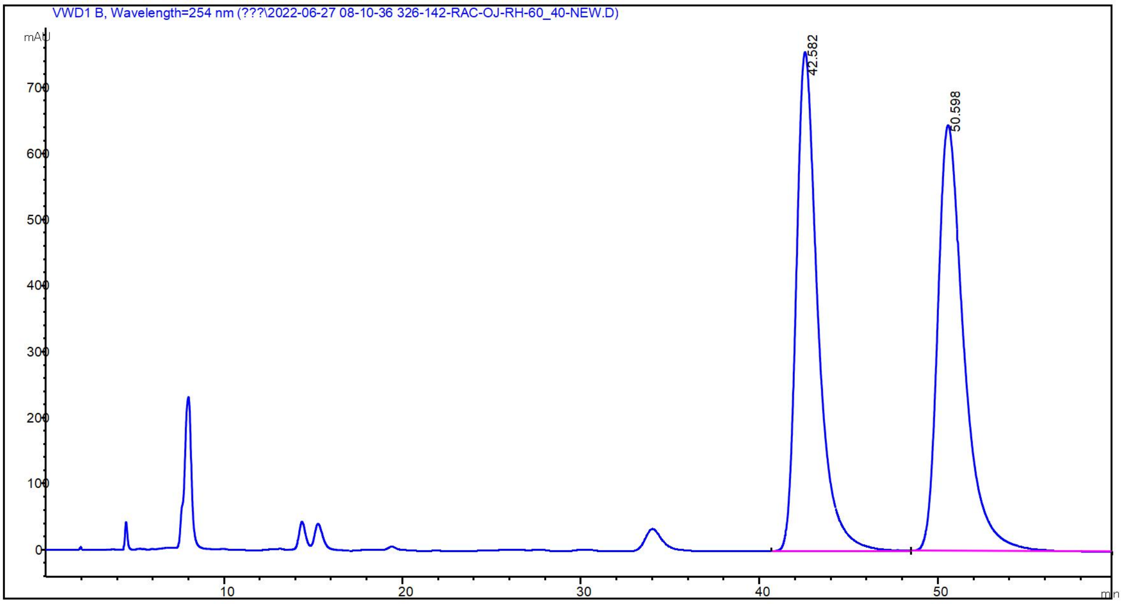

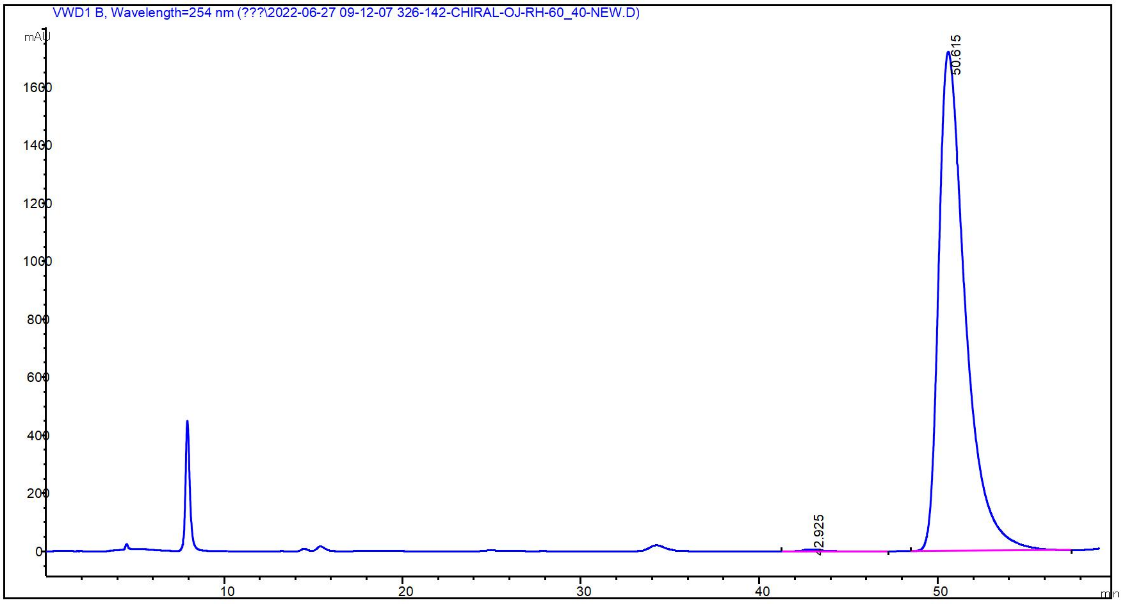

Index	t_R/ (min)	Height / (mv)	Area / (mv.sec)	Area / (%)
1	42.58	755.6	63434.2	49.1
2	50.59	644.4	65582.7	50.9
Total				100.0

Index	t_R/ (min)	Height / (mv)	Area / (mv.sec)	Area / (%)
1	42.92	6.7	554.6	0.3
2	50.61	1719.2	179452.1	99.7
Total				100.0

***m*-Methoxyphenylphenyphosphinous acid-borane 5bg:**

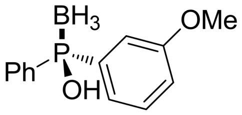

Enantiomeric excess: 99%. 204.2 mg (83% yield), white solid, **m.p.** = 80-84 °C, R_f = 0.45 (PE/EA= 5:1), **$[\alpha]^{25}_D$**= -52.3° (c = 1.00 in $CHCl_3$); ^{1}H NMR (300 MHz, $CDCl_3$) δ 7.83–7.60 (m, 2H), 7.52–7.35 (m, 3H), 7.27 (d, *J* = 15.9 Hz, 3H), 6.99 (d, *J* = 5.8 Hz, 1H), 5.67 (bs, 1H), 3.76 (s, 3H), 1.07 (d, *J* = 116.8 Hz, 3H); ^{13}C NMR (75 MHz, $CDCl_3$) δ 159.26 (d, *J* = 13.5 Hz), 134.4 (d, *J* = 64.5 Hz), 133.38 (d, *J* = 65.25 Hz), 131.80 (d, *J* = 2.3 Hz),130.90 (d, *J* = 12.0 Hz), 129.96 (d, *J* = 12.0 Hz), 128.64 (d, *J* = 11.3 Hz), 123.34 (d, *J* = 12.0 Hz), 117.72, 115.88 (d, *J* = 12.8 Hz), 55.55; ^{31}P NMR (122 MHz, $CDCl_3$) δ 94.31 (q, *J* =92.72 Hz); HRMS (ESI-MS) $[M-H]^+$: found 245.0497; calculated for $C_{13}H_{15}BO_2P$: 245.0497.

Chiral HPLC: Chiralpak OJ-RH column, Water/Acetonitrile= 60/40, flow rate = 1.0 mL/min, λ = 254 nm.

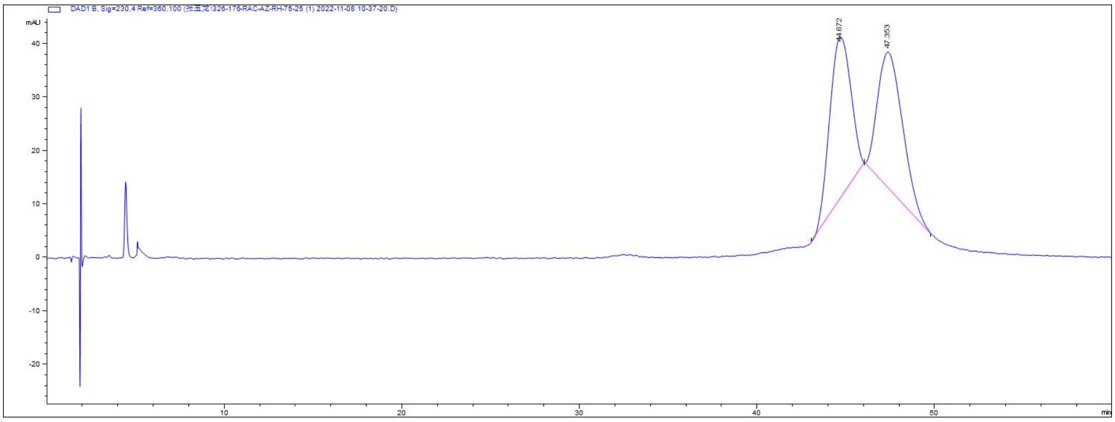

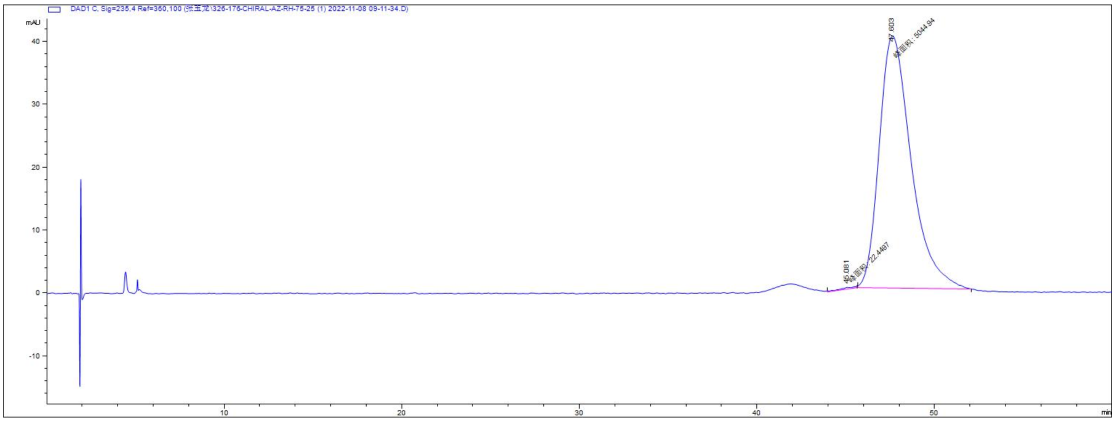

Index	t_R/ (min)	Height / (mv)	Area / (mv.sec)	Area / (%)
1	44.67	30.4	2532.3	50.4
2	47.35	25.5	2493.6	49.6
Total				100.0

Index	t_R/ (min)	Height / (mv)	Area / (mv.sec)	Area / (%)
1	45.04	0.43	31.9	0.4
2	47.60	58.5	7438.4	99.6
Total				100.0

***p*-Methoxyphenylphenyphosphinous acid-borane 5bh:**

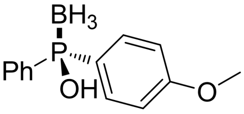

Enantiomeric excess: 98%. 245 mg (83% yield), white solid, **m.p.** = 103–108 °C, R_f = 0.48 (PE/EA= 5:1), $[\alpha]_D^{25}$ = -62.3° (c = 1.00 in $CHCl_3$); ^1H NMR (300 MHz, $CDCl_3$) δ 7.69 (q, *J* = 11.1, 10.7 Hz, 4H), 7.56–7.33 (m, 3H), 6.95 (d, *J* = 7.2 Hz, 2H), 5.31 (bs, 1H), 3.82 (s, 3H), 1.92–0.41 (m, 3H); ^{13}C NMR (75 MHz, $CDCl_3$) δ 162.34 (d, *J* = 2.3 Hz),133.15 (d, *J* = 66.8 Hz), 133.14 (d, *J* = 13.5 Hz), 131.67 (d, *J* = 2.3 Hz), 130.80 (d, *J* = 12.0 Hz), 128.62 (d, *J* = 10.5 Hz), 124.83 (d, *J* = 69.8 Hz), 115.7 (d, *J* = 91.5 Hz), 114.74 (d, *J* = 11.3 Hz), 55.52; ^{31}P NMR (122 MHz, $CDCl_3$) δ

94.62 (q, J = 80.52 Hz); HRMS (ESI-MS) $[M-H]^+$: found 245.0906; calculated for $C_{13}H_{15}BO_2P$: 245.0904.

Chiral HPLC: Chiralpak OJ-RH column, Water/Acetonitrile= 60/40, flow rate = 1.0 mL/min, λ = 254 nm.

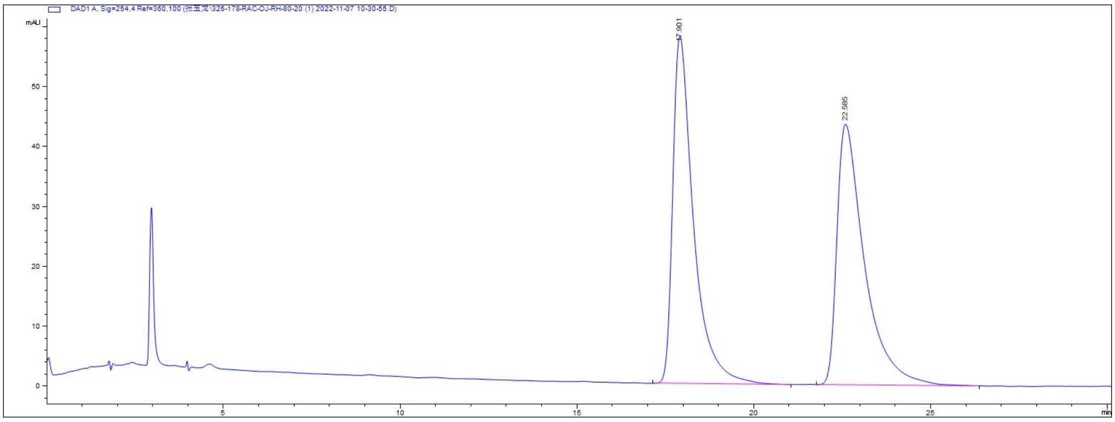

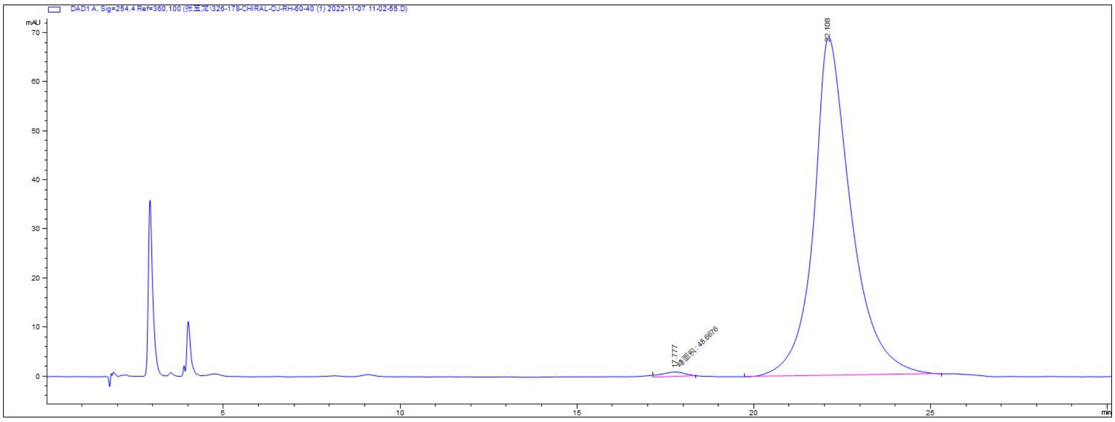

Index	t_R/ (min)	Height / (mv)	Area / (mv.sec)	Area / (%)
1	17.90	58.0	2372.4	50.1
2	22.58	43.5	2362.0	49.9
Total				100.0

Index	t_R/ (min)	Height / (mv)	Area / (mv.sec)	Area / (%)
1	17.77	1.0	48.7	0.9
2	22.10	69.1	5098.2	99.1
Total				100.0

***o*-Vinylphenylphenyphosphinous acid-borane 5bi:**

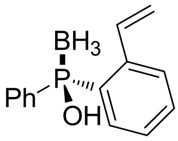

Enantiomeric excess: 99%. 213.1 mg (88% yield), white solid, **m.p.** = 123–127 °C, R_f = 0.33

(PE/EA= 5:1), $[\alpha]_D^{25}$ = -62.3° (c = 1.00 in $CHCl_3$); ^{1}H NMR (300 MHz, $CDCl_3$) δ 8.00 (dd, *J* = 13.2, 7.7 Hz, 1H), 7.68–7.57 (m, 3H), 7.52 (dd, *J* = 15.0, 7.4 Hz, 2H), 7.48–7.39 (m, 3H), 7.00 (dd, *J* = 17.1, 10.9 Hz, 1H), 5.54 (d, *J* = 17.1 Hz, 1H), 5.13 (d, *J* = 10.9 Hz, 1H), 4.51 (bs, 1H); ^{13}C NMR (75 MHz, $CDCl_3$) δ 141.20 (d, *J* = 6.8 Hz), 134.91 (d, *J* = 6.0 Hz), 133.62, 132.99 (d, *J* = 15.0 Hz), 132.59 (d, *J* = 24.0 Hz), 132.41 (d, *J* = 2.3 Hz), 131.70 (d, *J* = 2.3 Hz), 130.88 (d, *J* = 11.3 Hz), 129.46, 128.57 (d, *J* = 10.5 Hz), 127.54 (d, *J* = 12.0 Hz), 126.80, 126.75 (d, *J* = 8.3 Hz), 117.12; ^{31}P NMR (122 MHz, $CDCl_3$) δ 97.03 (q, *J* = 174.5 Hz); HRMS (ESI-MS) $[M-H]^+$: found 241.1032; calculated for $C_{14}H_{15}BOP$: 241.1032.

Chiral HPLC: Chiralpak AS-RH column, Water/Acetonitrile= 65/35, flow rate = 1.0 mL/min, λ = 254 nm.

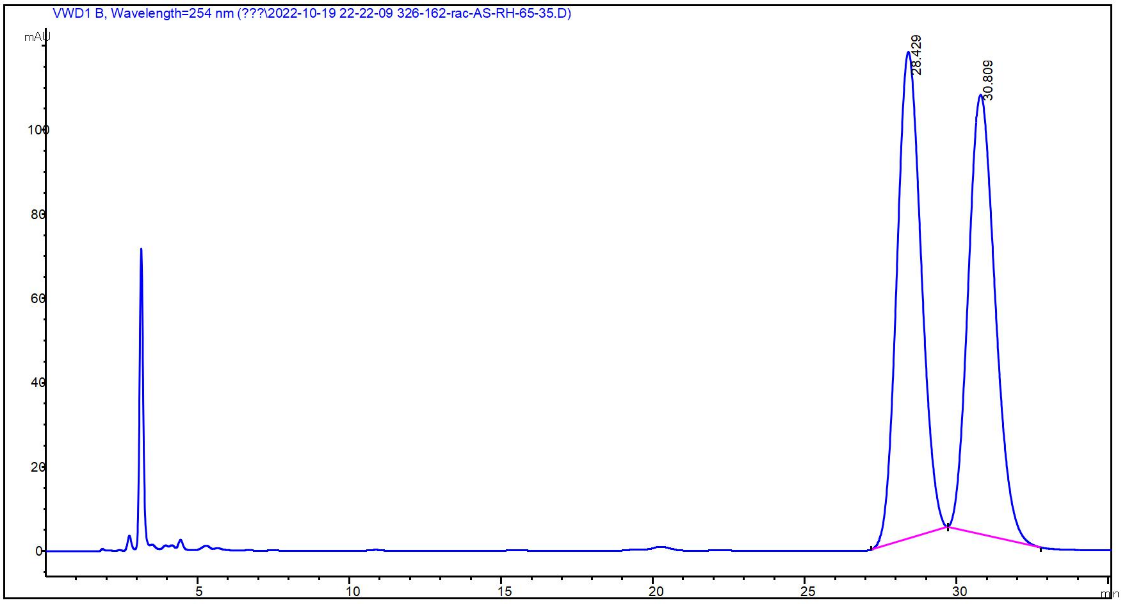

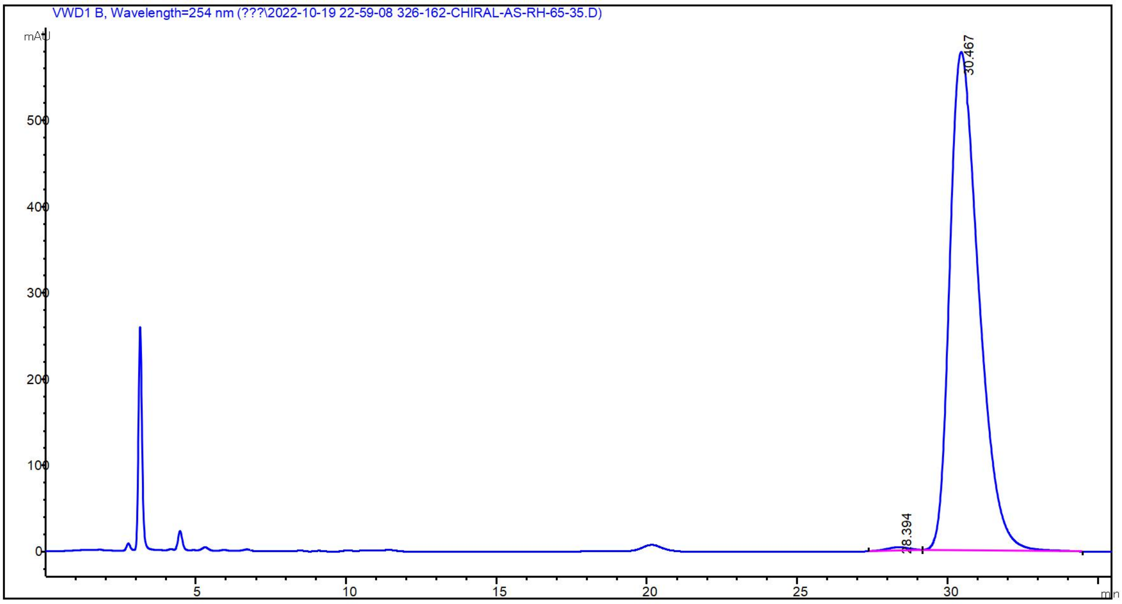

Index	t_R/ (min)	Height / (mv)	Area / (mv.sec)	Area / (%)

1	28.42	115.5	6436.3	50.1
2	30.80	104.3	6403.5	49.9
Total				100.0

Index	t_R/ (min)	Height / (mv)	Area / (mv.sec)	Area / (%)
1	28.39	3.6	177.3	0.4
2	30.46	577.7	38787.2	99.6
Total				100.0

[1,1'-Biphenyl]-2-yl-phenyphosphinous acid-borane 5bj:

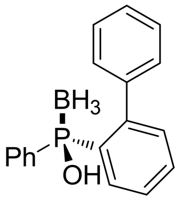

Enantiomeric excess: 95%. 251.2 mg (86% yield), white solid, **m.p.** = 118–120 °C, R_f = 0.30 (PE/EA= 5:1), $[\alpha]_D^{25}$ = -87.3° (c = 1.00 in $CHCl_3$); ^1H NMR (300 MHz, $CDCl_3$) δ 8.13 (dd, J = 12.9, 7.3 Hz, 1H), 7.52 (p, J = 7.2 Hz, 2H), 7.38–7.29 (m, 2H), 7.29–7.16 (m, 6H), 7.11 (t, J = 7.3 Hz, 2H), 6.92 (d, J = 7.5 Hz, 2H), 4.17 (bs, 1H); ^{13}C NMR (75 MHz, $CDCl_3$) δ 146.25 (d, J = 7.5 Hz), 140.17 (d, J = 3.8 Hz), 133.58 (d, J = 77.25 Hz), 133.25 (d, J = 3.8 Hz), 131.61 (d, J = 2.3 Hz), 131.45 (d, J = 7.5 Hz), 131.03 (d, J = 2.3 Hz), 130.68, 130.41 (d, J = 11.3 Hz), 129.63, 128.10 (d, J = 10.5 Hz), 127.57 (d, J = 3.0 Hz), 127.25 (d, J = 3.8 Hz); ^{31}P NMR (122 MHz, $CDCl_3$) δ 96.33 (q, J = 73.2 Hz); HRMS (ESI-MS) $[M-H]^+$: found 291.1190; calculated for $C_{18}H_{17}BOP$: 291.1188.

Chiral HPLC: Chiralpak AS-RH column, Water/Acetonitrile= 65/35, flow rate = 1.0 mL/min, λ = 254 nm.

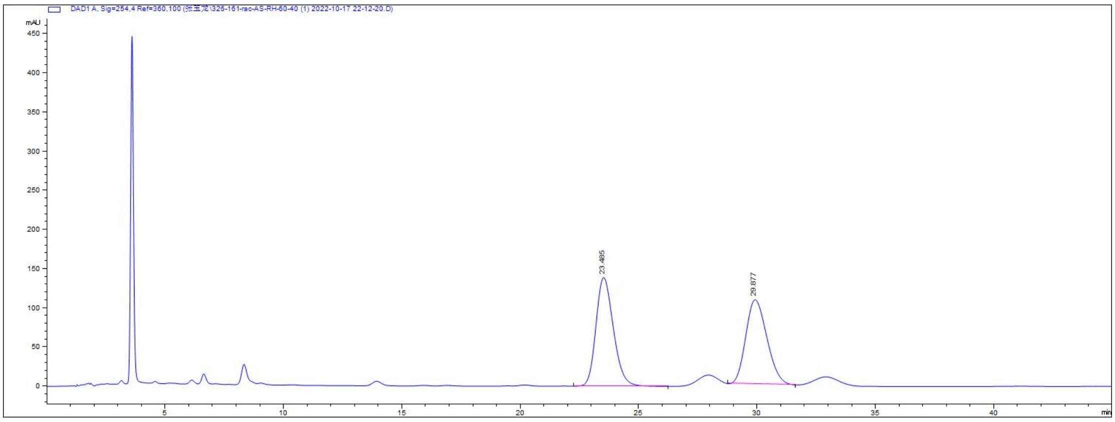

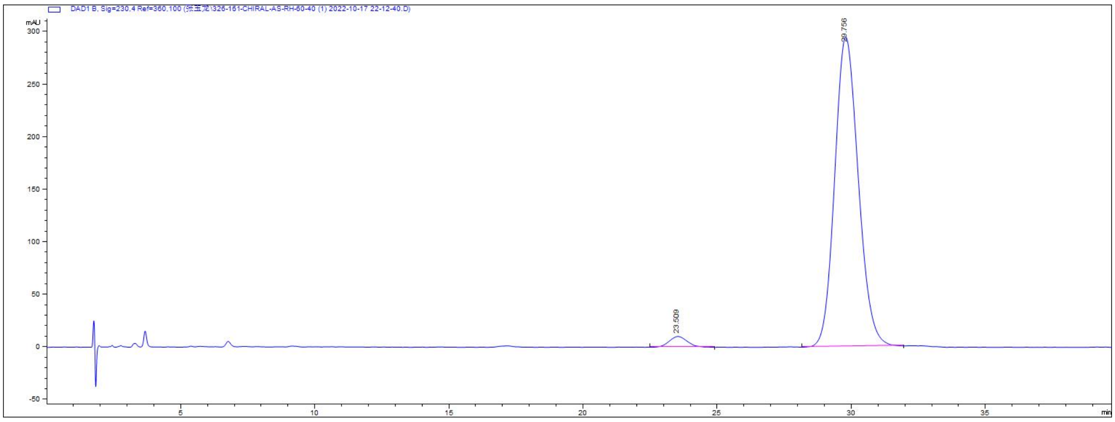

Index	t_R/ (min)	Height / (mv)	Area / (mv.sec)	Area / (%)
1	23.48	138.6	7093.5	51.4
2	29.87	107.7	6694.5	48.6
Total				100.0

Index	t_R/ (min)	Height / (mv)	Area / (mv.sec)	Area / (%)
1	23.50	10.3	493.8	2.6
2	29.76	295.2	18063.5	97.3
Total				100.0

2',6'-Dimethoxy-[1,1'-biphenyl]-2-yl)-phenyphosphinous acid-borane 5bk:

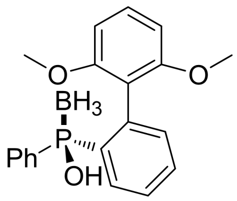

Enantiomeric excess: 97%. 290.8 mg (86% yield), white solid, **m.p.** = 99–101 °C, R_f = 0.35 (PE/EA= 5:1), $[\alpha]_D^{25}$ = -13.2° (c = 1.00 in $CHCl_3$); ^{1}H NMR (300 MHz, $CDCl_3$) δ 8.40 (dd, J = 14.1, 7.2 Hz, 1H), 7.49 (q, J = 7.2 Hz, 2H), 7.04 (dd, J = 26.8, 14.3 Hz, 7H), 6.53 (d, J = 8.3 Hz, 1H), 6.26 (bs, 1H), 6.01 (d, J = 8.3 Hz, 1H), 3.63 (s, 3H), 3.10 (s, 3H), 1.48–0.17 (m, 3H); ^{13}C NMR (75 MHz, $CDCl_3$) δ 157.37, 155.46, 137.27, 135.19 (d, J = 27.75 Hz), 132.57 (d, J = 6.0 Hz), 132.35 (d, J = 2.3 Hz), 130.01, 129.46 (d, J = 2.3 Hz), 129.02 (d, J = 52.5 Hz),127.64, 127.38 (d, J = 11.3 Hz),119.09, 105.43, 105.06, 57.42, 54.90; ^{31}P NMR (122 MHz, $CDCl_3$) δ 91.7 (q, J = 93.9 Hz); HRMS (ESI-MS) $[M-H]^+$: found 337.1078; calculated for $C_{20}H_{18}O_3P$: 337.1072.

Chiral HPLC: Chiralpak AS-RH column, Water/Acetonitrile= 60/40, flow rate = 1.0 mL/min, λ = 254 nm.

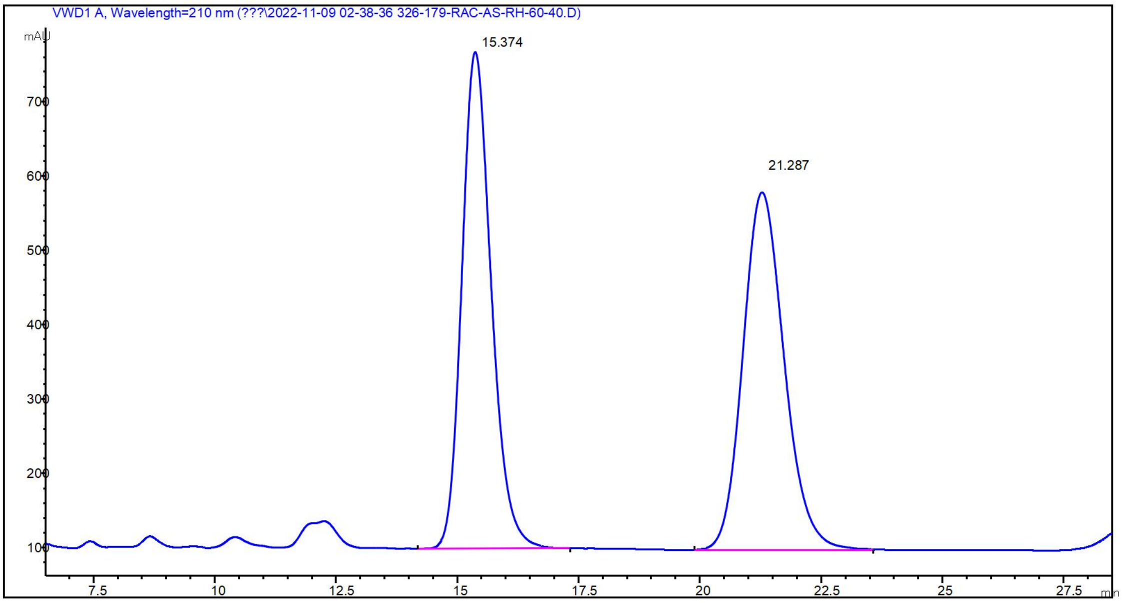

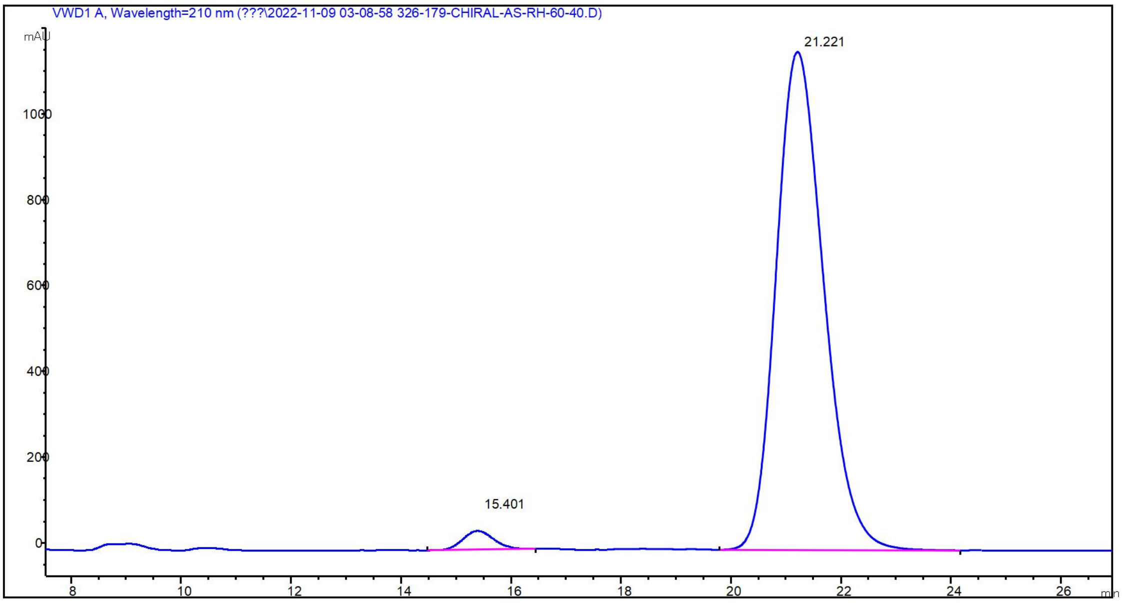

Index	t_R/ (min)	Height / (mv)	Area / (mv.sec)	Area / (%)
1	15.37	667.7	28172.6	49.8
2	21.28	480.2	28359.9	50.2
Total				100.0

Index	t_R/ (min)	Height / (mv)	Area / (mv.sec)	Area / (%)
1	15.40	43.8	1717.7	1.5
2	21.22	1160.3	698.357	98.5
Total				100.0

Naphthalen-1-yl-phenyphosphinous acid-borane 5bl:

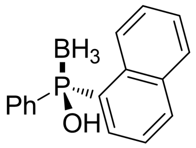

Enantiomeric excess: 99%. 234.1 mg (88% yield), white solid, **m.p.** =126–128 °C, R_f = 0.39 (PE/EA= 5:1), $[\alpha]_D^{25}$ = -43.8° (c = 1.00 in $CHCl_3$); ^{1}H NMR (300 MHz, $CDCl_3$) δ 8.30 (dd, J = 14.6, 6.5 Hz, 1H), 8.16 (d, J = 7.9 Hz, 1H), 8.02 (d, J = 7.6 Hz, 1H), 7.87 (d, J = 7.2 Hz, 1H), 7.78–7.61 (m, 2H), 7.56 (s, 1H), 7.51–7.28 (m, 6H), 1.75–1.21 (m, 3H); ^{13}C NMR (75 MHz, $CDCl_3$) δ 134.27, 133.86 (d, J = 3.8 Hz), 133.71 (d, J = 3.8 Hz), 133.36, 132.50 (d, J = 6.8 Hz), 131.44, 130.74 (d, J = 12.0 Hz), 129.03, 128.92, 128.60 (d, J = 10.5 Hz), 128.12, 126.96, 126.79 (d, J = 6.0 Hz), 126.30, 124.80 (d, J = 13.5 Hz); ^{31}P NMR (122 MHz, $CDCl_3$) δ 95.3 (q, J = 75.6 Hz); HRMS (ESI-MS) $[M-H]^+$: found 265.1035; calculated for $C_{16}H_{15}BOP$: 265.1032.

Chiral HPLC: Chiralpak OJ-RH column, Water/Acetonitrile= 60/40, flow rate = 1.0 mL/min, λ = 254 nm.

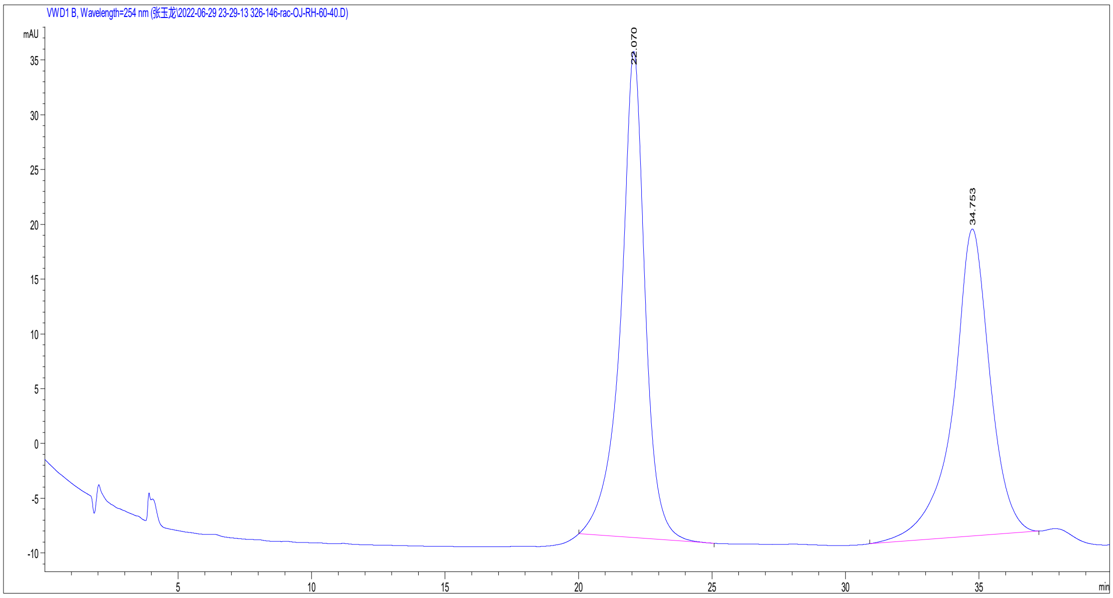

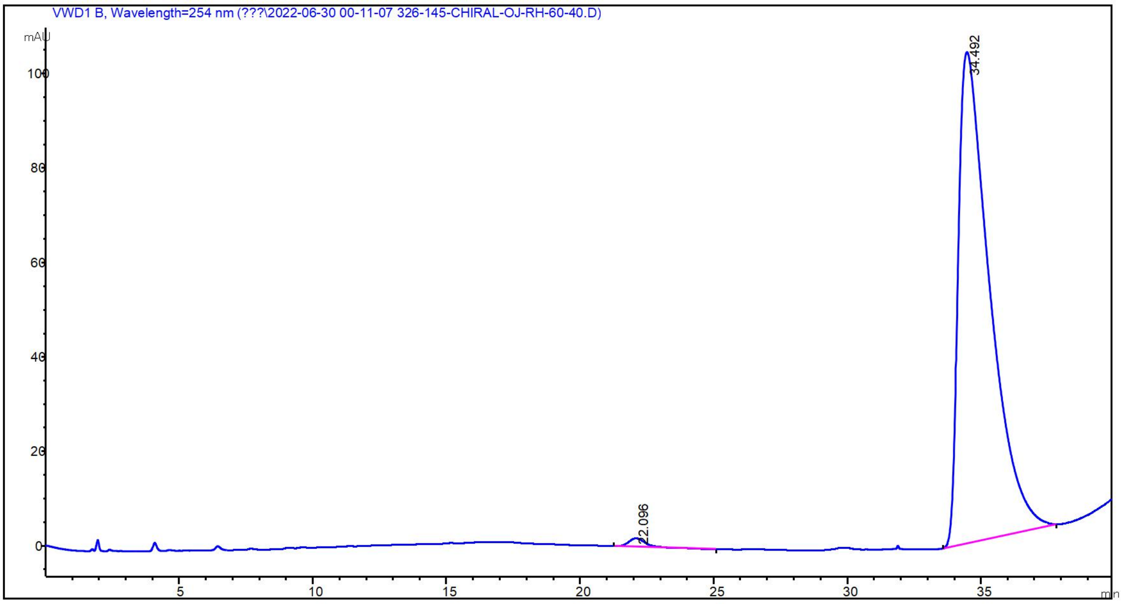

Index	t_R/ (min)	Height / (mv)	Area / (mv.sec)	Area / (%)
1	22.07	44.4	2832.4	51.3
2	34.75	28.0	2682.7	48.7
Total				100.0

Index	t_R/ (min)	Height / (mv)	Area / (mv.sec)	Area / (%)
1	22.09	1.8	77.7	0.5
2	34.49	104	8489.7	99.5
Total				100.0

Naphthalen-2-yl-phenyphosphinous acid-borane 5bm:

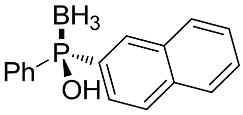

Enantiomeric excess: 99%. 253.1 mg (90% yield), white solid, **m.p.** =123–126 °C, R_f = 0.40 (PE/EA= 5:1), $[\alpha]_D^{25}$ = -38.4° (c = 1.00 in $CHCl_3$); ^{1}H NMR (300 MHz, $CDCl_3$) δ 8.37 (d, J = 13.1 Hz, 1H), 7.87 (q, J = 7.3, 6.7 Hz, 3H), 7.82–7.73 (m, 2H), 7.69 (t, J = 8.8 Hz, 1H), 7.57 (q, J = 6.7 Hz, 2H), 7.52–7.38 (m, 3H), 5.08 (bs, 1H), 1.54–0.64 (m, 3H); ^{13}C NMR (75 MHz, $CDCl_3$) δ 134.66 (d, J = 2.3 Hz), 133.38, 132.75 (d, J = 13.5 Hz), 132.53 (d, J = 4.5 Hz), 132.39, 131.82 (d, J = 2.3 Hz), 130.97 (d, J = 12.0 Hz), 129.98 (d, J = 65.7 Hz), 128.86 (d, J = 15.8 Hz), 128.61, 128.50, 128.04 (d, J = 24.8 Hz), 126.96, 125.85 (d, J = 10.5 Hz); ^{31}P NMR (122 MHz, $CDCl_3$) δ 95.02 (q, J = 86.6 Hz); HRMS (ESI-MS) $[M-H]^+$: found 265.1035; calculated for $C_{16}H_{15}BOP$: 265.1032.

Chiral HPLC: Chiralpak OJ-RH column, Water/Acetonitrile= 65/35, flow rate = 1.0 mL/min, λ = 254 nm.

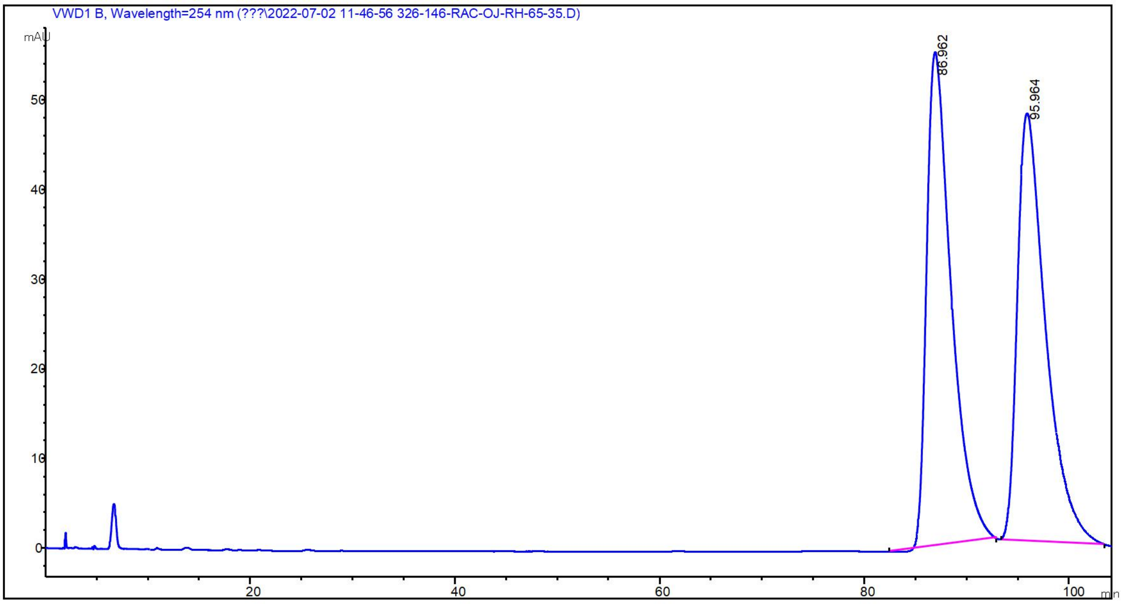

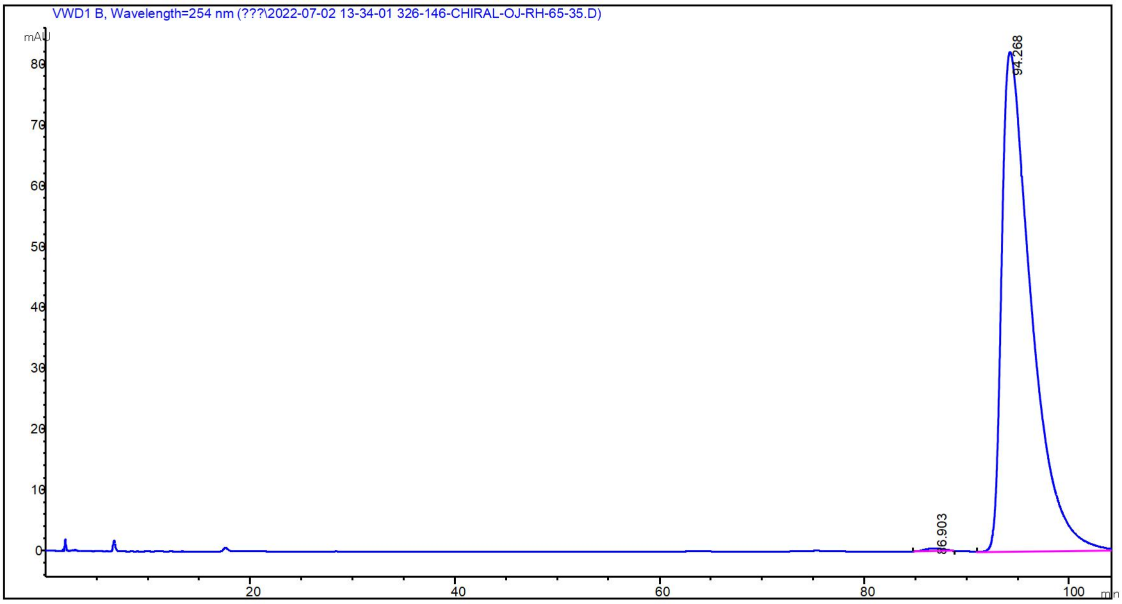

Index	t_R/ (min)	Height / (mv)	Area / (mv.sec)	Area / (%)
1	86.96	55.0	9168.2	50.0
2	95.96	47.6	9163.7	50.0
Total				100.0

Index	t_R/ (min)	Height / (mv)	Area / (mv.sec)	Area / (%)
1	86.90	0.5	68.4	0.4
2	94.26	82.1	16617.5	99.6
Total				100.0

Dibenzo[b,d]furan-4-yl-phenyphosphinous acid-borane 5bn:

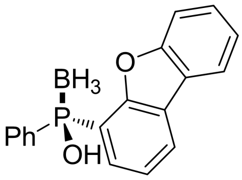

Enantiomeric excess: 99%. 284.6 mg (93% yield), white solid, **m.p.** =91–96 °C, R_f = 0.32 (PE/EA= 5:1), $[\alpha]_D^{25}$ = -12.4° (c = 1.00 in $CHCl_3$); ^1H NMR (300 MHz, $CDCl_3$) δ 8.10 (d, J = 7.7 Hz, 1H), 7.99–7.77 (m, 4H), 7.53–7.43 (m, 6H), 7.35 (t, J = 7.3 Hz, 1H), 5.42 (bs, 1H); ^{13}C NMR (75 MHz, $CDCl_3$) δ 156.08 (d, J = 4.5 Hz), 132.42 (d, J = 66.0 Hz), 131.89 (d, J = 2.3 Hz), 130.92 (d, J = 6.0 Hz), 130.64, 128.57 (d, J = 11.25 Hz), 127.81, 124.94 (d, J = 2.3 Hz), 124.83 (d, J = 4.5 Hz), 123.36, 123.05, 122.92, 120.81, 116.39 (d, J = 60.0 Hz), 112.05; ^{31}P NMR (122 MHz, $CDCl_3$) δ 92.50 (q, J = 76.9 Hz); HRMS (ESI-MS) $[M-H]^+$: found 305.0983; calculated for $C_{18}H_{15}BOP$: 305.0981.

Chiral HPLC: Chiralpak OJ-RH column, Water/Acetonitrile= 65/35, flow rate = 1.0 mL/min, λ = 254 nm.

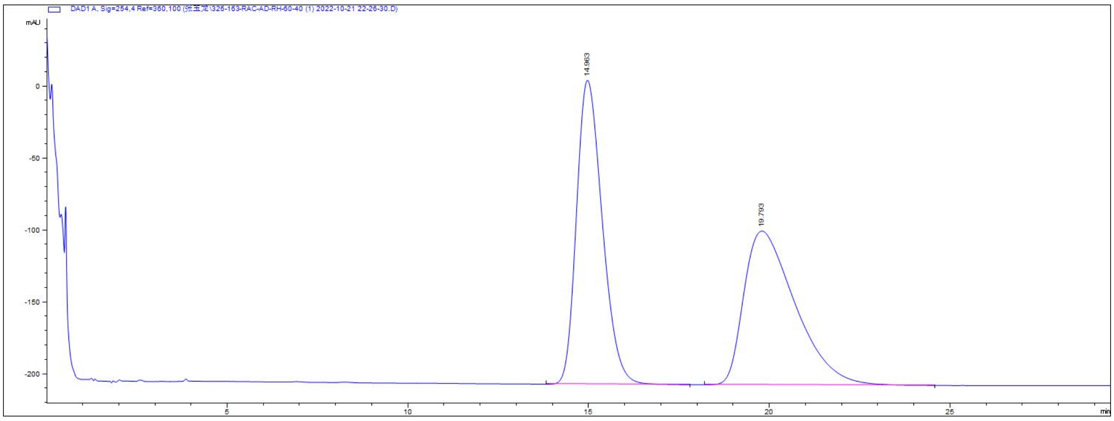

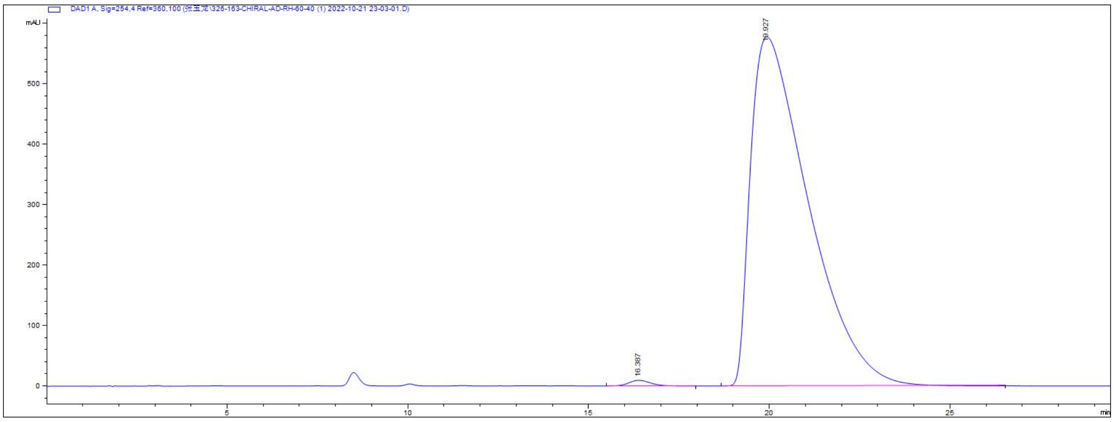

Index	t_R/ (min)	Height / (mv)	Area / (mv.sec)	Area / (%)
1	14.96	211.6	10518.4	49.8
2	19.79	107.2	10574.7	50.2
Total				100.0

Index	t_R/ (min)	Height / (mv)	Area / (mv.sec)	Area / (%)
1	16.38	9.3	393.3	0.6

2	19.92	578.9	64649.1	99.4
Total				100.0

Dibenzo[b,d]thiophen-4-yl-phenyphosphinous acid-borane 5bo (CCDC 2251985):

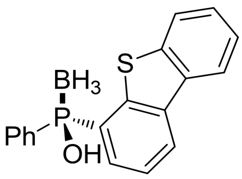

Enantiomeric excess: 99%. 312.3 mg (93% yield), white solid, **m.p.** =99–105 °C, R_f = 0.42 (PE/EA= 5:1), $[\alpha]_D^{25}$ = -5.4 ° (c = 1.00 in $CHCl_3$); ^{1}H NMR (300 MHz, $CDCl_3$) δ 8.18 (d, *J* = 44.3 Hz, 3H), 7.80 (s, 3H), 7.44 (s, 6H), 5.16 (bs, 1H), 1.24 (s, 3H); ^{13}C NMR (75 MHz, $CDCl_3$) δ 141.08 (d, *J* = 4.5 Hz),139.86, 136.72 (d, *J* = 4.5 Hz), 134.12, 132.06, 131.56 (d, *J* = 15.8 Hz),131.16 (d, *J* = 12.0 Hz),128.65 (d, *J* = 10.5 Hz), 127.29, 126.67 (d, *J* = 64.5 Hz), 124.86 (d, *J* = 34.5 Hz), 124.33 (d, *J* = 12.0 Hz), 122.53, 121.65; ^{31}P NMR (122 MHz, $CDCl_3$) δ 96.95 (q, *J* = 79.3 Hz); HRMS (ESI-MS) $[M-H]^+$: found 321.0762; calculated for $C_{18}H_{15}BSP$: 321.0753.

Chiral HPLC: Chiralpak AS-RH column, Water/Acetonitrile= 60/40, flow rate = 1.0 mL/min, λ = 254 nm.

***RAC*-5bo**

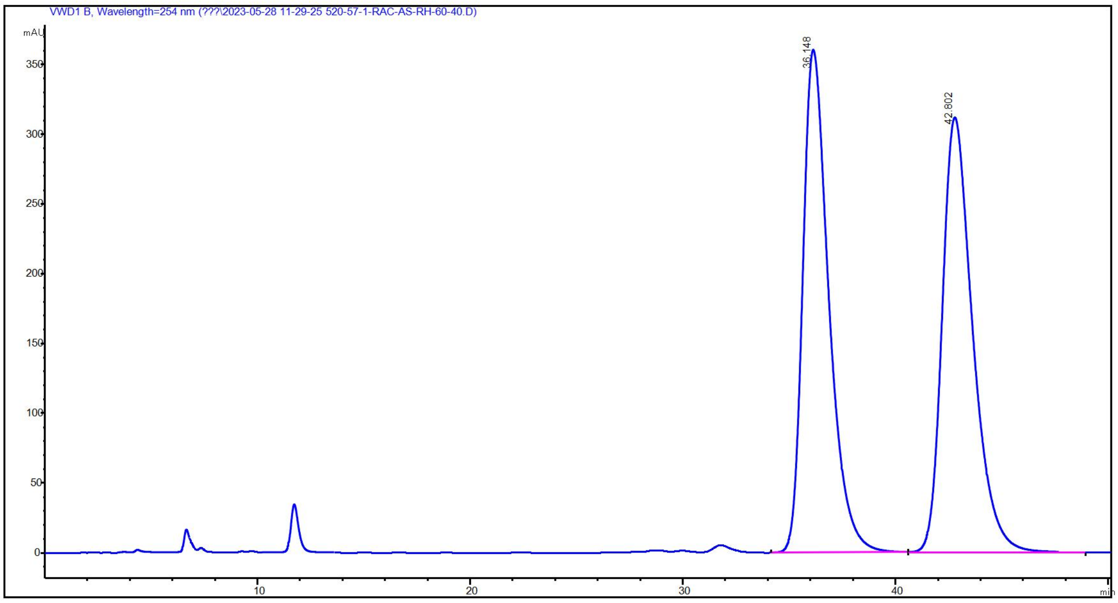

(R_p)-5bo

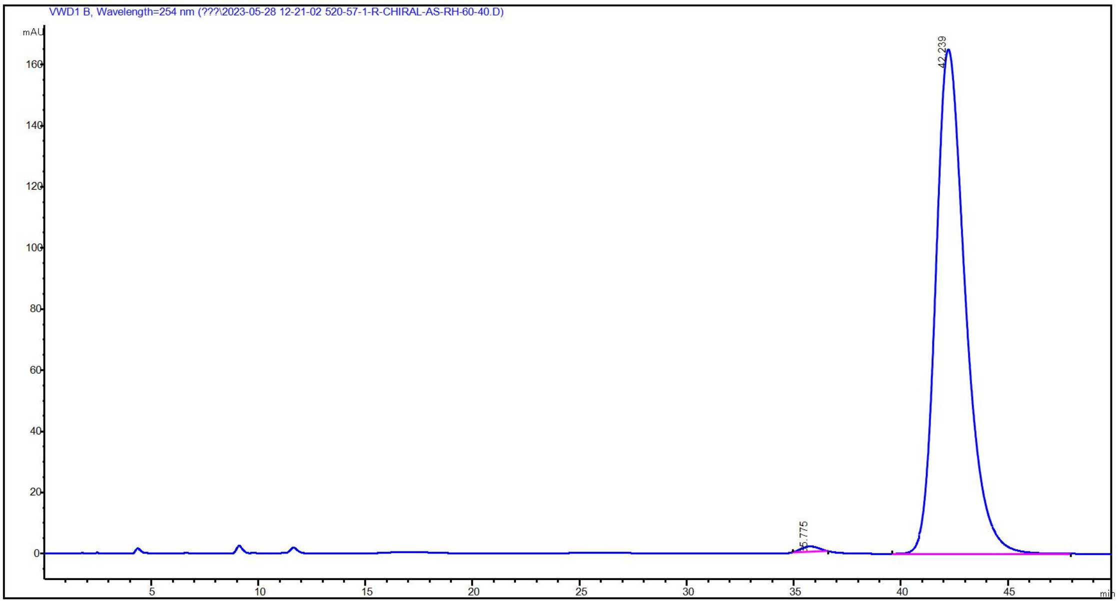

(S_p)-5bo

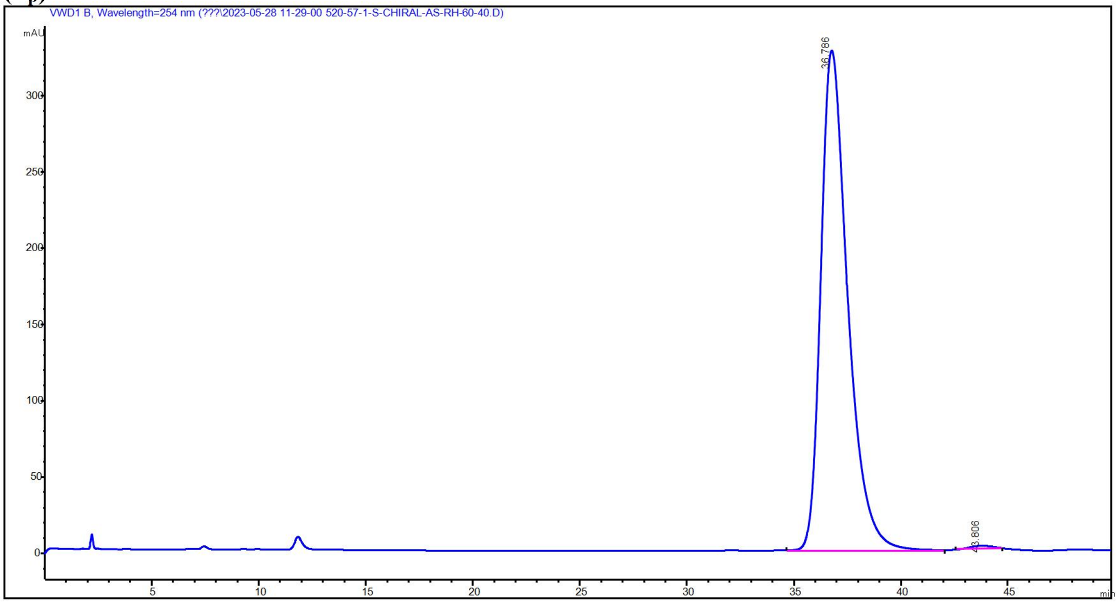

***RAC*-5bo**

Index	t_R / (min)	Height / (mv)	Area /	Area / (%)
1	36.14	360.3	30564.6	49.8
2	42.80	311.5	30861.0	50.2
Total				100.0

(R_p)-5bo

Index	t_R / (min)	Height / (mv)	Area /	Area / (%)
1	35.77	1.8	106.6	0.6
2	42.23	164.9	15900.5	99.4
Total				100.0

(S_p)-5bo

Index	t_R / (min)	Height / (mv)	Area /	Area / (%)
1	36.78	327.4	28581.6	99.5
2	43.80	2.1	147.3	0.5
Total				100.0

***o*-Methoxyphenyl-methylphosphinous acid-borane 5bp:**

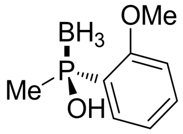

Enantiomeric excess: 97%. 165.6 mg (90% yield), white solid, **m.p.** =82–84 °C, R_f = 0.62 (PE/EA= 5:1), $[\alpha]_D^{25}$ = -18.8 ° (c = 1.00 in $CHCl_3$); ^1H NMR (300 MHz, $CDCl_3$) δ 7.82 (s, 1H), 7.53 (s, 1H), 7.20–6.79 (m, 2H), 5.51 (bs, 1H), 3.94 (s, 3H), 1.81 (s, 3H), 0.83 (dd, J = 175.3, 97.1 Hz, 3H); ^{13}C NMR (75 MHz, $CDCl_3$) δ 160.55, 133.92, 133.40 (d, J = 15.8 Hz), 121.39 (d, J = 12.0 Hz), 110.90 (d, J = 3.8 Hz), 55.93, 17.05 (d, J = 41.3 Hz); ^{31}P NMR (122 MHz, $CDCl_3$) δ 100.67 (q, J = 215.9 Hz); HRMS (ESI-MS) $[M-H]^+$: found 183.0830; calculated for $C_8H_{13}BO_2P$: 183.0824.

Chiral HPLC: Chiralpak AS-RH column, Water/Acetonitrile= 70/30, flow rate = 1.0 mL/min, λ = 254 nm.

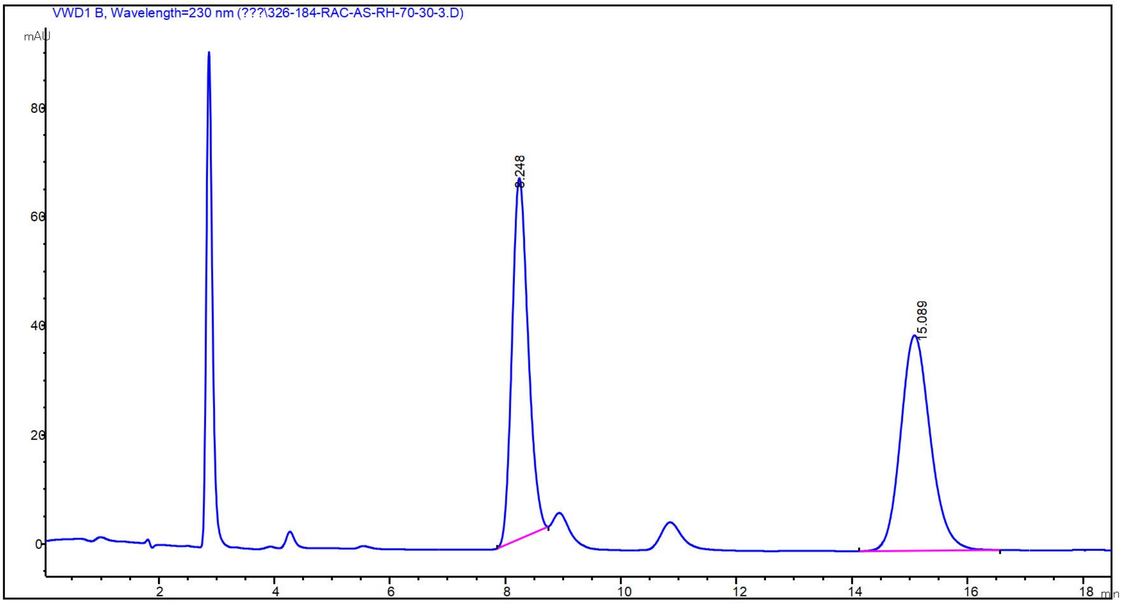

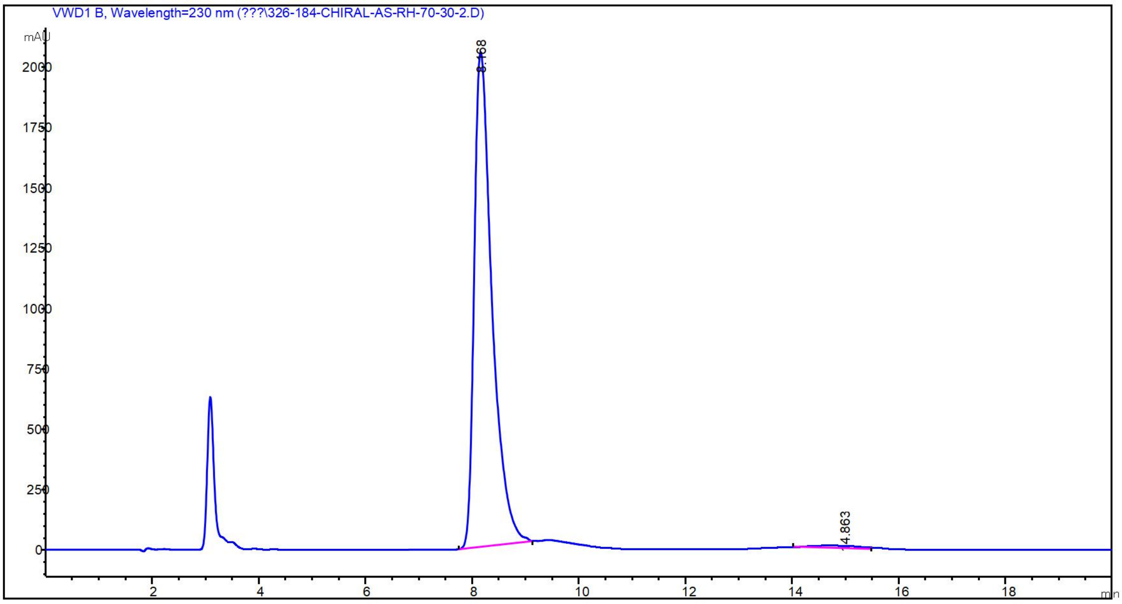

Index	t_R/ (min)	Height / (mv)	Area / (mv.sec)	Area / (%)
1	8.24	66.2	1283.2	47.5
2	15.08	39.5	1415.6	52.5
Total				100.0

Index	t_R/ (min)	Height / (mv)	Area / (mv.sec)	Area / (%)
1	8.16	2049.4	47618.0	98.5
2	14.86	11.5	736.5	1.5
Total				100.0

***m*-Methoxyphenyl-methylphosphinous acid-borane 5bq:**

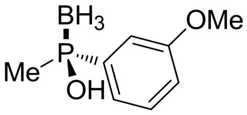

Enantiomeric excess: 82%. 158.3 mg (86% yield), colorless oil, R_f = 0.67 (PE/EA= 5:1), $[\alpha]_D^{25}$ = -28.4 ° (c = 1.00 in $CHCl_3$); ^1H NMR (300 MHz, $CDCl_3$) δ 7.42–7.27 (m, 3H), 7.02 (td, *J* = 32.7, 28.0, 6.3 Hz, 2H), 3.82 (s, 4H), 1.67 (d, *J* = 9.3 Hz, 3H); ^{13}C NMR (75 MHz, $CDCl_3$) δ 159.33 (d, *J* = 12.4 Hz), 135.23 (d, *J* = 58.6 Hz), 130.03 (d, *J* = 11.8 Hz), 122.89 (d, *J* = 6.2 Hz), 122.17 (d, *J* = 11.9 Hz), 117.62, 114.96 (d, *J* = 17.1 Hz), 55.45 , 17.65 (d, *J* = 45.9 Hz); ^{31}P NMR (122 MHz, $CDCl_3$) δ 102.45–98.01 (m); HRMS (ESI-MS) $[M-H]^+$: found 183.0826; calculated for $C_8H_{13}BO_2P$: 183.0824.

Chiral HPLC: Chiralpak AS-RH column, Water/Acetonitrile= 70/30, flow rate = 1.0 mL/min, λ = 254 nm.

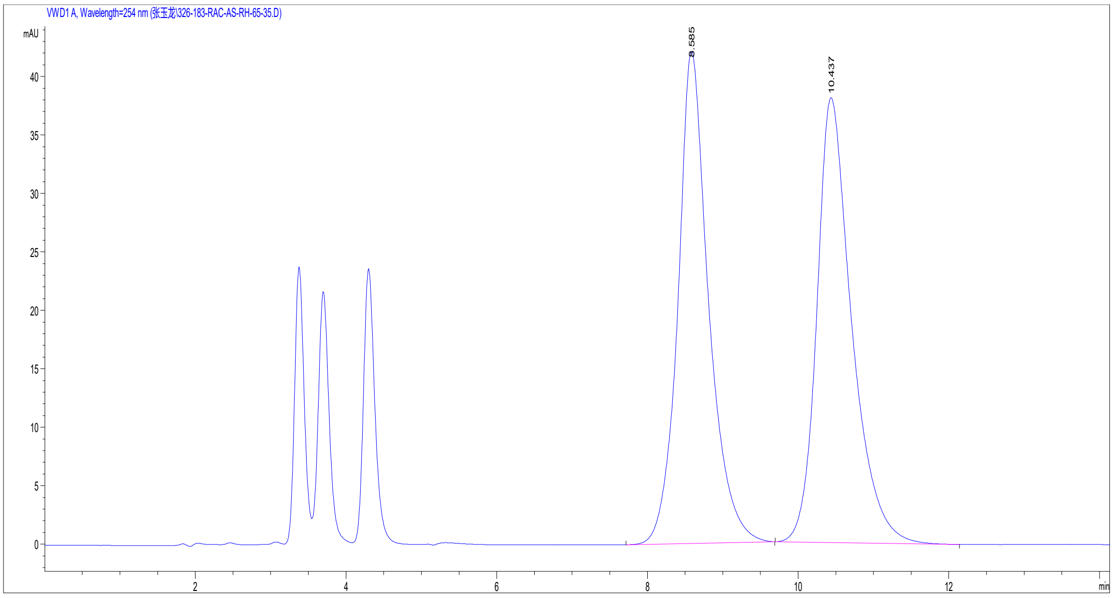

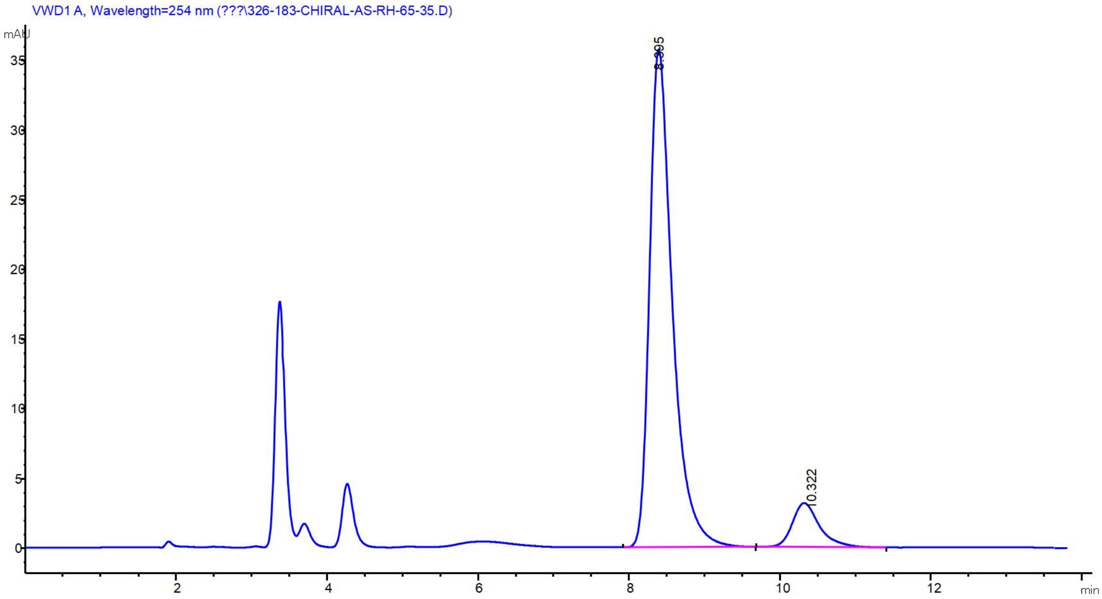

Index	t_R/ (min)	Height / (mv)	Area / (mv.sec)	Area / (%)
1	8.58	42.1	1188.3	49.4
2	10.43	38	1220.4	50.6
Total				100.0

Index	t_R/ (min)	Height / (mv)	Area / (mv.sec)	Area / (%)
1	8.39	35.7	752.5	90.8
2	10.32	3.2	78	9.2
Total				100.0

***p*-Methoxyphenyl-methylphosphinous acid-borane 5br:**

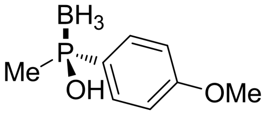

Enantiomeric excess: 65%. 165.6 mg (90% yield), colorless oil, R_f = 0.62 (PE/EA= 5:1), $[\alpha]_D^{25}$ = -3.8° (c = 1.00 in $CHCl_3$); ^{1}H NMR (300 MHz, $CDCl_3$) δ 7.70 (t, *J* = 9.3 Hz, 2H), 6.96 (d, *J* = 7.8 Hz, 2H), 5.04 (bs, 1H), 3.83 (s, 3H), 1.69 (d, *J* = 9.4 Hz, 3H), 0.83 (dd, *J* = 173.1, 84.9 Hz, 3H); ^{13}C NMR (75 MHz, $CDCl_3$) δ 162.30 (d, *J* = 1.5 Hz), 132.05 (d, *J* = 12.8 Hz),114.30 (d, *J* = 12.0 Hz), 55.52, 17.50 (d, *J* = 45.0 Hz); ^{31}P NMR (122 MHz, $CDCl_3$) δ 96.59 (q, *J* = 209.8 Hz); HRMS (ESI-MS) $[M-H]^+$: found 183.0831; calculated for $C_8H_{13}BO_2P$: 183.0824.

Chiral HPLC: Chiralpak AS-RH column, Water/Acetonitrile= 70/30, flow rate = 1.0 mL/min, λ = 254 nm.

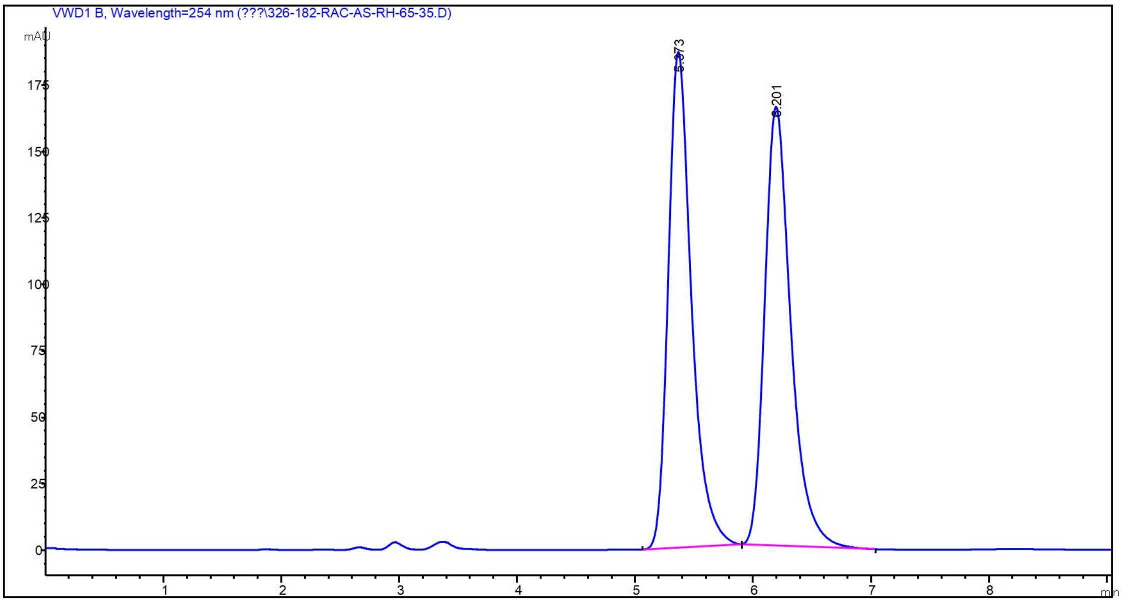

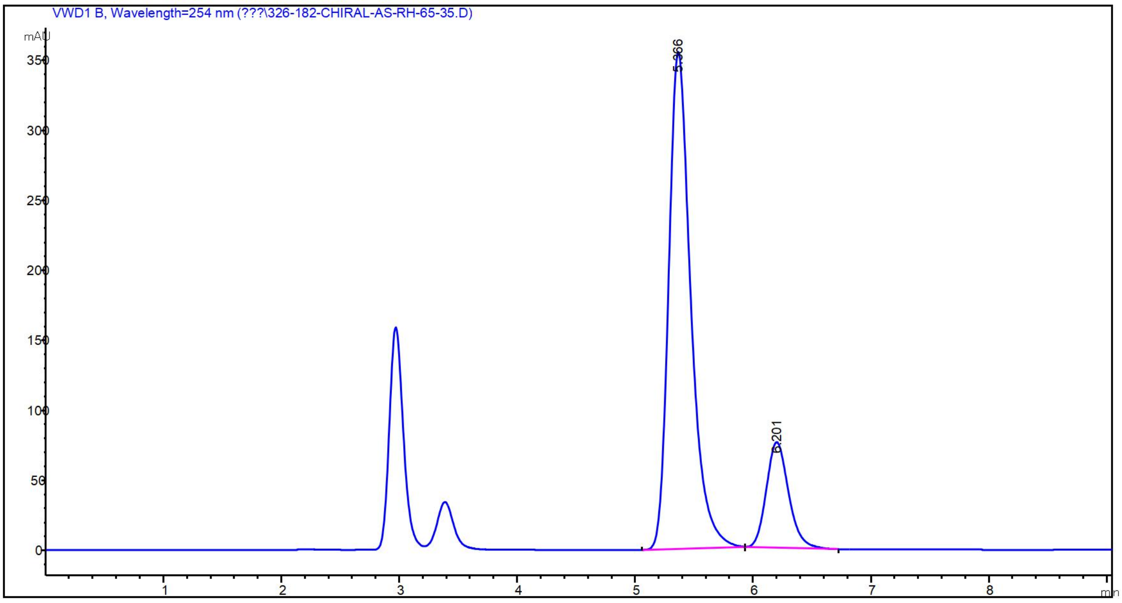

Index	t_R/ (min)	Height / (mv)	Area / (mv.sec)	Area / (%)
1	5.37	186.4	2548.2	50.0
2	6.20	165	2540.0	50.0
Total				100.0

Index	t_R/ (min)	Height / (mv)	Area / (mv.sec)	Area / (%)
1	5.36	355	4620.0	82.4
2	6.20	75.4	987.8	17.6
Total				100.0

General procedure for synthesis of phosphinousacid-borane 5ca-cc

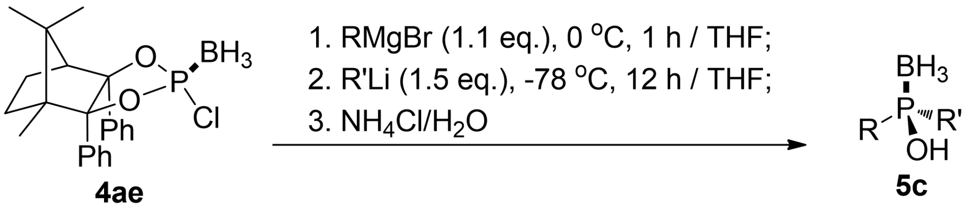

A Schlenk tube was dried under vacuum. After cooling, the tube was placed under argon atmosphere. Under an argon atmosphere, to a stirred solution of CAMDOL (**1e**, 1 equiv.) in dry THF was added triethylamine (2.5 equiv.). To the mixture at 0 °C was added trichlorophosphine (1.5 equiv.) and the mixture remained at 0 °C. After ^{31}P NMR analysis of a small aliquot showed complete consumption, $BH_3{\cdot}SMe_2$ (2.0 equiv.) was added dropwise and stirred to 0 °C at until ^{31}P NMR showed complete consumption of intermediate material. Grignard reagent (1.1 equiv.) was then added dropwiseand the resulting solution was allowed to stir until ^{31}P NMR showed complete consumption of **4ae.** To the resulting mixture was added saturated aqueous NH_4Cl solution (20 mL) and EtOAc (40 mL). The layers were separated, and the aqueous layer was washed with EtOAc (2 x 20 mL). The combined organic layers were washed with brine (20 mL), dried over anhydrous Na_2SO_4, concentrated and dried *in vacuo*.

The crude diphenyl-camdol-dioxaphosphine phosphine borane (1 equiv.) was dissolved in THF (15 mL) and charged into a 50 mL flask under argon atmosphere, which was then added dropwise to the flask containing the organolithium reagent. The resulting mixture was stirred for 3 h while being kept at –78 °C. After ^{31}P NMR analysis of a small aliquot showed complete consumption of starting material, the reaction was warmed to room temperature. To the resulting mixture was added saturated aqueous NH_4Cl solution (20 mL) and EtOAc (40 mL). The layers were separated, and the aqueous layer was washed with EtOAc (2 x 20 mL). The combined organic layers were washed with brine (20 mL), dried over anhydrous Na_2SO_4, filtered and concentrated. The residue was purified by silica gel chromatography to afford the desired product.

[1,1'-Biphenyl]-2-yl -vinylphosphinous acid-borane 5ca:

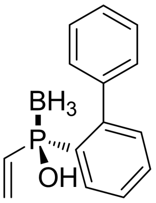

Enantiomeric excess: 96%. 40.8 mg (83% yield), white solid, **m.p.** = 99–103 °C, R_f = 0.62 (PE/EA= 5:1), $[\alpha]_D^{25}$ = -28.3 ° (c = 1.00 in $CHCl_3$); ^1H NMR (300 MHz, $CDCl_3$) δ 8.02 (dd, J = 13.5, 7.6 Hz, 1H), 7.51 (dt, J = 14.9, 7.4 Hz, 2H), 7.38 (s, 5H), 7.31 (dd, J = 7.2, 3.5 Hz, 1H), 5.95–5.82 (m, 2H), 5.82–5.64 (m, 1H); ^{13}C NMR (75 MHz, $CDCl_3$) δ 145.91 (d, J = 6.0 Hz),140.79 (d, J = 3.75 Hz), 132.89 (d, J = 17.3 Hz), 132.13 (d, J = 6.8 Hz), 131.59 (d, J = 2.3 Hz), 131.52, 131.22 (d, J = 6.8 Hz), 130.85, 130.72, 130.03, 129.95, 129.33, 128.57, 128.18, 127.87, 127.39 (d, J = 11.3 Hz); ^{31}P NMR (122 MHz, $CDCl_3$) δ 95.72 (q, J = 190.3 Hz); HRMS (ESI-MS) $[M-H]^+$: found 241.1032; calculated for $C_{14}H_{15}BOP$: 241.1032.

Chiral HPLC: Chiralpak OJ-RH column, Water/Acetonitrile= 60/40, flow rate = 1.0 mL/min, λ = 230 nm.

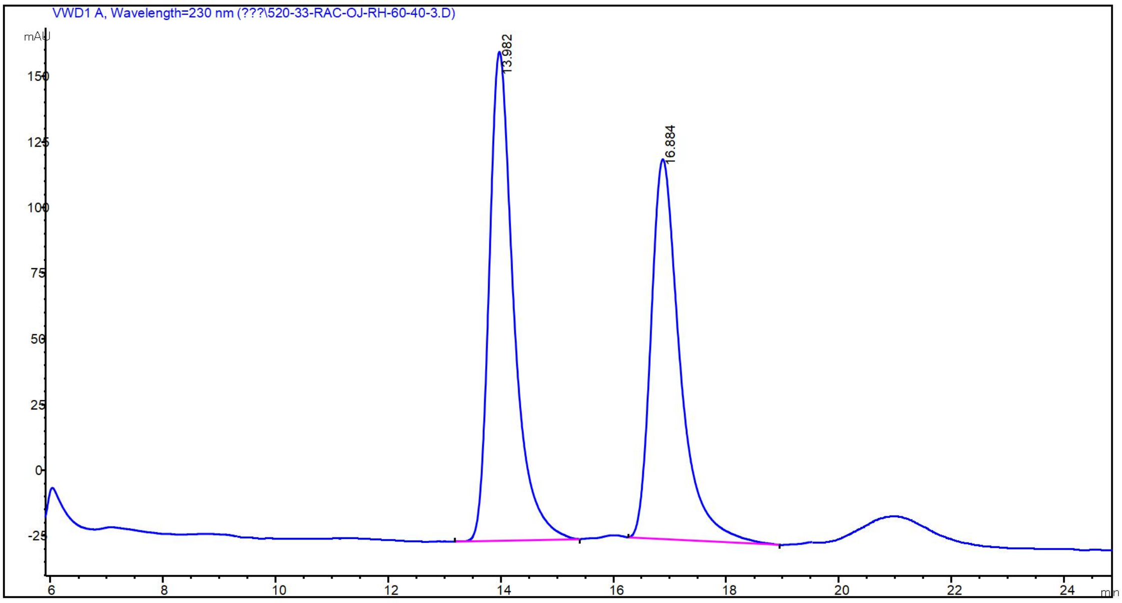

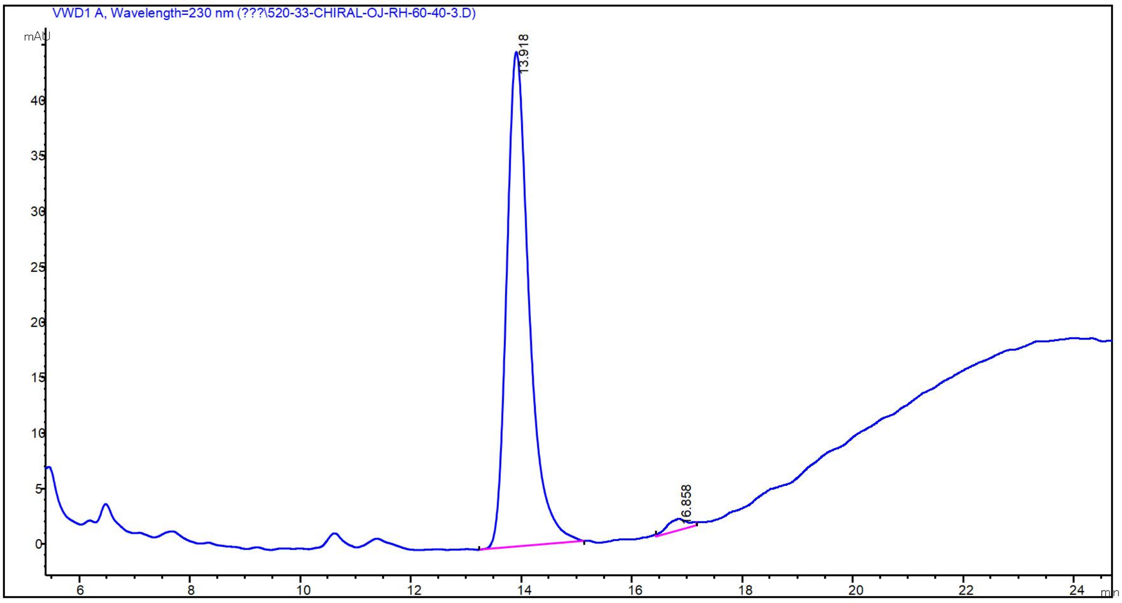

Index	t_R/ (min)	Height / (mv)	Area / (mv.sec)	Area / (%)
1	13.98	186.1	5639.9	51.9
2	16.88	144.6	5180.8	48.1
Total				100.0

Index	t_R/ (min)	Height / (mv)	Area / (mv.sec)	Area / (%)
1	13.91	44.6	1262.2	97.8
2	16.85	1.0	28.5	2.2
Total				100.0

Naphthalen-1-yl-cyclohexylphosphinous acid-borane 5cb:

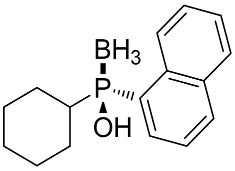

Enantiomeric excess: 97%. 54.4 mg (90% yield), white solid, **m.p.** = 110–113 °C, R_f = 0.50 (PE/EA= 5:1), $[\alpha]_D^{25}$ = -78.4 ° (c = 1.00 in $CHCl_3$); ^{1}H NMR (300 MHz, $CDCl_3$) δ 8.49 (d, J = 8.0 Hz, 1H), 8.21–8.02 (m, 2H), 7.83–7.74 (m, 1H), 7.63–7.51 (m, 1H), 7.33–7.20 (m, 1H), 6.82 (d, J = 7.4 Hz, 1H), 2.39–2.16 (m, 1H), 1.90–0.97 (m, 13H); ^{13}C NMR (75 MHz, $CDCl_3$) δ 151.20, 132.85 (d, J = 2.7 Hz), 129.29, 127.84 (d, J = 13.8 Hz), 126.41 (d, J = 8.2 Hz), 126.20 (d, J = 4.7 Hz), 125.60 (d, J = 43.0 Hz), 124.73 (d, J = 13.0 Hz), 121.18 (d, J = 59.0 Hz), 108.83 , 38.45 (d, J = 42.2 Hz), 26.41 , 26.24 (d, J = 1.9 Hz), 25.76 (d, J = 1.3 Hz), 25.68 (d, J = 3.0 Hz), 25.44 (d, J = 1.7 Hz);^{31}P NMR (122 MHz,$CDCl_3$) δ 110.96 (q, J = 81.5 Hz); HRMS (ESI-MS) $[M\text{-}H]^+$: found271.1506; calculated for $C_{16}H_{21}BOP$: 271.1501.

Chiral HPLC: Chiralpak AS-RH column, Water/Acetonitrile= 50/50, flow rate = 1.0 mL/min, λ = 254 nm.

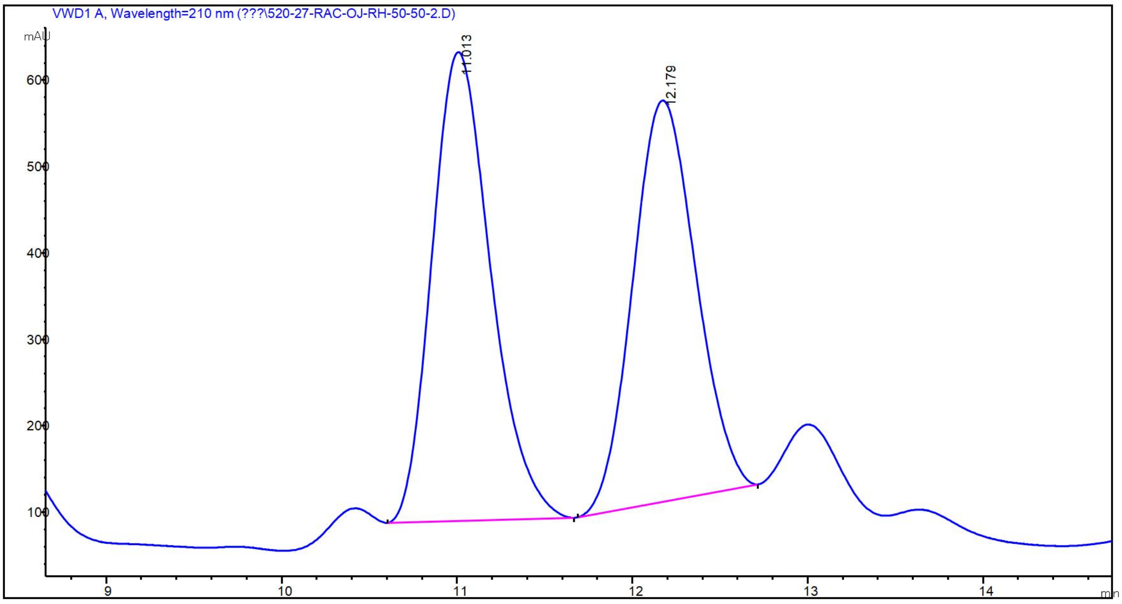

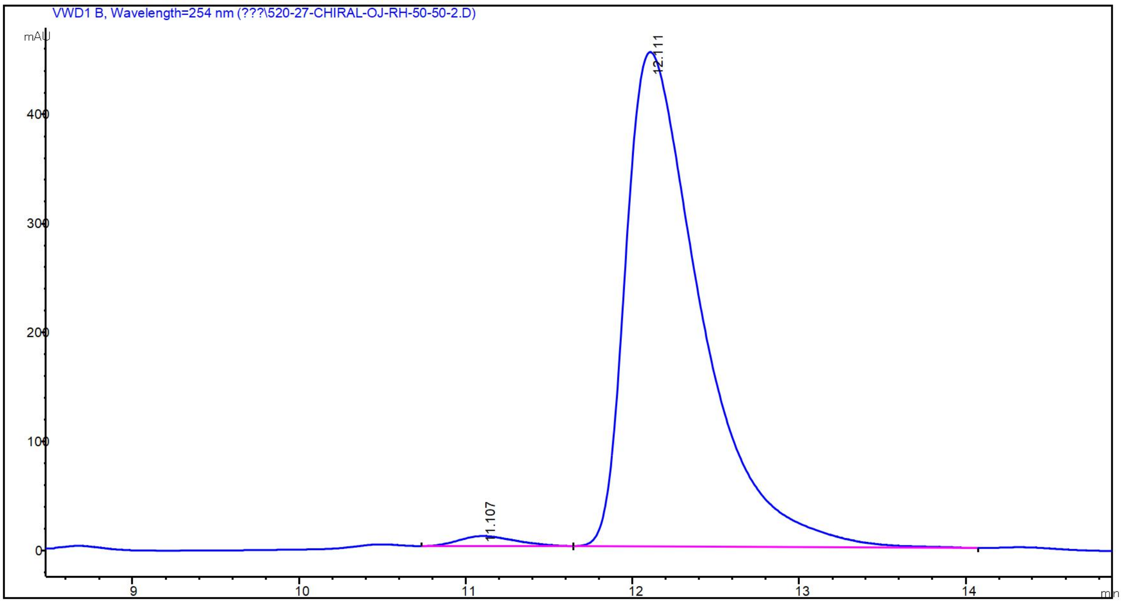

Index	t_R/ (min)	Height / (mv)	Area / (mv.sec)	Area / (%)
1	11.01	542.6	12660.2	51.0
2	12.17	646.4	11579.4	49.0
Total				100.0

Index	t_R/ (min)	Height / (mv)	Area / (mv.sec)	Area / (%)
1	11.10	9.1	212.7	1.5
2	12.11	453.5	14186.2	98.5
Total				100.0

2-Vinylphenyl-4-fluorophenylphosphinous acid-borane 5cc:

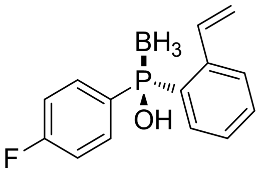

Enantiomeric excess: 98%. 46.2 mg (89% yield), white solid, **m.p.** = 89–93 °C, R_f = 0.33 (PE/EA= 5:1), $[\alpha]_D^{25}$ = -26.3 ° (c = 1.00 in $CHCl_3$); ^1H NMR (300 MHz, $CDCl_3$) δ 7.98 (dd, J = 14.1, 7.7 Hz, 1H), 7.69–7.58 (m, 2H), 7.54 (t, J = 7.5 Hz, 1H), 7.47–7.36 (m, 1H), 7.13–7.07 (m, 2H), 6.98 (dd, J = 17.1, 10.9 Hz, 1H), 5.54 (d, J = 17.1 Hz, 1H), 5.14 (d, J = 11.6 Hz, 1H); ^{13}C NMR (75 MHz, $CDCl_3$) δ 166.56 (d, J = 2.3 Hz), 163.20 (d, J = 3.0 Hz), 141.17 (d, J = 7.5 Hz), 134.75 (d, J = 6.8 Hz), 133.50 (q, J = 8.3, J = 9.0 Hz), 132.81 (d, J = 14.3 Hz), 127.62 (d, J = 11.3 Hz), 126.86 (d, J = 7.5 Hz), 117.33, 115.97 (q, J = 12.0 Hz, J = 21.0 Hz); ^{31}P NMR (122 MHz, $CDCl_3$) δ 96.45 (q, J = 92.7 Hz); ^{19}F NMR (282 MHz, $CDCl_3$) δ -106.92 (dqt, J = 8.3, 5.5, 2.2 Hz); HRMS (ESI-MS) $[M-H]^+$: found259.0940;calculated for $C_{14}H_{14}BFOP$: 259.0938.

Chiral HPLC: Chiralpak AS-RH column, Water/Acetonitrile= 60/40, flow rate = 1.0 mL/min, λ = 254 nm.

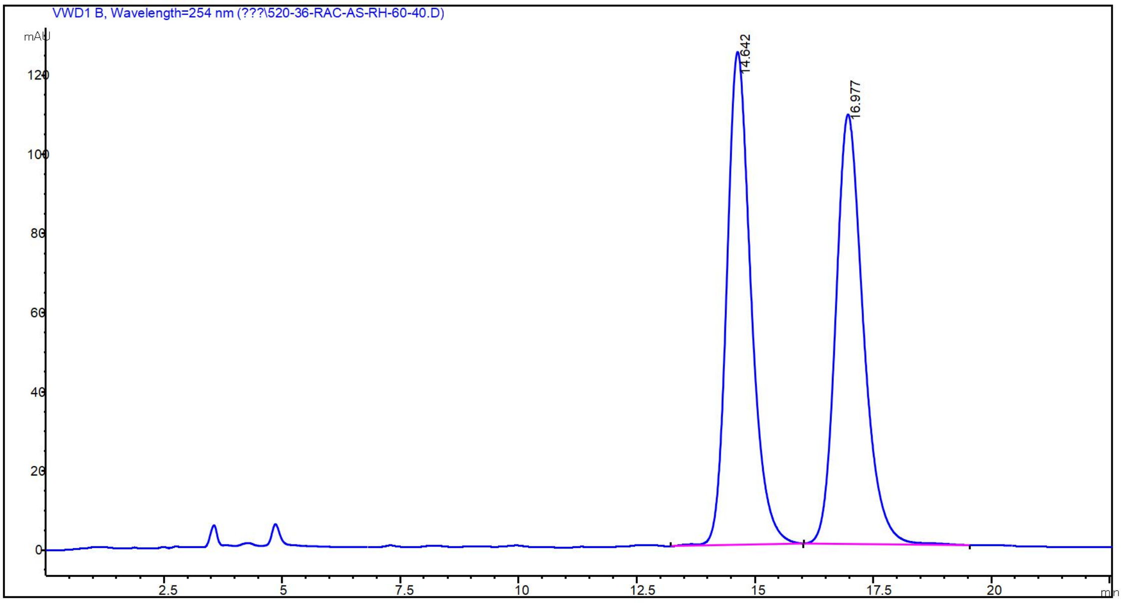

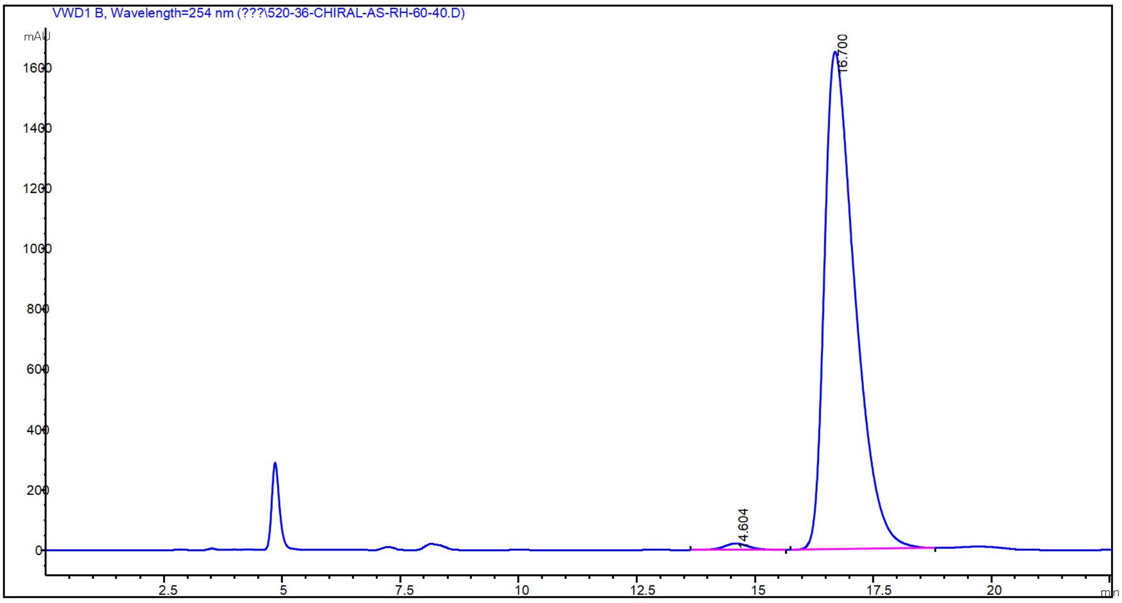

Index	t_R/ (min)	Height / (mv)	Area / (mv.sec)	Area / (%)
1	14.64	124.5	4406.3	50.1
2	16.97	108.5	4391.8	49.9
Total				100.0

Index	t_R/ (min)	Height / (mv)	Area / (mv.sec)	Area / (%)
1	14.60	21.6	802.9	1.0
2	16.70	1649.3	73926.2	99.0
Total				100.0

General procedure for synthesis of P(III)-ligand PAMP

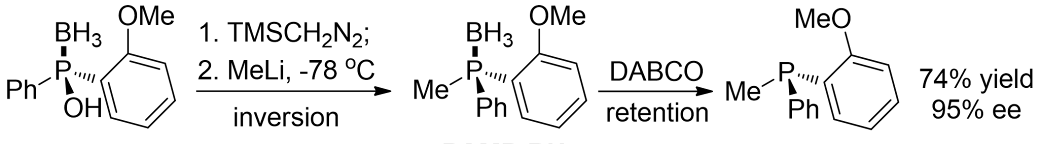

5bf (99% ee) **PAMP-BH$_3$** **PAMP**

To a stirred solution of phosphinous acid-borane (1 equiv.) in methanol (10 mL) was added (trimethylsilyl)diazomethane (2.0 M in hexanes, 2 equiv.) over two minutes at room temperature. Thephosphinite product was filtrated through a short column of silica (hexane/EtOAc) and the solvent removed under vacuum to give the product as an oil. A solution of the substrates **Me-5bf** (1.0 mmol, 1.0 equiv.) in 6 mL THF was prepared in a flame-dried flask under argon atmosphere, which was then added dropwise to methyllithium reagent (2.0 mmol in 1 mL THF, 2.0 equiv.). The resulting mixture was stirred for 2 h while being kept at -30 °C. After ^{31}P NMR analysis of a small aliquot showed complete consumption of substrates **Me-5bf**, the reaction was carefully quenched with slow addition of saturated aqueous NH_4Cl solution (20 mL) and then diluted with water (10 mL) and DCM (40 mL). The layers were separated, and the aqueous layer was washed with DCM (2 x 20mL). The combined organic layers were washed with brine (20 mL), dried over

anhydrousNa_2SO_4, filtered and concentrated. The residue was dissolved in THF (10 mL), to which DABCO (3.0 mmol, 3.0 equiv.) were added. After stirring for 1 h at 50 °C, the reaction was diluted with water (20 mL) and extracted with EtOAc (3 x 40 mL). The combined organic layers were washed with brine (20 mL), dried over anhydrous Na_2SO_4, filtered and concentrated. The residue was purified by silica gel chromatography to afford the desired product.

Data of **PAMP**:

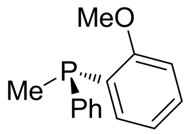

Enantiomeric excess: 95%. Colorless oil, R_f = 0.33 (PE/EA= 5:1), $[\alpha]_D^{25}$ = 30.3° (c = 1.00 in $CHCl_3$); ^1H NMR (300 MHz, $CDCl_3$) δ 7.96–7.78 (m, 1H), 7.72–7.57 (m, 2H), 7.50 (t, *J* = 7.8 Hz, 1H), 7.45–7.30 (m, 3H), 7.06 (t, *J* = 7.5 Hz, 1H), 6.88 (dd, *J* = 8.3, 3.3 Hz, 1H), 3.69 (s, 3H), 1.95 (d, *J* = 10.6 Hz, 3H); ^{13}C NMR (75 MHz, $CDCl_3$) δ 161.37 (d, *J* = 1.2 Hz), 135.63 (d, *J* = 14.6 Hz), 133.81 (d, *J* = 2.1 Hz), 131.16 (d, *J* = 9.8 Hz), 130.41 (d, *J* = 2.4 Hz), 128.35 (d, *J* = 10.3 Hz), 121.06 (d, *J* = 12.3 Hz), 117.23 (d, *J* = 54.6 Hz), 111.21 (d, *J* = 4.1 Hz), 55.41, 10.49 (d, *J* = 42.4 Hz); ^{31}P NMR (122 MHz, $CDCl_3$) δ -36.99; HRMS (ESI-MS) $[M+H]^+$: found231.0861; calculated for $C_{14}H_{16}OP$: 231.0861.

Chiral HPLC: Chiralpak OJ-RH column, Water/Acetonitrile= 60/40, flow rate = 1.0 mL/min, λ = 254 nm.

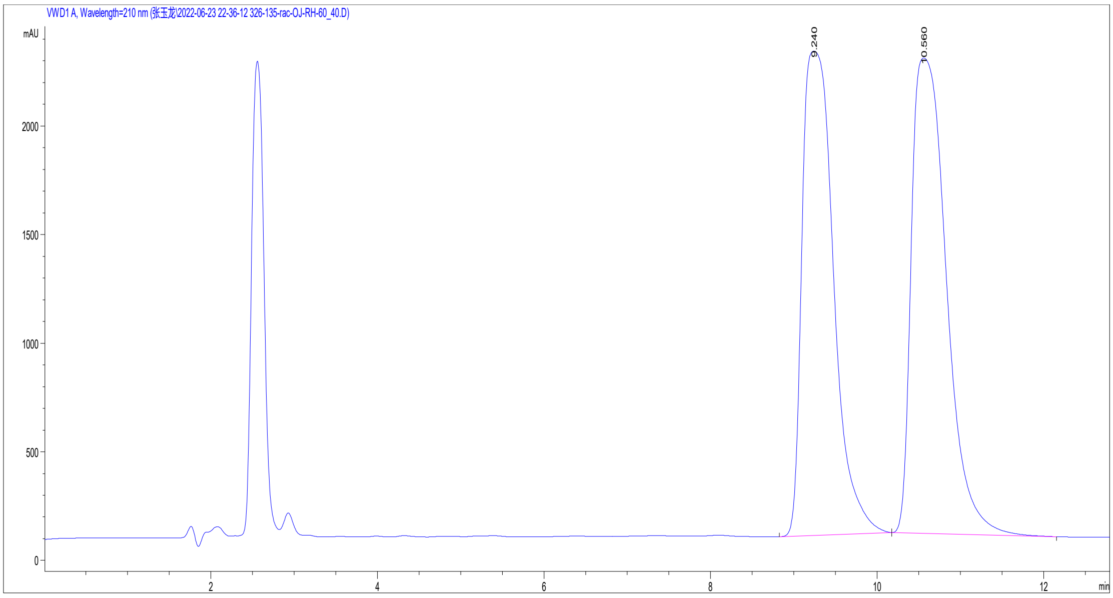

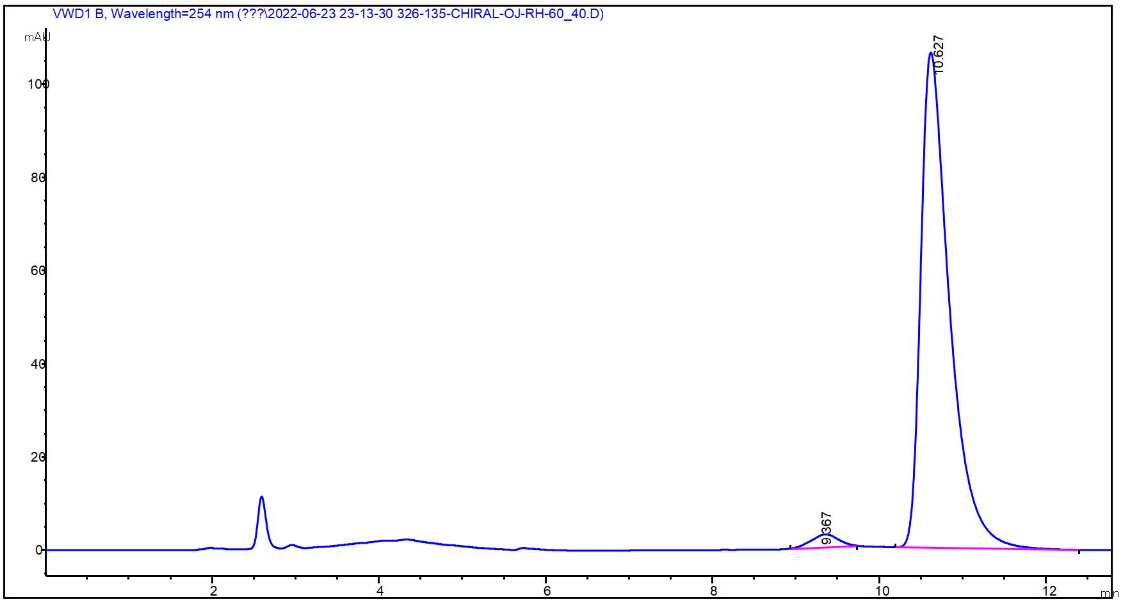

Index	t_R/ (min)	Height / (mv)	Area / (mv.sec)	Area / (%)
1	9.24	2230.1	58670.7	48.3
2	10.56	2187.9	63758.3	51.7
Total				100.0

Index	t_R/ (min)	Height / (mv)	Area / (mv.sec)	Area / (%)
1	9.36	2.8	65.2	2.4
2	10.62	106.3	2550.9	97.6
Total				100.0

General procedure for synthesis of phosphinites-borane 6

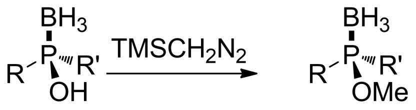

5 **6**

To a stirred solution of phosphinousacid-borane (1 equiv.) in methanol (10 mL) was added (trimethylsilyl)diazomethane (2.0 M in hexanes, 2 equiv.) over two minutes at room temperature. The phosphinite product was filtrated through a short column of silica (hexane/EtOAc) and the solvent removed under vacuum to give the product **6**.

O-methyl phenyl(2-vinylphenyl)phosphinite-borane 6a:

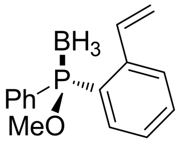

Enantiomeric excess: 99%. 251.0 mg (98% yield), colorless oil, R_f = 0.72 (PE/EA= 10:1), $[\alpha]_D^{25}$ = -72.3 ° (c = 1.00 in $CHCl_3$); ^1H NMR (300 MHz, $CDCl_3$) δ 7.93 (dd, J = 12.9, 7.7 Hz, 1H), 7.70–7.33 (m, 7H), 7.01 (dd, J = 17.1, 11.1 Hz, 1H), 5.55 (d, J = 17.2 Hz, 1H), 5.13 (d, J = 10.9 Hz, 1H), 3.73 (d, J = 12.1 Hz, 3H); ^{13}C NMR (75 MHz, $CDCl_3$) δ 141.56 (d, J = 5.8 Hz), 134.90 (d, J = 5.3 Hz), 134.05 (d, J = 15.8 Hz), 132.55 (d, J = 2.2 Hz), 131.75 (d, J = 2.3 Hz), 131.16 (d, J = 11.3 Hz), 128.58 (d, J = 10.6 Hz), 127.54 (d, J = 11.9 Hz), 126.82 (d, J = 7.6 Hz), 116.86, 53.88 (d, J = 2.6 Hz); ^{31}P NMR (122 MHz, $CDCl_3$) δ 112.8 (q, J = 92.7 Hz); HRMS (ESI-MS) $[M+H]^+$: found257.1190; calculated for $C_{15}H_{19}BOP$: 257.1188.

O-methyl phenyl(dibenzo[b,d]thiophen-4-yl)phosphinite -borane 6b:

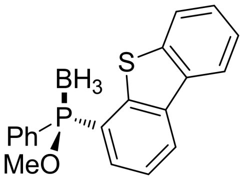

Enantiomeric excess: 99%. 325.9 mg (97% yield), white solid, **m.p.** = 85-90 °C, R_f = 0.69 (PE/EA= 10:1), $[\alpha]_D^{25}$ = -33.4° (c = 1.00 in $CHCl_3$); ^1H NMR (300 MHz, $CDCl_3$) δ 8.33 (d, J = 7.9 Hz, 1H), 8.21–8.03 (m, 2H), 7.88–7.69 (m, 3H), 7.60 (td, J = 7.7, 2.2 Hz, 1H), 7.55–7.39 (m, 5H), 3.86 (d, J = 12.1 Hz, 3H), 1.48–0.74 (m, 3H); ^{13}C NMR (75 MHz, $CDCl_3$) δ 141.09 (d, J = 2.7 Hz), 139.89, 136.76 (d, J = 6.3 Hz), 134.12, 132.77 (d, J = 17.4 Hz), 132.13 (d, J = 2.4 Hz), 131.31 (d, J = 11.5 Hz), 130.35 (d, J = 68.3 Hz), 128.66 (d, J = 10.8 Hz), 127.39, 125.36 (d, J = 2.4 Hz), 124.48 (d, J = 12.1 Hz), 122.52, 121.65, 54.29 (d, J = 2.3 Hz); ^{31}P NMR (122 MHz, $CDCl_3$) δ 110.47 (q, J = 83.8 Hz); HRMS (ESI-MS) $[M+H]^+$: found 337.0912; calculated for $C_{19}H_{19}BOPS$: 337.0909.

General procedure for synthesis of phosphinates 7

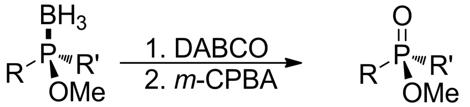

6 **7**

A solution of the substrates **6** (0.2 mmol, 1.0 equiv.) in 6 mL THF was prepared in a flame-dried flask under argon atmosphere, which was then added dropwise to DABCO (0.4 mmol in 1 mL THF, 2.0 equiv.). The resulting mixture was stirred for 2 h while being kept at 50 °C. After ^{31}P NMR analysis of a small aliquot showed complete consumption of substrates **6**, *m*-CPBA (0.6 mmol in 1 mL THF, 3.0 equiv.) were added. After stirring for 1 h, the reaction was diluted with water (20 mL) and extracted with EtOAc (3 x 40 mL). The combined organic layers were washed with brine (20 mL), dried over anhydrous Na_2SO_4, filtered and concentrated. The residue was purified by silica gel chromatography to afford the desired product.

O-methyl phenyl(2-vinylphenyl)phosphinate 7a:

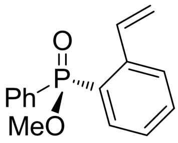

Enantiomeric excess: 99%. 46.6 mg (90% yield), colorless oil, R_f = 0.34 (PE/EA= 1:1), $[\alpha]_D^{25}$ = -83.4 ° (c = 1.00 in $CHCl_3$); ^1H NMR (300 MHz, $CDCl_3$) δ 8.00 (s, 1H), 7.88 (dd, J = 12.6, 7.8 Hz, 2H), 7.69 (dd, J = 13.4, 7.5 Hz, 2H), 7.58–7.50 (m, 1H), 7.45 (t, J = 7.3 Hz, 3H), 7.38 (dd, J = 7.5, 3.5 Hz, 2H), 7.35–7.18 (m, 1H), 5.53 (d, J = 17.2 Hz, 1H), 5.18 (d, J = 11.1 Hz, 1H), 3.73 (d, J = 11.3 Hz, 3H); ^{13}C NMR (75 MHz, $CHCl_3$) δ 169.00 , 141.72 (d, J = 10.4 Hz), 134.85 (d, J = 5.4 Hz), 133.60 (d, J = 9.1 Hz), 133.21, 132.88 (d, J = 2.6 Hz), 132.38 (d, J = 2.9 Hz), 131.58 (d, J = 10.8 Hz), 129.92 (d, J = 33.3 Hz), 128.59 (d, J = 13.5 Hz), 128.18, 127.44 (d, J = 12.7 Hz), 126.72 (d, J = 12.1 Hz), 117.50 , 51.81 (d, J = 6.0 Hz); ^{31}P NMR (121 MHz, $CDCl_3$) δ 35.02; HRMS (ESI-MS) $[M+H]^+$: found 259.0813; calculated for $C_{15}H_{16}O_2P$: 259.0810.

Chiral HPLC: Chiralpak AS-3R column, Water/Acetonitrile= 65/35, flow rate = 1.0 mL/min, λ = 254 nm.

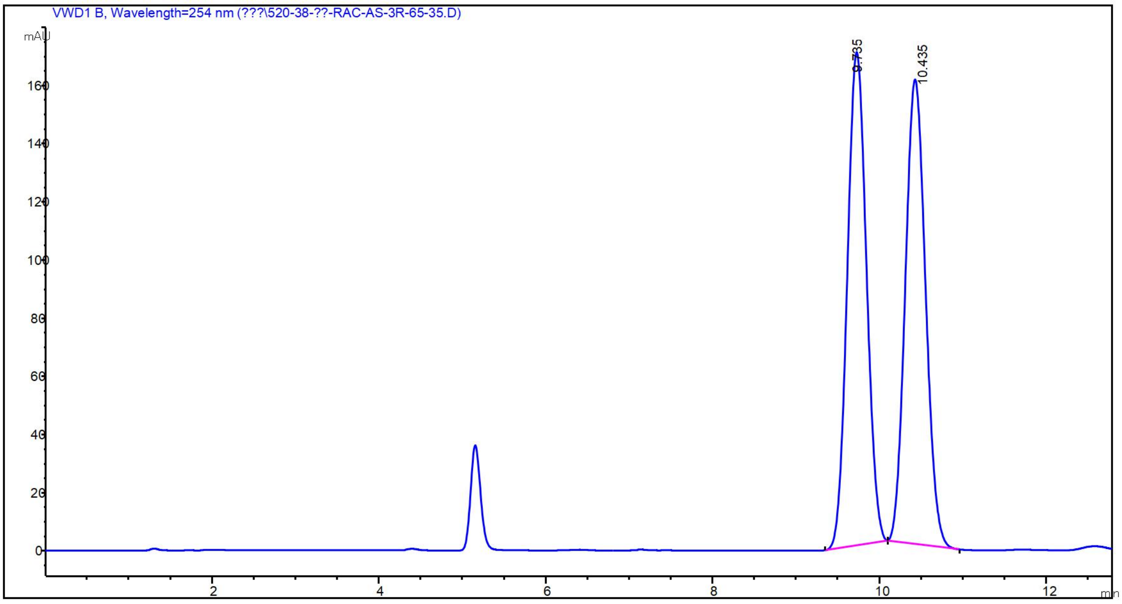

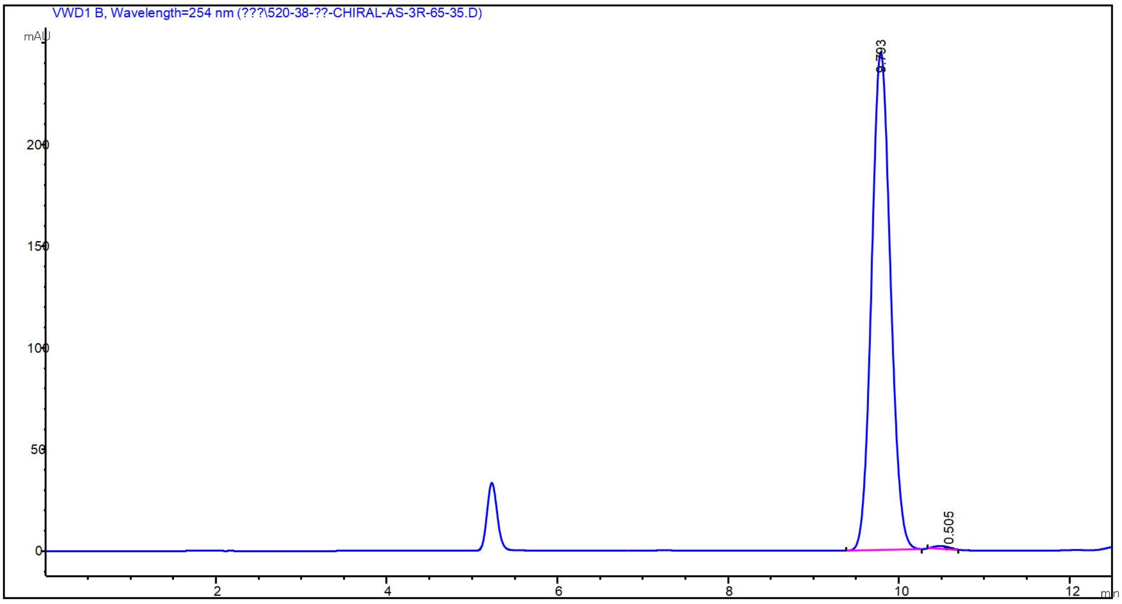

Index	t_R/ (min)	Height / (mv)	Area / (mv.sec)	Area / (%)
1	9.73	169.6	2704.7	50.2
2	10.43	159.6	2688.1	49.8
Total				100.0

Index	t_R/ (min)	Height / (mv)	Area / (mv.sec)	Area / (%)
1	9.79	248	3769.3	99.5
2	10.50	1.5	18.9	0.5
Total				100.0

O-methyl dibenzo[b,d]thiophen-4-yl(phenyl)phosphinate 7b:

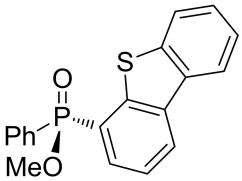

Enantiomeric excess: 99%. 325.9 mg (97% yield), white solid, **m.p.** = 105-110 °C, R_f = 0.69 (PE/EA= 10:1), $[\alpha]_D^{25}$ = -33.4 ° (c = 1.00 in $CHCl_3$); ^1H NMR (300 MHz, $CDCl_3$) δ 8.33 (d, J = 7.9 Hz, 1H), 8.23–8.13 (m, 1H), 8.02 (dd, J = 12.9, 7.4 Hz, 1H), 7.97–7.81 (m, 3H), 7.58 (td, J = 7.7, 2.7 Hz, 1H), 7.47 (dq, J = 10.8, 5.5, 3.6 Hz, 5H), 3.89 (d, J = 11.2 Hz, 3H); ^{13}C NMR (75 MHz, $CDCl_3$) δ 142.03 (d, J = 8.2 Hz), 140.18, 136.67 (d, J = 10.5 Hz), 134.31, 132.55 (d, J = 2.8 Hz), 131.93 (d, J = 8.7 Hz), 131.52 (d, J = 10.5 Hz), 128.58 (d, J = 13.5 Hz), 127.35, 125.38 (d, J = 2.7 Hz), 124.61, 124.37 (d, J = 12.3 Hz), 122.60, 121.57, 51.94 (d, J = 5.9 Hz); ^{31}P NMR (121 MHz, $CDCl_3$) δ 32.36; HRMS (ESI-MS) $[M+H]^+$: found 339.0533; calculated for $C_{19}H_{16}O_2PS$: 339.0530.

Chiral HPLC: Chiralpak AS-3R column, Water/Acetonitrile= 55/45, flow rate = 1.0 mL/min, λ = 254 nm.

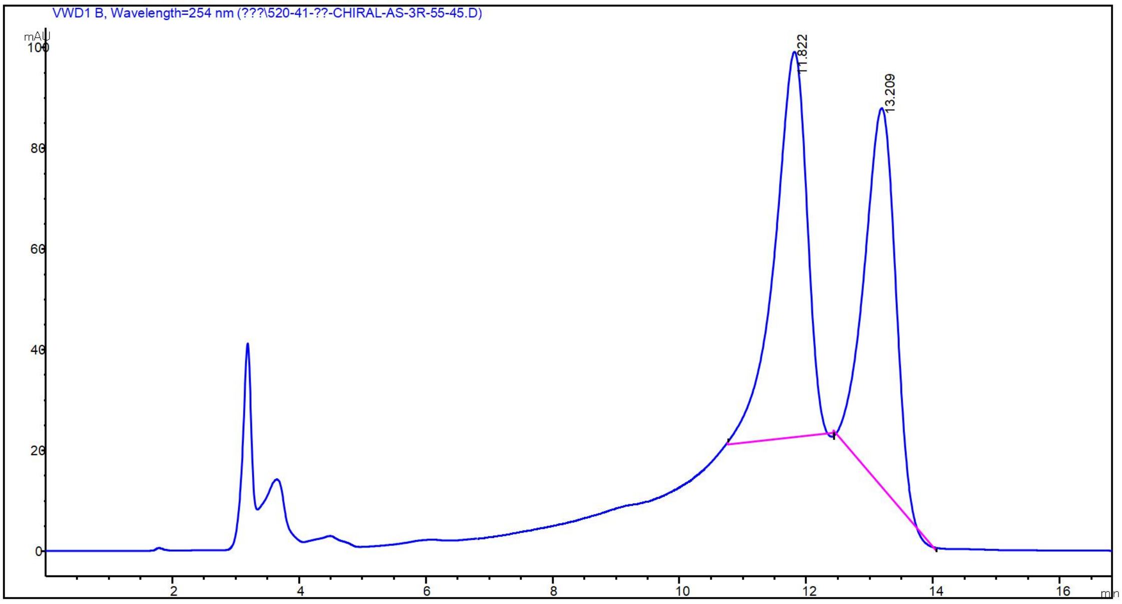

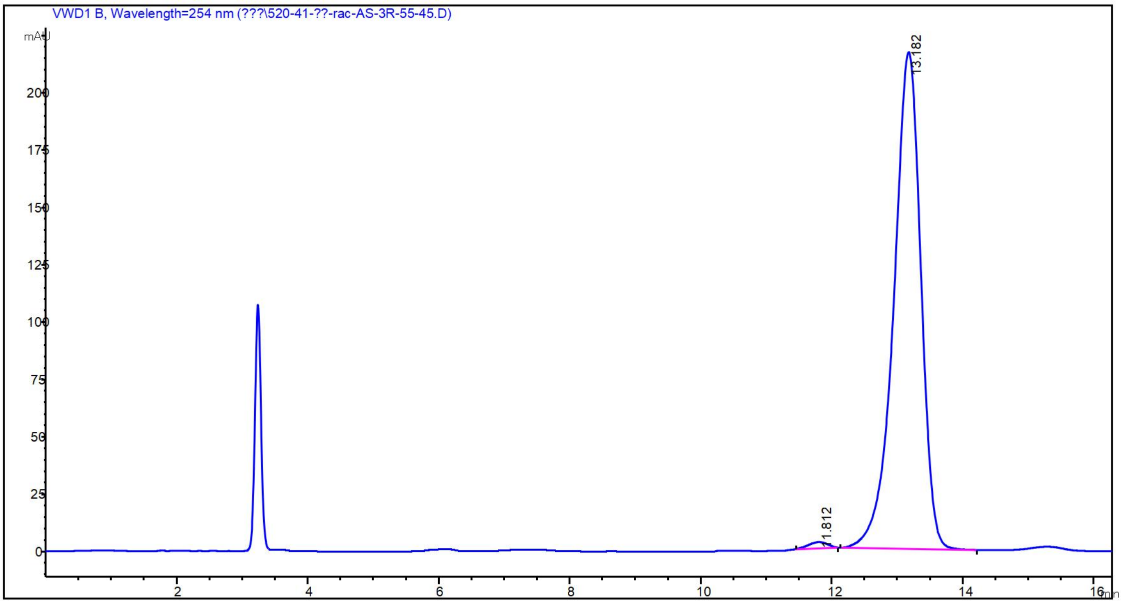

Index	t_R/ (min)	Height / (mv)	Area / (mv.sec)	Area / (%)
1	11.82	76.5	2722.4	51.7
2	13.20	75.3	2538.8	48.3
Total				100.0

Index	t_R/ (min)	Height / (mv)	Area / (mv.sec)	Area / (%)
1	11.81	2.8	59.6	0.9
2	13.18	216.5	6209.7	99.1
Total				100.0

General procedure for synthesis of phosphinothioates 8

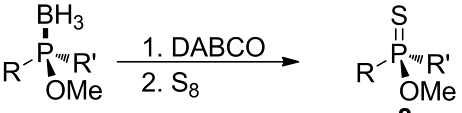

6 **8**

A solution of the substrates **6** (0.2 mmol, 1.0 equiv.) in 6 mL THF was prepared in a flame-dried flask under argon atmosphere, which was then added dropwise to DABCO (0.4 mmol in 1 mL THF, 2.0 equiv.). The resulting mixture was stirred for 2 h while being kept at 50 °C. After ^{31}P NMR analysis of a small aliquot showed complete consumption of substrates **6**, S_8 (0.6 mmol, 3.0 equiv.) were added. After stirring for 1 h, the reaction was diluted with water (20 mL) and extracted with EtOAc (3 x 40 mL). The combined organic layers were washed with brine (20 mL), dried over anhydrous Na_2SO_4, filtered and concentrated. The residue was purified by silica gel chromatography to afford the desired product.

O-methyl phenyl(2-vinylphenyl)phosphinothioate 8a:

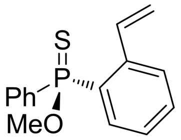

Enantiomeric excess: 99%. 52.1 mg (95% yield), colorless oil, R_f = 0.63 (PE/EA= 10:1), $[\alpha]_D^{25}$ = 4.5° (c = 1.00 in $CHCl_3$); ^1H NMR (300 MHz, $CDCl_3$) δ 7.94 (dd, *J* = 15.0, 7.7 Hz, 1H), 7.79 (dd, *J* = 13.8, 6.9 Hz, 2H), 7.64–7.54 (m, 1H), 7.53–7.48 (m, 2H), 7.45 (dd, *J* = 7.7, 3.5 Hz, 2H), 7.39–7.31 (m, 1H), 7.26 (dd, *J* = 10.1, 7.1 Hz, 1H), 5.53 (d, *J* = 17.2 Hz, 1H), 5.15 (d, *J* = 11.0 Hz, 1H), 3.69 (d, *J* = 14.0 Hz, 3H); ^{13}C NMR (75 MHz, $CDCl_3$) δ 140.66 (d, *J* = 9.9 Hz), 135.12 (d, *J* = 6.2 Hz), 133.96 (d, *J* = 108.2 Hz), 132.90 (d, *J* = 11.2 Hz), 132.30 , 132.14–131.73 (m), 131.37 (d, *J* = 11.7 Hz), 128.44 (d, *J* = 13.5 Hz), 127.31 (d, *J* = 10.3 Hz), 127.15 (d, *J* = 8.5 Hz), 51.04 (d, *J* = 5.5 Hz); ^{31}P NMR (121 MHz, $CDCl_3$) δ 83.55; HRMS (ESI-MS) $[M+H]^+$: found 275.0588; calculated for $C_{15}H_{16}OPS$: 275.0581.

Chiral HPLC: Chiralpak OJ-RH column, Water/Acetonitrile= 40/60, flow rate = 1.0 mL/min, λ = 254 nm.

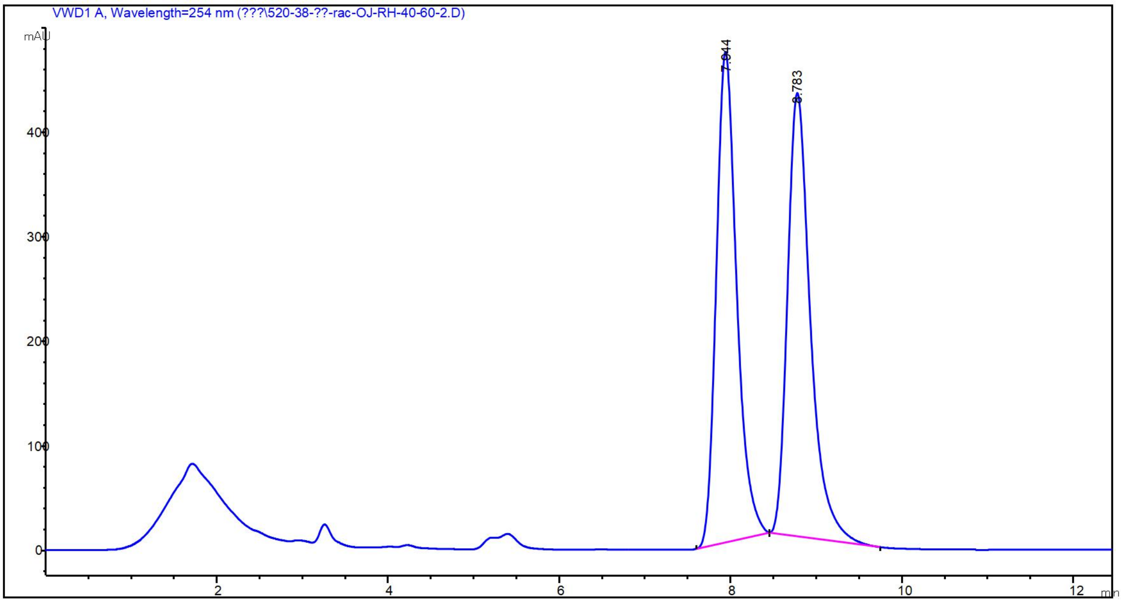

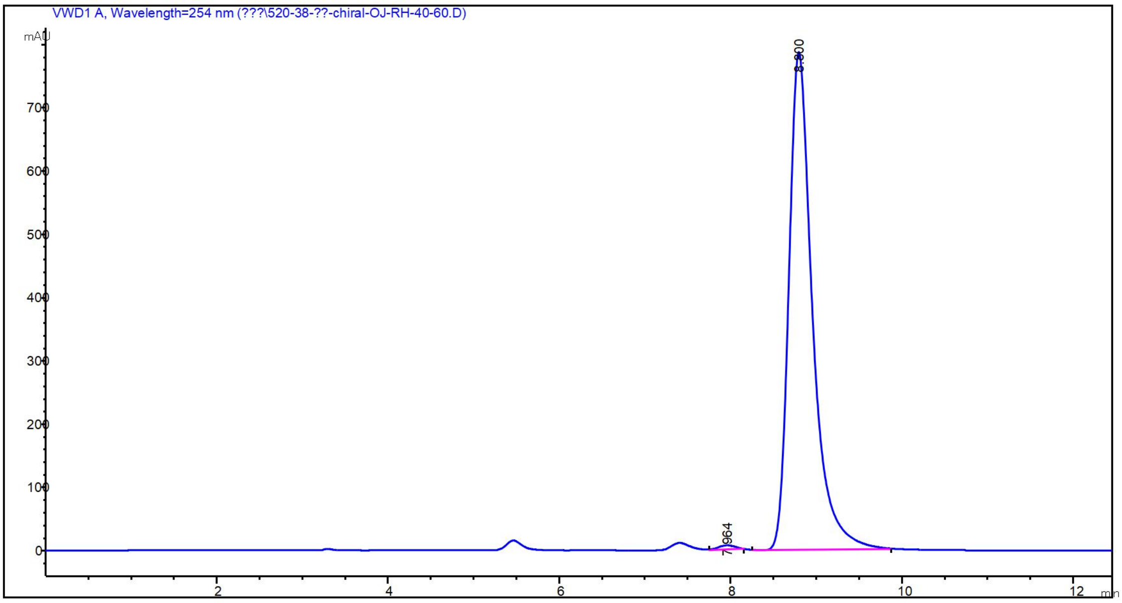

Index	t_R/ (min)	Height / (mv)	Area / (mv.sec)	Area / (%)
1	7.94	469.4	7752.7	49.6
2	8.78	424.0	7876.1	50.4
Total				100.0

Index	t_R/ (min)	Height / (mv)	Area / (mv.sec)	Area / (%)
1	7.96	6.4	82.7	0.5
2	8.80	786.5	15046.5	99.5
Total				100.0

O-methyl dibenzo[b,d]thiophen-4-yl(phenyl)phosphinothioate 8b:

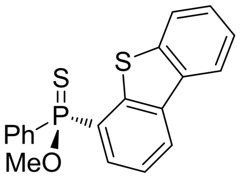

Enantiomeric excess: 98%. 65.8 mg (93% yield), white solid, **m.p.** = 105-110 °C, R_f = 0.69 (PE/EA= 10:1), $[\alpha]_D^{25}$ = 10.4° (c = 1.00 in $CHCl_3$); ^1H NMR (300 MHz, $CDCl_3$) δ 8.31 (d, *J* = 7.9 Hz, 1H), 8.22–8.08 (m, 2H), 8.01–7.85 (m, 2H), 7.87–7.76 (m, 1H), 7.60 (td, *J* = 7.7, 2.6 Hz, 1H), 7.54–7.36 (m, 5H), 3.84 (d, *J* = 13.9 Hz, 3H); ^{13}C NMR (75 MHz, $CDCl_3$) δ 140.65 (d, *J* = 8.1 Hz), 140.12, 136.85 (d, *J* = 10.1 Hz), 134.17, 132.20 (d, *J* = 3.1 Hz), 131.99 (d, *J* = 10.9 Hz), 131.28 (d, *J* = 11.8 Hz), 128.46 (d, *J* = 13.7 Hz), 127.33, 125.13 (d, *J* = 3.0 Hz), 124.60, 124.35 (d, *J* = 13.0 Hz), 122.49, 121.60, 68.02 , 25.65; ^{31}P NMR (121 MHz, $CDCl_3$) δ 81.89; HRMS (ESI-MS) $[M+H]^+$: found 355.0300; calculated for $C_{19}H_{16}OPS_2$: 355.0302.

Chiral HPLC: Chiralpak OJ-RH column, Water/Acetonitrile= 40/60, flow rate = 1.0 mL/min, λ = 254 nm.

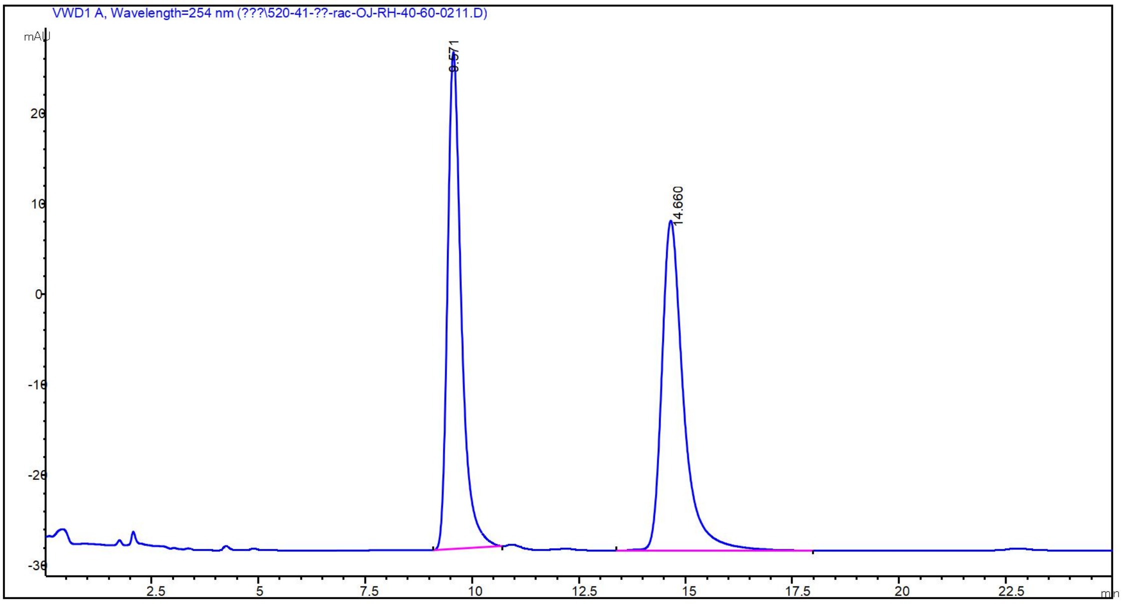

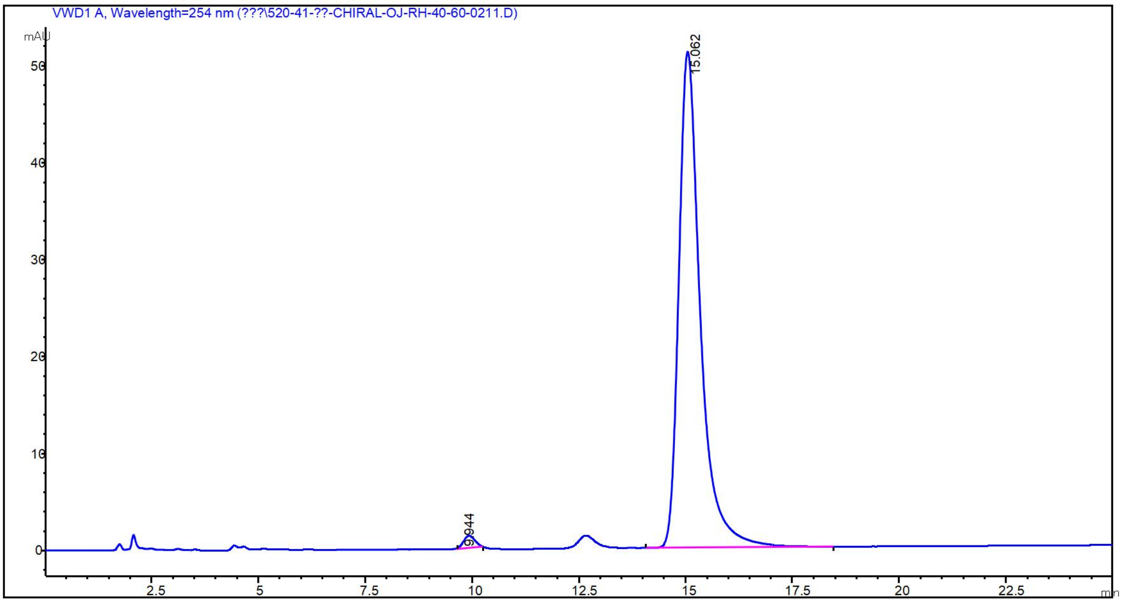

Index	t_R/ (min)	Height / (mv)	Area / (mv.sec)	Area / (%)
1	9.57	54.9	1235.6	49.1
2	14.66	36.4	1279.4	50.9
Total				100.0

Index	t_R/ (min)	Height / (mv)	Area / (mv.sec)	Area / (%)
1	9.94	1.2	22.7	1.2
2	15.06	51.1	1793.8	98.8
Total				100.0

General procedure for synthesis of phosphines 9

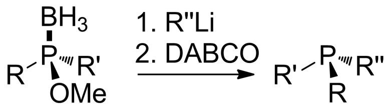

6 **9**

A solution of the substrates **6** (1.0 mmol, 1.0 equiv.) in 6 mL THF was prepared in a flame-dried flask under argon atmosphere, which was then added dropwise to organolithium reagent (2.0 mmol in 1 mL THF, 2.0 equiv.). The resulting mixture was stirred for 2 h while being kept at -30 °C. After ^{31}P NMR analysis of a small aliquot showed complete consumption of substrates **6**, the reaction was carefully quenched with slow addition of saturated aqueous NH_4Cl solution (20 mL) and then diluted with water (10 mL) and DCM (40 mL). The layers were separated, and the aqueous layer was washed with DCM (2 x 20mL). The combined organic layers were washed with brine (20 mL), dried over anhydrous Na_2SO_4, filtered and concentrated. The residue was dissolved in THF (10 mL), to which DABCO (3.0 mmol, 3.0 equiv.) were added. After stirring for 1 h at 50 °C, the reaction was diluted with water (20 mL) and extracted with EtOAc (3 x 40 mL). The combined organic layers were washed with brine (20 mL), dried over anhydrous Na_2SO_4, filtered and concentrated. The residue was purified by silica gel chromatography to afford the desired

product.

Dibenzo[b,d]thiophen-4-yl(methyl)(phenyl)phosphine 9b:

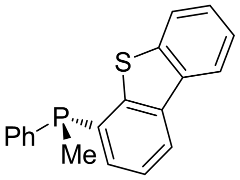

Enantiomeric excess: 96%. 284.5 mg (93% yield), white solid, **m.p.** = 112–114 °C, R_f = 0.69 (PE/EA= 10:1), $[\alpha]_D^{25}$ = 47.2° (c = 1.00 in $CHCl_3$); ^{1}H NMR (300 MHz, $CDCl_3$) δ 8.23–8.07 (m, 2H), 7.84 (dd, J = 6.1, 3.0 Hz, 1H), 7.57–7.41 (m, 6H), 7.33 (d, J = 3.4 Hz, 3H), 1.78 (d, J = 3.5 Hz, 3H); ^{13}C NMR (75 MHz, $CDCl_3$) δ 145.19 (d, J = 27.8 Hz), 139.77 (d, J = 6.3 Hz), 138.19 (d, J = 10.4 Hz), 135.44 (d, J = 5.6 Hz), 133.44 (d, J = 12.9 Hz), 132.14 (d, J = 18.4 Hz), 128.91 (d, J = 2.4 Hz), 128.71, 128.54 (d, J = 6.7 Hz), 126.84 , 124.83 (d, J = 1.9 Hz), 124.38, 122.82 , 121.98 , 121.71 , 11.29 (d, J = 13.3 Hz); ^{31}P NMR (121 MHz, $CDCl_3$) δ -30.63; HRMS (ESI-MS) $[M+H]^+$: found 307.0638; calculated for $C_{19}H_{16}PS$: 307.0632.

Chiral HPLC: Chiralpak OJ-RH column, Water/Acetonitrile= 40/60, flow rate = 1.0 mL/min, λ = 254 nm.

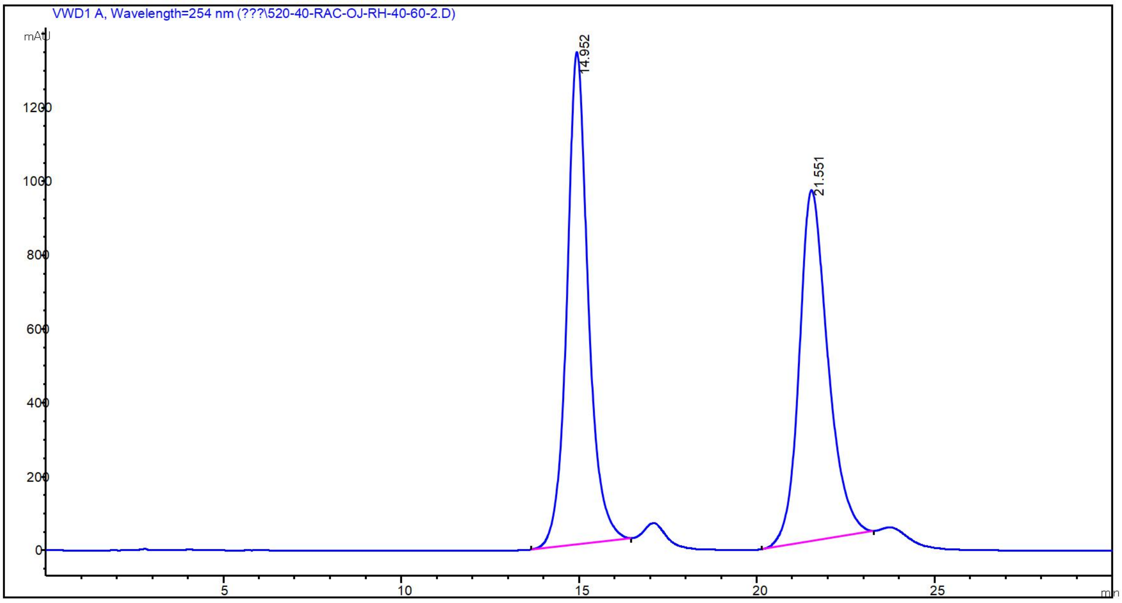

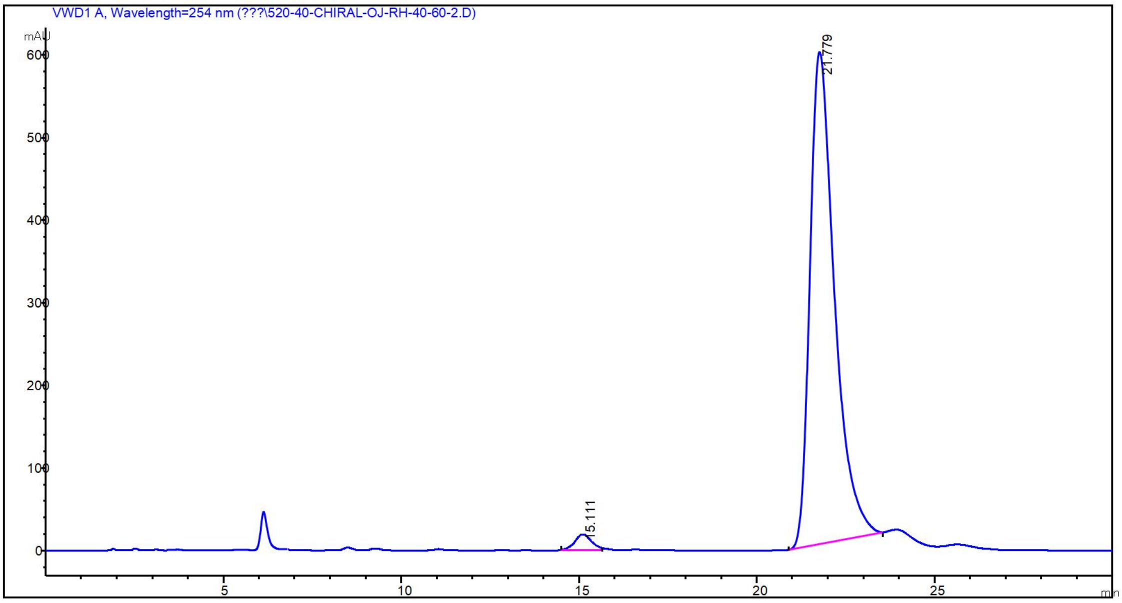

Index	t_R/ (min)	Height / (mv)	Area / (mv.sec)	Area / (%)
1	14.95	1333.7	55625.7	51.0
2	21.55	949.9	53266.1	49.0
Total				100.0

Index	t_R/ (min)	Height / (mv)	Area / (mv.sec)	Area / (%)
1	15.11	19.4	642.4	2.1
2	21.77	595.9	28646.2	97.9

General procedure for synthesis of phosphine oxides 10

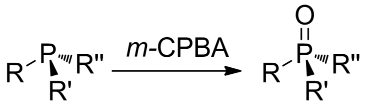

9 **10**

A solution of the substrates **9** (0.2 mmol, 1.0 equiv.) in 6 mL THF was prepared in a flame-dried flask under argon atmosphere, which was then added dropwise to *m*-CPBA (0.4 mmol in 1 mL THF, 2.0 equiv.). The resulting mixture was stirred for 1 h while being kept at rt. After ^{31}P NMR analysis of a small aliquot showed complete consumption of substrates **9**, the reaction was diluted with water (20 mL) and extracted with EtOAc (3 x 40 mL). The combined organic layers were washed with brine (20 mL), dried over anhydrous Na_2SO_4, filtered and concentrated. The residue was purified by silica gel chromatography to afford the desired product.

Dibenzo[b,d]thiophen-4-yl(methyl)(phenyl)phosphine oxide 10b:

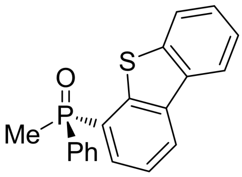

Enantiomeric excess: 96%. 59.9 mg (93% yield), white solid, **m.p.** = 103-107 °C, R_f = 0.54 (PE/EA= 1:1), $[\alpha]_D^{25}$ = = -74.3° (c = 1.00 in $CHCl_3$); ^1H NMR (300 MHz, $CDCl_3$) δ 8.32 (d, J = 7.9 Hz, 1H), 8.17 (dd, J = 6.0, 3.1 Hz, 1H), 7.81 (dt, J = 12.5, 7.0 Hz, 4H), 7.66–7.40 (m, 6H), 2.22 (d, J = 13.3 Hz, 3H); ^{13}C NMR (75 MHz, $CDCl_3$) δ 141.53 (d, J = 6.8 Hz), 140.28 , 136.85 (d, J = 8.9 Hz), 134.07, 132.17 (d, J = 2.7 Hz), 130.76 (d, J = 10.2 Hz), 129.86 (d, J = 9.3 Hz), 128.76 (d, J = 12.1 Hz), 127.34 , 124.89 (d, J = 2.7 Hz), 124.58, 124.23 (d, J = 11.4 Hz), 122.62, 121.59, 15.62 (d, J = 74.1 Hz); ^{31}P NMR (121 MHz, $CDCl_3$) δ 30.93; HRMS (ESI-MS) $[M+H]^+$: found 323.0584; calculated for $C_{19}H_{16}OPS$: 323.0581.

Chiral HPLC: Chiralpak OJ-RH column, Water/Acetonitrile= 60/40, flow rate = 1.0 mL/min, λ = 254 nm.

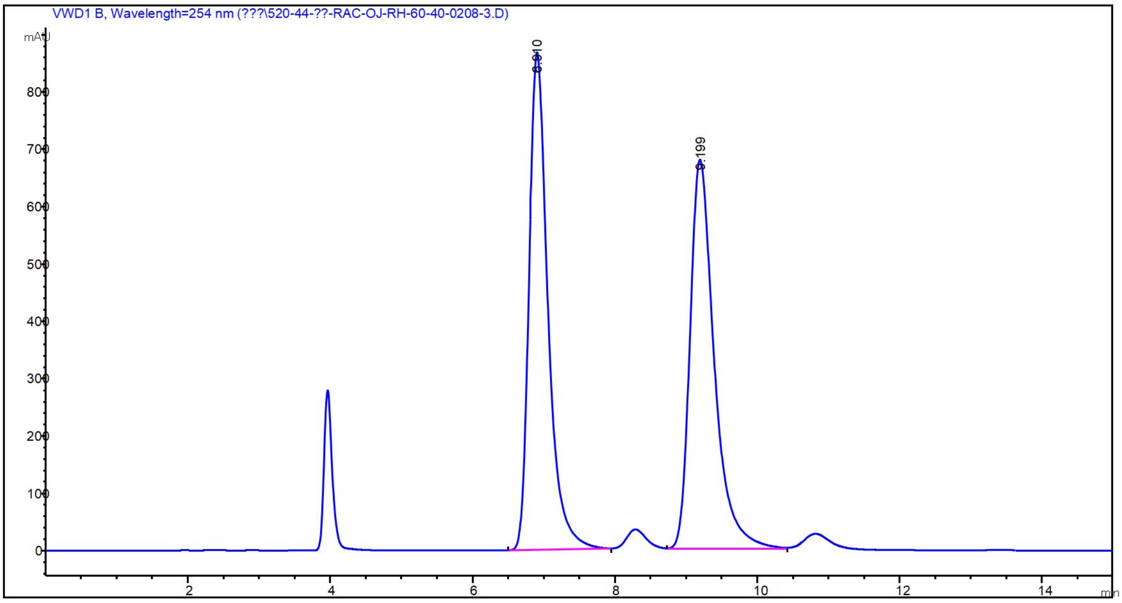

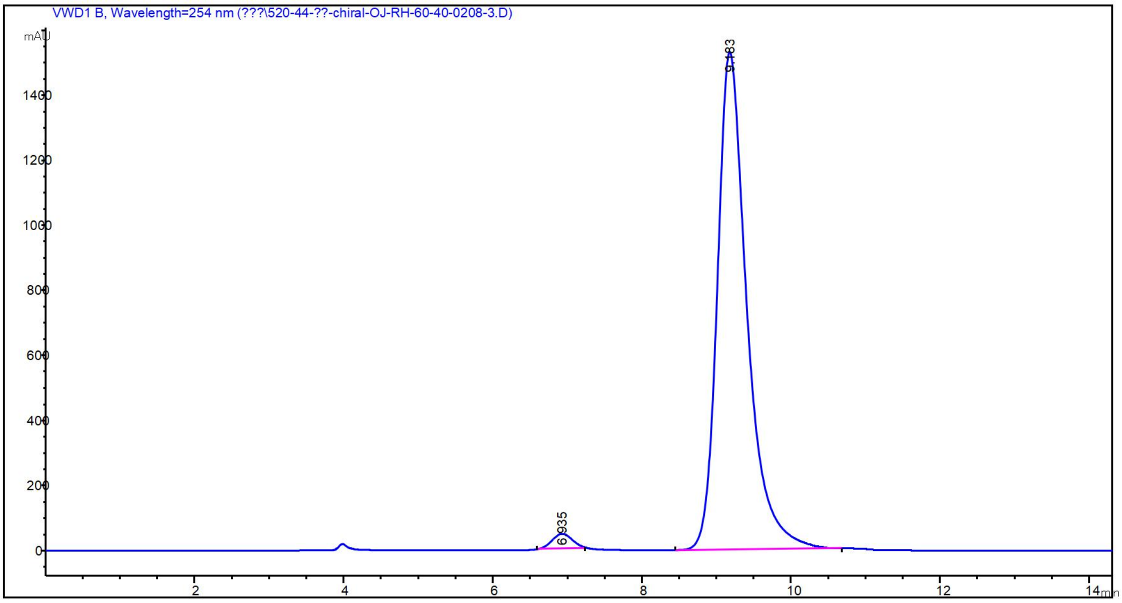

Index	t_R/ (min)	Height / (mv)	Area / (mv.sec)	Area / (%)
1	6.91	867.8	15943.3	50.0
2	9.19	677.7	15908.7	50.0
Total				100.0

Index	t_R/ (min)	Height / (mv)	Area / (mv.sec)	Area / (%)
1	6.93	44.5	817.7	1.9
2	9.18	1529.8	42052.8	98.1
Total				100.0

General procedure for synthesis of phosphine sulfides 11

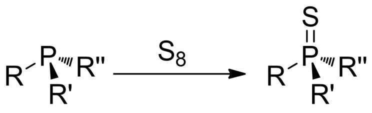

9 11

A solution of the substrates **9** (0.2 mmol, 1.0 equiv.) in 6 mL THF was prepared in a flame-dried flask under argon atmosphere, which was then added dropwise to S_8 (0.4 mmol, 2.0 equiv.). The resulting mixture was stirred for 1 h while being kept at r.t.. After ^{31}P NMR analysis of a small aliquot showed complete consumption of substrates **9**, the reaction was diluted with water (20 mL) and extracted with EtOAc (3 x 40 mL). The combined organic layers were washed with brine (20 mL), dried over anhydrous Na_2SO_4, filtered and concentrated. The residue was purified by silica gel chromatography to afford the desired product.

Dibenzo[b,d]thiophen-4-yl(methyl)(phenyl)phosphine sulfide 11b:

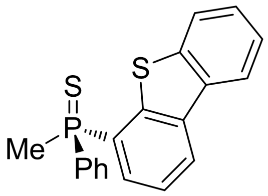

Enantiomeric excess: 96%. 62.9 mg (93% yield), white solid, **m.p.** = 93-98 °C, R_f = 0.68 (PE/EA= 10:1), $[\alpha]_D^{25}$ = 12.3° (c = 1.00 in $CHCl_3$); ^1H NMR (300 MHz, $CDCl_3$) δ 8.31 (d, J = 7.9 Hz, 1H), 8.21–8.11 (m, 1H), 7.96 (dd, J = 14.5, 7.4 Hz, 1H), 7.86 (dd, J = 13.7, 6.8 Hz, 2H), 7.80–7.72 (m, 1H), 7.60 (td, J = 7.7, 1.8 Hz, 1H), 7.55–7.38 (m, 5H), 2.46 (d, J = 13.3 Hz, 3H); ^{13}C NMR (75 MHz, $CDCl_3$) δ 141.03 (d, J = 7.3 Hz), 140.05 , 137.17 (d, J = 8.8 Hz), 134.04 , 131.88 (d, J = 3.0 Hz), 131.15 (d, J = 11.1 Hz), 130.10 (d, J = 9.8 Hz), 128.79 (d, J = 12.5 Hz), 127.38 , 124.81 (d, J = 2.9 Hz), 124.62 , 124.32 (d, J = 11.8 Hz), 122.53 , 121.65, 20.88 (d, J = 60.0 Hz); ^{31}P NMR (121 MHz, $CDCl_3$) δ 35.03; HRMS (ESI-MS) $[M+H]^+$: found 339.0350; calculated for $C_{19}H_{16}PS_2$: 339.0353.

Chiral HPLC: Chiralpak 96% *ee* (Chiralcel OJ-RH column, Water/Acetonitrile= 60/40, flow rate = 1.0 mL/min, λ = 254 nm.

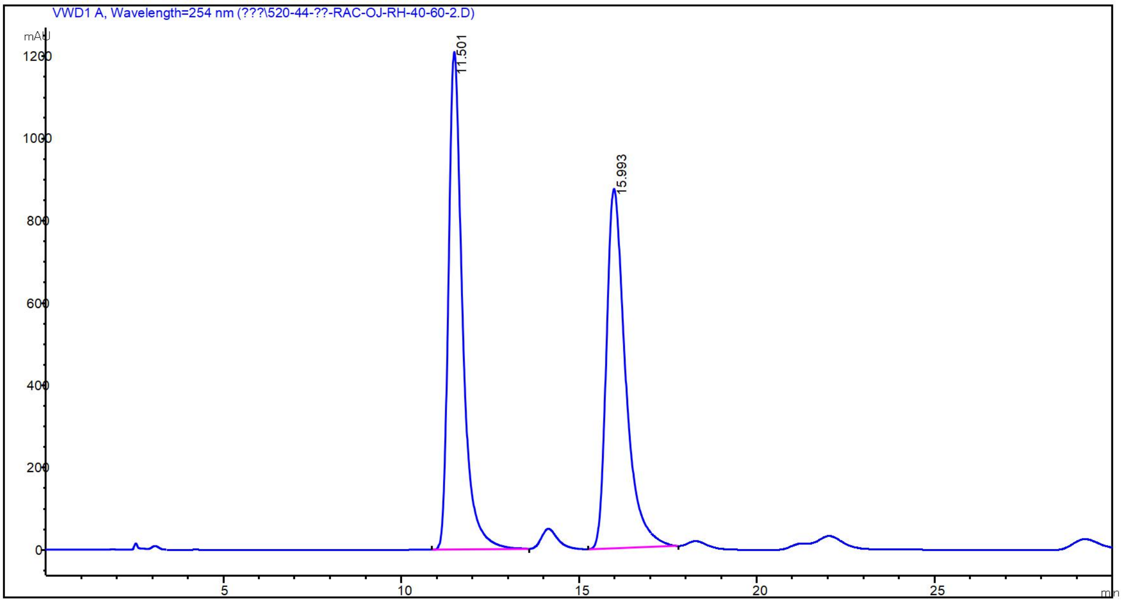

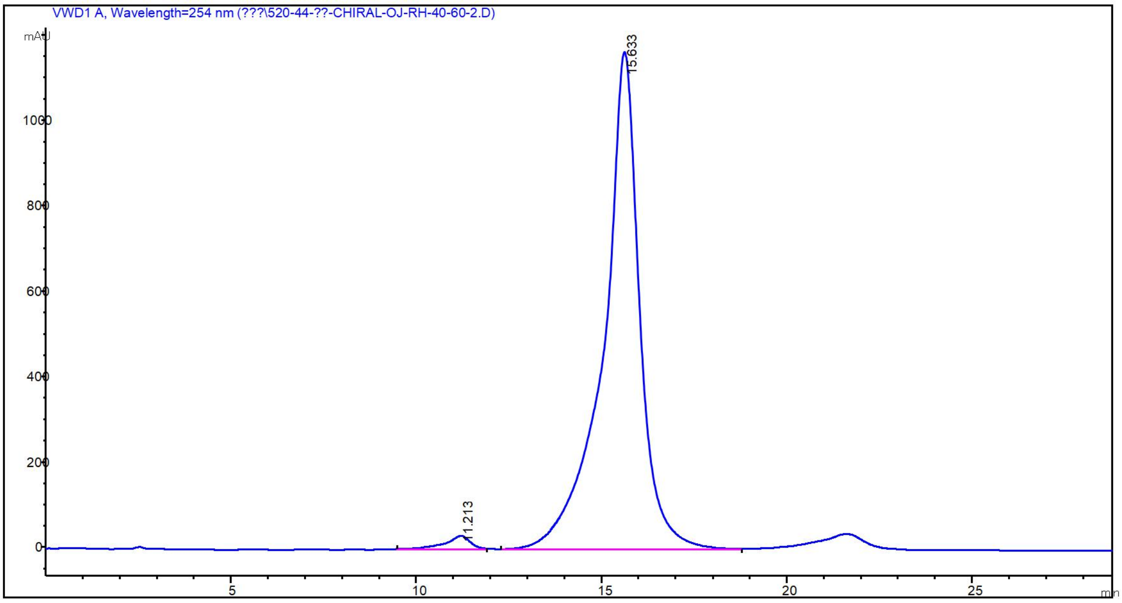

Index	t_R/ (min)	Height / (mv)	Area / (mv.sec)	Area / (%)
1	11.50	1209.3	33172.9	50.4
2	15.99	872.9	32671.4	49.6
Total				100.0

Index	t_R/ (min)	Height / (mv)	Area / (mv.sec)	Area / (%)
1	11.21	31.0	1516.2	1.9
2	15.63	1163.6	78719.7	98.1
Total				100.0

General procedure for synthesis of Secondary Phosphine Oxide 12

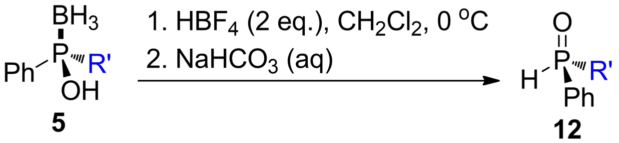

5 12

A solution of the phosphinous acid borane **5** (0.2 mmol, 1.0 equiv.) was dissolved in CH_2Cl_2 (15 mL) and $HBF_4{\cdot}OEt_2$ (0.4 mmol, 2.0 equiv.) was added dropwise at 0 ºC. The solution was stirred at this temperature for 30 min, saturated aqueous solution of $NaHCO_3$ (7 mL) was added dropwise and the solution was stirred a further 10 min at room temperature. The two phases were separated, the aqueous phase was extracted with CH_2Cl_2 (10 x 3 mL) and the combined organic layers were washed with brine (10 mL). The organic layer was dried over Na2SO4, filtered and concentrated. The residue was purified by silica gel chromatography to afford the desired product.

(*R*)-dibenzo[b,d]thiophen-4-yl(phenyl)phosphine oxide 12a:

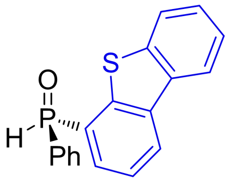

Enantiomeric excess: 98%. 55.4 mg (90% yield), white solid, **m.p.** = 103-108 °C, R_f = 0.30 (PE/EA= 1:2), $[\alpha]_D^{25}$ = -15.8° (c = 1.00 in $CHCl_3$); ^{1}H NMR (300 MHz, $CDCl_3$) δ 9.11 (s, 1H), 8.29 (d, *J* = 7.8 Hz, 1H), 8.19 – 8.02 (m, 1H), 7.98 – 7.67 (m, 4H), 7.65 – 7.34 (m, 6H); ^{13}C NMR (75 MHz, $CDCl_3$) δ 141.15 (d, *J* = 7.6 Hz), 139.72 , 136.80 (d, *J* = 9.0 Hz), 134.08 , 132.88 (d, *J* = 2.8 Hz), 131.37 , 130.86 (d, *J* = 11.9 Hz), 129.88 (d, *J* = 10.4 Hz), 128.97 (d, *J* = 13.1 Hz), 127.51 , 125.80 , 125.48 (d, *J* = 2.8 Hz), 124.82 , 124.45 (d, *J* = 12.4 Hz), 122.74 , 121.71; ^{31}P NMR (121 MHz, $CDCl_3$) δ 19.38; HRMS (ESI-MS) $[M+H]^+$: found 309.0427; calculated for $C_{18}H_{14}OPS$: 309.0425.

Chiral HPLC: Chiralpak 98% *ee* (Chiralcel OJ-RH column, Water/Acetonitrile = 70/30, flow rate = 1.0 mL/min, λ = 254 nm)

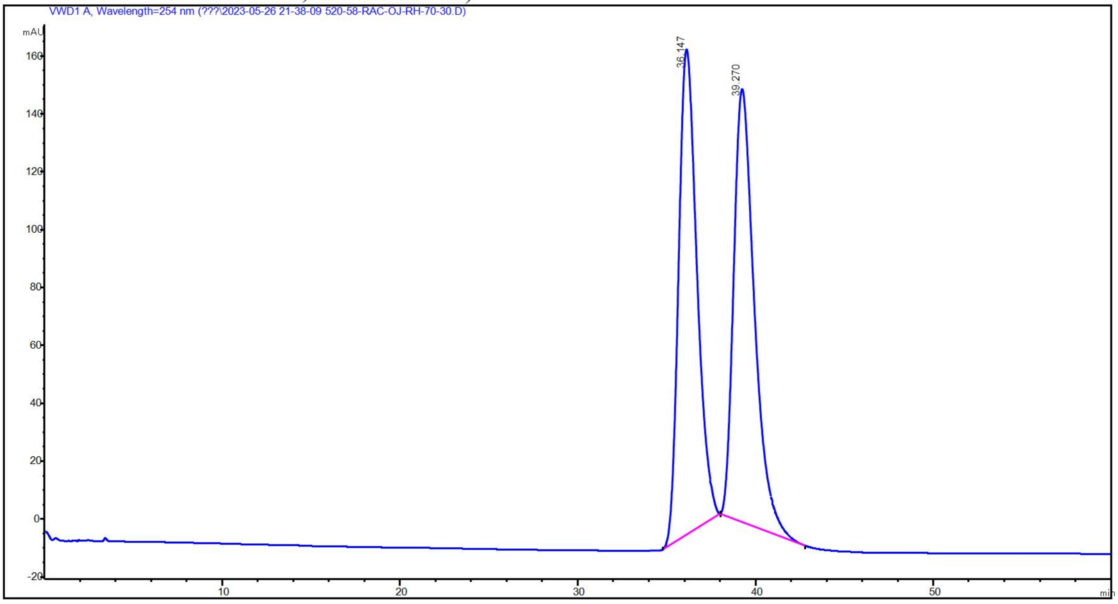

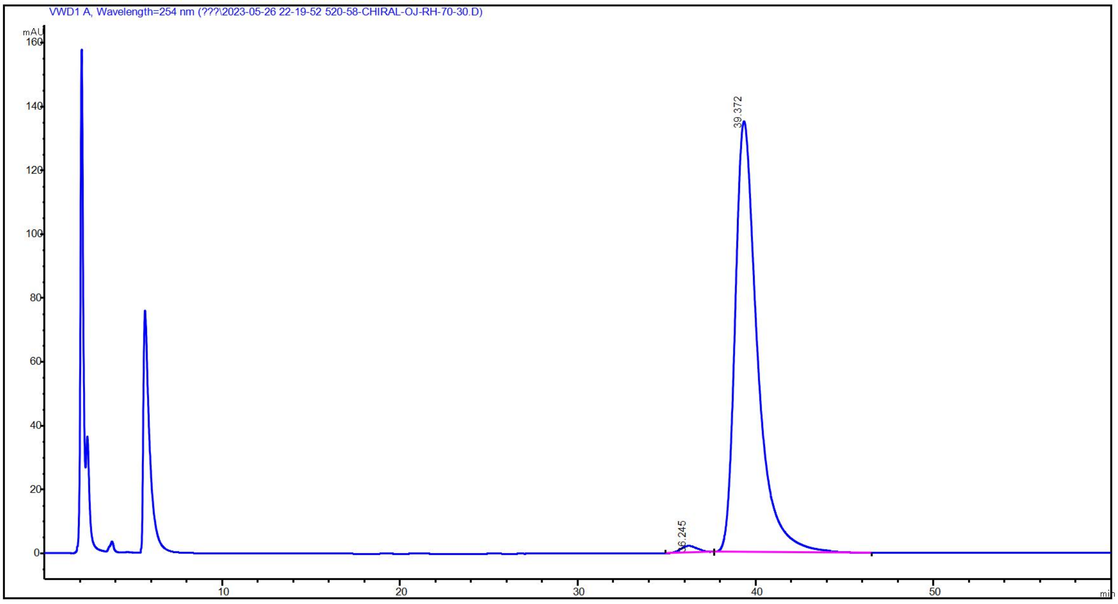

Index	t_R / (min)	Height / (mv)	Area /	Area / (%)
1	36.14	167.6	11996.9	49.8
2	39.27	149.6	12090.3	50.2
Total				100.0

Index	t_R / (min)	Height / (mv)	Area /	Area / (%)
1	36.24	2.2	146.2	1.2
2	39.37	135	11721.1	98.8
Total				100.0

(*R*)-dibenzo[b,d]furan-4-yl(phenyl)phosphine oxide 12b:

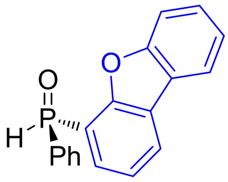

Enantiomeric excess: 97%. 49.6 mg (85% yield), white solid, **m.p.** = 90-94 °C, R_f = 0.25 (PE/EA= 1:2), $[\alpha]_D^{25}$ = -38.4° (c = 1.00 in $CHCl_3$); ^1H NMR (300 MHz, $CDCl_3$) δ 9.35 (s, 0.5H), 8.10 (d, *J* = 7.0 Hz, 1H), 7.88 (dd, *J* = 17.4, 7.4 Hz, 4H), 7.70 (s, 0.5H), 7.62 – 7.39 (m, 5H), 7.35 (d, *J* = 7.1 Hz, 1H); ^{13}C NMR (75 MHz, $CDCl_3$) 156.21 , 156.06 – 155.69 (m), 132.64 (d, *J* = 2.9 Hz), 131.79 , 130.82 – 130.26 (m), 129.58 (d, *J* = 6.1 Hz), 128.87 (d, *J* = 13.3 Hz), 127.97 , 125.60 – 125.05 (m), 124.81 (d, *J* = 6.5 Hz), 123.45 , 123.25 , 123.06 (d, *J* = 7.5 Hz), 120.89 , 115.26 (d, *J* = 99.8 Hz), 112.03; ^{31}P NMR (121 MHz, $CDCl_3$) δ 11.46; HRMS (ESI-MS) $[M+H]^+$: found 293.0658; calculated for $C_{18}H_{14}O_2P$: 293.0653.

Chiral HPLC: Chiralpak 97% *ee* (Chiralcel OD-RH column, Water/Acetonitrile = 40/60, flow rate = 1.0 mL/min, λ = 254 nm)

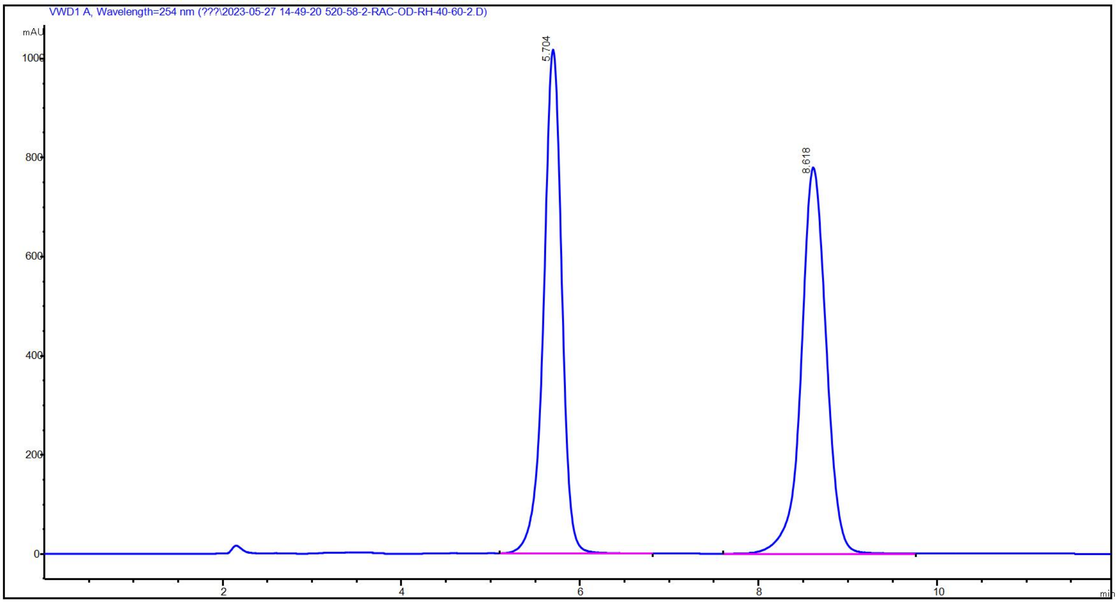

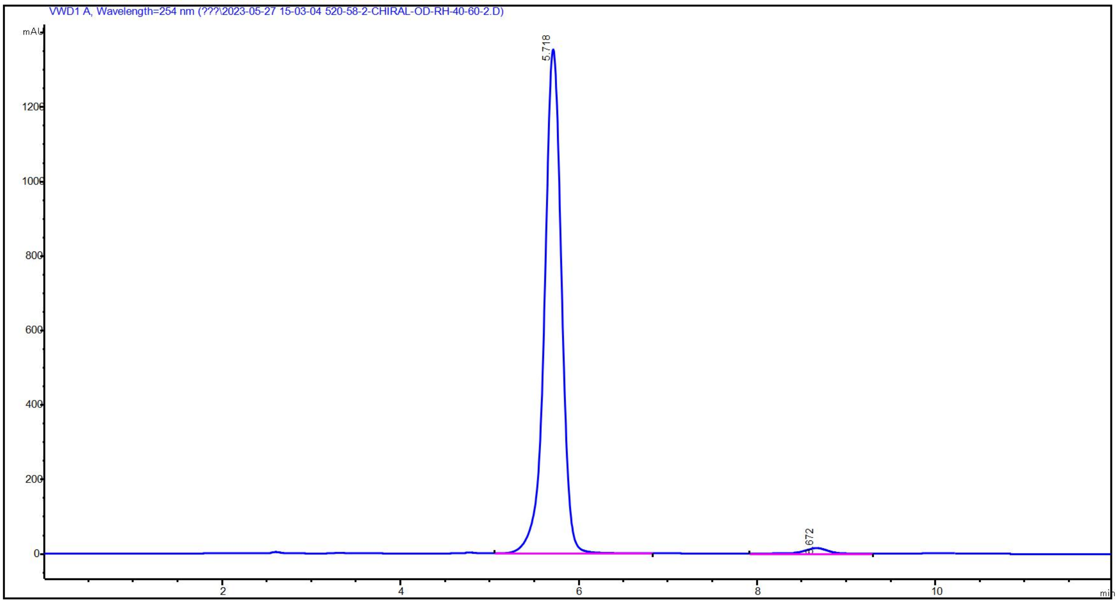

Index	t_R / (min)	Height / (mv)	Area /	Area / (%)
1	5.70	1016.7	14362.9	48.7
2	8.61	778.9	15124.9	51.29
Total				100.0

Index	t_R / (min)	Height / (mv)	Area /	Area / (%)
1	5.70	1354.0	17907.6	98.6
2	8.67	14.4	255.3	1.4
Total				100.0

(*R*)-naphthalen-2-yl(phenyl)phosphine oxide 12c:

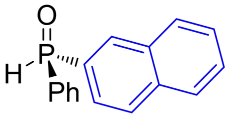

Enantiomeric excess: 98%. 44.3 mg (88% yield), white solid, **m.p.** = 78-82 °C, R_f = 0.30 (PE/EA= 1:2), $[\alpha]_D^{25}$ = -20.4° (c = 1.00 in $CHCl_3$); ^{1}H NMR (300 MHz, $CDCl_3$) δ 8.99 (s, 0.5H), 8.34 (d, *J* = 15.7 Hz, 1H), 7.88 (dd, *J* = 16.9, 7.9 Hz, 3H), 7.72 (dd, *J* = 13.8, 8.2 Hz, 2H), 7.64 – 7.41 (m, 6H), 7.39 (s, 0.5H); ^{13}C NMR (151 MHz MHz, $CDCl_3$) δ 135.09 (d, *J* = 2.4 Hz), 132.86 (d, *J* = 10.9 Hz), 132.64 (d, *J* = 2.8 Hz), 132.53 (d, *J* = 14.1 Hz), 131.46 (d, *J* = 101.7 Hz), 130.79 (d, *J* = 11.5 Hz), 129.03 – 128.89 (m), 128.85 (d, *J* = 4.3 Hz), 128.62 , 128.47 , 128.09 – 127.90 (m), 127.20 , 125.07 (d, *J* = 12.5 Hz); ^{31}P NMR (121 MHz, $CDCl_3$) δ 21.71; HRMS (ESI-MS) $[M+H]^+$: found 253.2478; calculated for $C_{16}H_{14}OP$: 253.2476.
Chiral HPLC: Chiralpak 98% *ee* (Chiralcel AS-RH column, Water/Acetonitrile = 60/40, flow rate = 1.0 mL/min, λ = 254 nm)

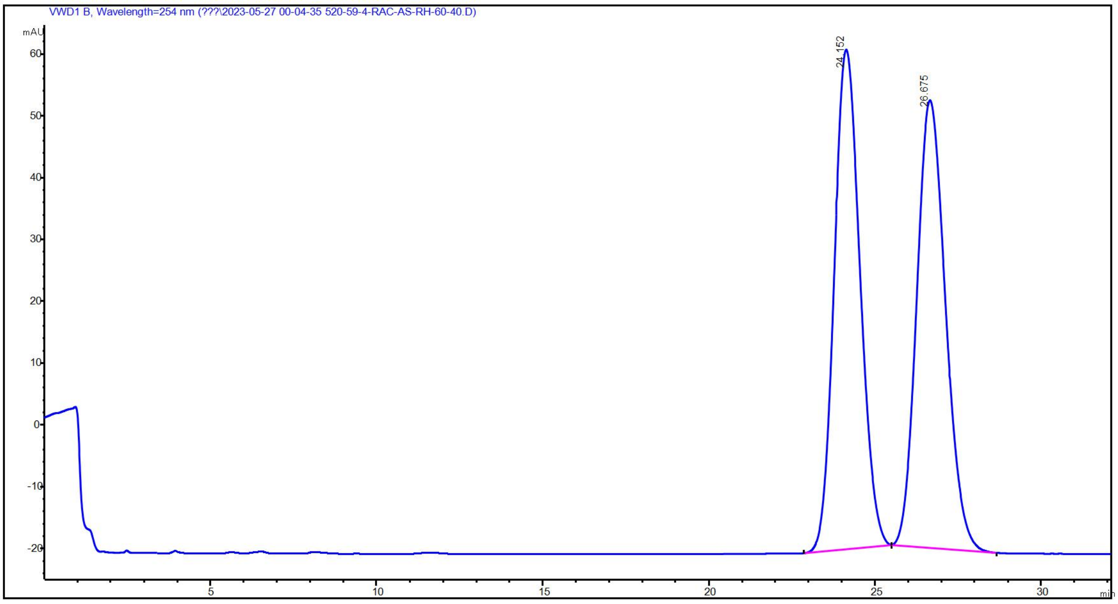

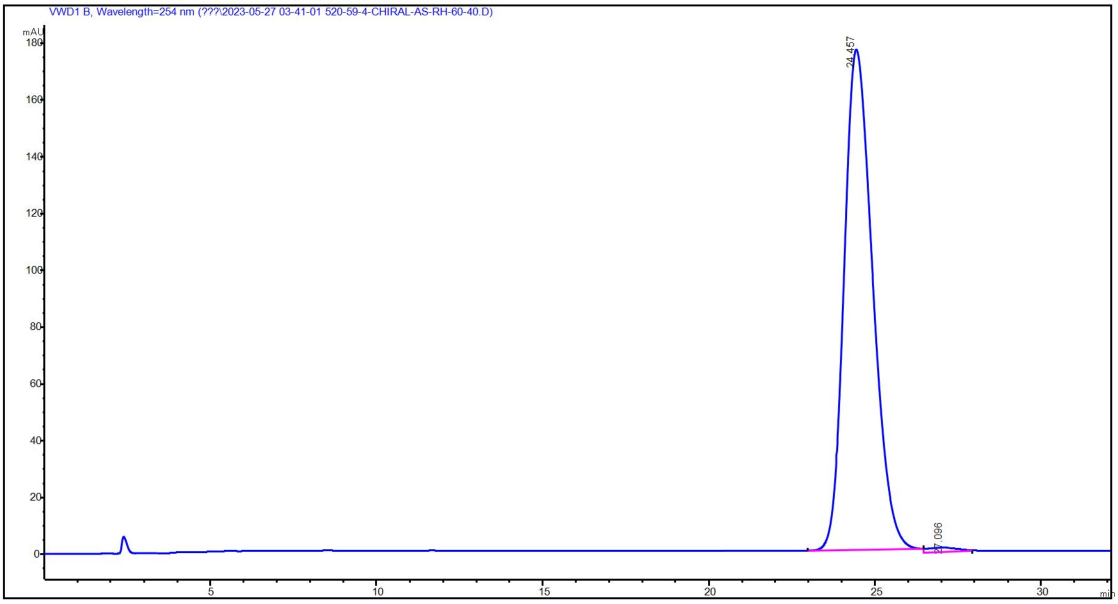

Index	t_R / (min)	Height / (mv)	Area /	Area / (%)
1	24.15	80.8	4365.2	50.0
2	26.67	72.4	4356.1	50.0
Total				100.0

Index	t_R / (min)	Height / (mv)	Area /	Area / (%)
1	24.45	176.3	10501.6	99.0
2	27.09	1.5	102.3	1.0
Total				100.0

(*R*)-naphthalen-2-yl(phenyl)phosphine oxide 12d:

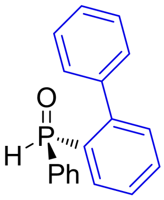

Enantiomeric excess: 95%. 44.5 mg (80% yield), white solid, **m.p.** = 92-96 °C, R_f = 0.25 (PE/EA= 1:2), $[\alpha]_D^{25}$ = -84.4° (c = 1.00 in $CHCl_3$); ^1H NMR (600 MHz, $CDCl_3$) δ 8.28 (s, 0.5H), 7.94 (dd, *J* = 14.0, 8.6 Hz, 1H), 7.59 (t, *J* = 7.6 Hz, 1H), 7.51 (t, *J* = 7.6 Hz, 1H), 7.46 (s, 0.5H), 7.43 – 7.38 (m, 1H), 7.37 – 7.27 (m, 8H), 7.21 (dd, *J* = 7.9, 1.3 Hz, 2H); ^{13}C NMR (151 MHz MHz, $CDCl_3$) δ 139.19 (d, *J* = 5.3 Hz), 132.70 (d, *J* = 10.5 Hz), 132.24 (d, *J* = 2.3 Hz), 131.86 (d, *J* = 2.8 Hz), 131.80 , 131.12 , 130.64 (d, *J* = 9.3 Hz), 130.51 , 130.42 (d, *J* = 11.6 Hz), 129.84 , 129.38 , 128.37 (d, *J* = 13.0 Hz), 128.19 , 127.96 , 127.52 (d, *J* = 12.0 Hz); ^{31}P NMR (121 MHz, $CDCl_3$) δ 18.48; HRMS (ESI-MS) $[M+H]^+$: found 279.0863; calculated for $C_{18}H_{16}OP$: 279.0861.

Chiral HPLC: Chiralpak 98% *ee,* Chiralcel OD-RH column, Water/Acetonitrile = 70/30, flow rate = 1.0 mL/min, λ = 254 nm.

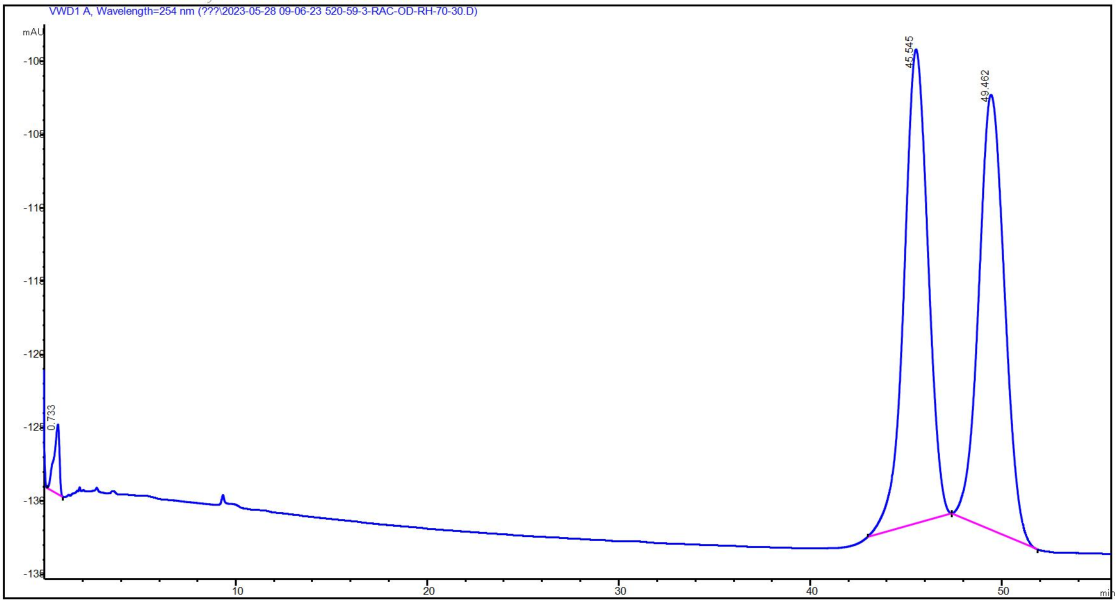

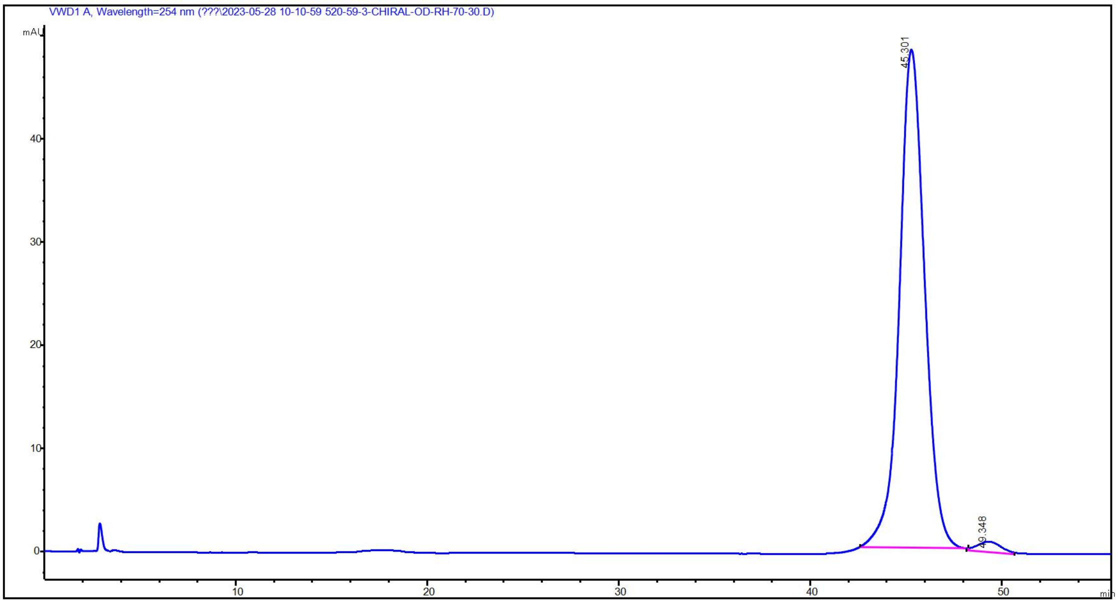

Index	t_R / (min)	Height / (mv)	Area /	Area / (%)
1	45.54	32.3	2811.8	50.3
2	49.46	29.7	2778.1	49.69
Total				100.0

Index	t_R / (min)	Height / (mv)	Area /	Area / (%)
1	45.30	48.2	4413.9	97.5
2	49.32	1.1	98.2	2.5
Total				100.0

X-ray crystal structures

X-ray crystal structure of CAMDOL 1e

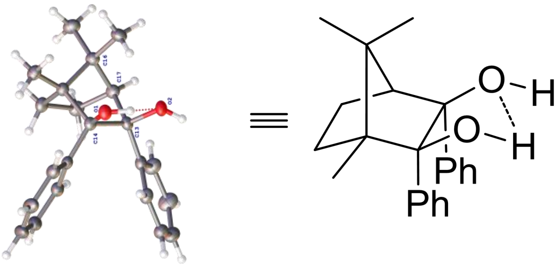

Crystal data for compound **1e**

Identification code	**1e**
Empirical formula	$C_{22}H_{26}O_2$
Formula weight	322.43
Temperature/K	149.99(10)
Crystal system	orthorhombic

Space group	$P2_12_12_1$
a/Å	8.03389(7)
b/Å	10.26993(9)
c/Å	20.93414(19)
α/°	90
β/°	90
γ/°	90
Volume/Å^3	1727.22(3)
Z	4
ρ_{calc}g/cm^3	1.240
μ/mm^{-1}	0.603
F(000)	696.0
Crystal size/mm^3	0.15 × 0.12 × 0.1
Radiation	Cu Kα (λ = 1.54184)
2Θ range for data collection/°	8.448 to 143.902
Index ranges	-9 ≤ h ≤ 9, -12 ≤ k ≤ 12, -25 ≤ l ≤ 21
Reflections collected	9757
Independent reflections	3340 [R_{int} = 0.0199, R_{sigma} = 0.0197]
Data/restraints/parameters	3340/0/225
Goodness-of-fit on F^2	1.053
Final R indexes [I>=2σ (I)]	R_1 = 0.0294, wR_2 = 0.0738
Final R indexes [all data]	R_1 = 0.0298, wR_2 = 0.0742
Largest diff. peak/hole / e Å^{-3}	0.14/-0.19
Flack/Hooft parameter	-0.04(6)/-0.04(5)

X-ray crystal structure of camphor epoxide

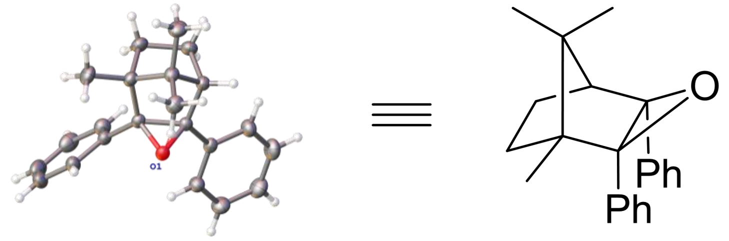

Deposition No. CCDC-2233676

Crystal data for compound camphor epoxide

Identification code	Camphor epoxide
Empirical formula	$C_{22}H_{24}O$
Formula weight	304.41

Temperature/K	295.02(10)
Crystal system	monoclinic
Space group	I2
a/Å	10.2357(2)
b/Å	9.9381(2)
c/Å	16.7995(4)
α /°	90
β /°	95.2966(19)
γ /°	90
Volume/Å^3	1701.61(6)
Z	4
ρ_{calc}g/cm^3	1.188
μ /mm^{-1}	0.540
F(000)	656.0
Crystal size/mm^3	0.15 × 0.13 × 0.1
Radiation	Cu Kα (λ = 1.54184)
2Θ range for data collection/°	9.736 to 147.95
Index ranges	-11 ≤ h ≤ 12, -12 ≤ k ≤ 12, -18 ≤ l ≤ 20
Reflections collected	9057
Independent reflections	3334 [R_{int} = 0.0342, R_{sigma} = 0.0247]
Data/restraints/parameters	3334/1/211
Goodness-of-fit on F^2	1.106
Final R indexes [I>=2σ (I)]	R_1 = 0.0456, wR_2 = 0.1188
Final R indexes [all data]	R_1 = 0.0470, wR_2 = 0.1199
Largest diff. peak/hole / e Å^{-3}	0.19/-0.35
Flack parameter	0.06(11)

X-ray crystal structure of product 5bo

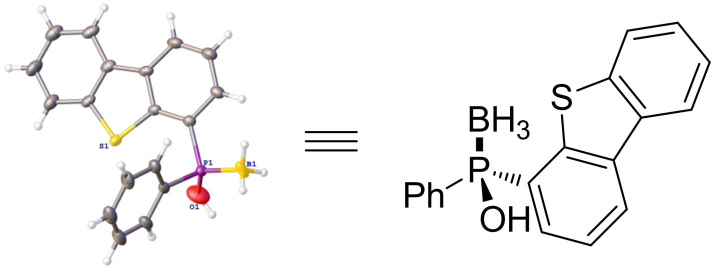

Crystal data for compound **5bo**

Identification code	**5bo**
Empirical formula	$C_{18}H_{16}BOPS$
Formula weight	322.15
Temperature/K	170.01(10)
Crystal system	triclinic
Space group	P-1
a/Å	9.73780(10)
b/Å	11.88200(10)
c/Å	14.5772(2)
α /°	89.4940(10)
β /°	87.5190(10)
γ /°	82.2680(10)
Volume/Å^3	1669.74(3)
Z	4
ρ_{calc}g/cm^3	1.281
μ /mm^{-1}	2.593
F(000)	672.0
Crystal size/mm^3	0.13 × 0.12 × 0.1
Radiation	Cu Kα (λ = 1.54184)
2Θ range for data collection/°	6.068 to 143.226
Index ranges	-11 ≤ h ≤ 11, -11 ≤ k ≤ 14, -17 ≤ l ≤ 17
Reflections collected	16735
Independent reflections	6310 [R_{int} = 0.0186, R_{sigma} = 0.0195]
Data/restraints/parameters	6310/378/402
Goodness-of-fit on F^2	1.256
Final R indexes [I>=2σ (I)]	R_1 = 0.1335, wR_2 = 0.2898
Final R indexes [all data]	R_1 = 0.1338, wR_2 = 0.2899
Largest diff. peak/hole / e Å^{-3}	1.58/-1.31

Supplementary References

1. Knouse, K. W.; deGruyter, J. N.; Schmidt, M. A.; Zheng, B.; Vantourout, J. C.; Kingston, C.; Mercer, S. E.; McDonald, I. M.; Olson, R. E.; Zhu, Y.; Hang, C.; Zhu, J.; Yuan, C.; Wang, Q.; Park, P.; Eastgate, M. D.; Baran, P. S., Unlocking P(V): Reagents for chiral phosphorothioate synthesis. *Science* **2018,** *361*, 1234.

2. Woźniak, L. A.; Wieczorek, M.; Pyzowski, J.; Majzner, W.; Stec, W. J. Stereochemistry of the DBU/LiCl-Assisted Nucleophilic Substitution at Phosphorus in Nucleoside-3‘-O-(Se-methyl

Methanephosphonoselenolate). *J. Org. Chem.* **1998,** *63*,5395–5402.

4. Krasovskiy, A.; Knochel, P., Convenient Titration Method for Organometallic Zinc, Magnesium, and Lanthanide- Reagents. *Synthesis* **2006,** *2006*, 890–891.

5. Han, Z. S.; Goyal, N.; Herbage, M. A.; Sieber, J. D.; Qu, B.; Xu, Y.; Li, Z.; Reeves, J.T.; Desrosiers, J.-N.; Ma, S.; Grinberg, N.; Lee, H.; Mangunuru, H. P. R.; Zhang, Y.; Krishnamurthy, D.; Lu, B. Z.; Song, J. J.; Wang, G.; Senanayake, C. H., Efficient Asymmetric Synthesis of P-Chiral Phosphine Oxides via Properly Designed andActivated Benzoxazaphosphinine-2-oxide Agents. *J. Am. Chem. Soc.* **2013,** *135*, 2474–2477.

6. Rajendran, K. V.; Gilheany, D. G., Simple unprecedented conversion of phosphine oxides and sulfides to phosphine boranes using sodium borohydride. *Chem. Comm.* **2012,** *48*, 817–819.

7. Paul, S.; Roy, S.; Monfregola, L.; Shang, S.; Shoemaker, R.; Caruthers, M. H., Oxidative Substitution of Boranephosphonate Diesters as a Route toPost synthetically Modified DNA. *J. Am. Chem. Soc.* **2015,** *137*, 3253–3264.

8. Huang, H.-S.; Kong, R.; Zheng, X.-A.; Chen, W.-J.; Han, S.-B.; Zeng, D.-Y.; Gong, S.-S.; Sun, Q., A Practical Method for Regioselective 5′-O-tert-ButyldimethylsilylDeprotection of Persilylated Nucleosides by Methanolic Phosphomolybdic Acid. *Synlett* **2018,** *29*, 2437–2443.
