# Supplementary material for: Universal and divergent P-stereogenic building with camphor-derived 2,3-diols
Source: Commun Chem. 2023 Jun 27;6:133. doi: 10.1038/s42004-023-00935-0 (PMC10300088; doi:10.1038/s42004-023-00935-0)
Supplement: Supplementary file 3 — Description of Additional Supplementary [file 42004_2023_935_MOESM3_ESM.pdf]

Description of Additional Supplementary Files

File name: Supplementary Data 1
Description: NMR spectra.

File name: Supplementary Data 2
Description: Cif file of **1e**

File name: Supplementary Data 3
Description: Cif file of camphor epoxide

File name: Supplementary Data 4
Description: Cif file of **5bo**
