# Supplementary material for: Universal and divergent P-stereogenic building with camphor-derived 2,3-diols
Source: Commun Chem. 2023 Jun 27;6:133. doi: 10.1038/s42004-023-00935-0 (PMC10300088; doi:10.1038/s42004-023-00935-0)

**^1^H, ^13^C, and ^31^P NMR Spectra**

**Compound 1a ^1^H NMR**

**Compound 1a ^13^C NMR**

**Compound 1b ^1^H NMR**

**Compound 1b ^13^C NMR**


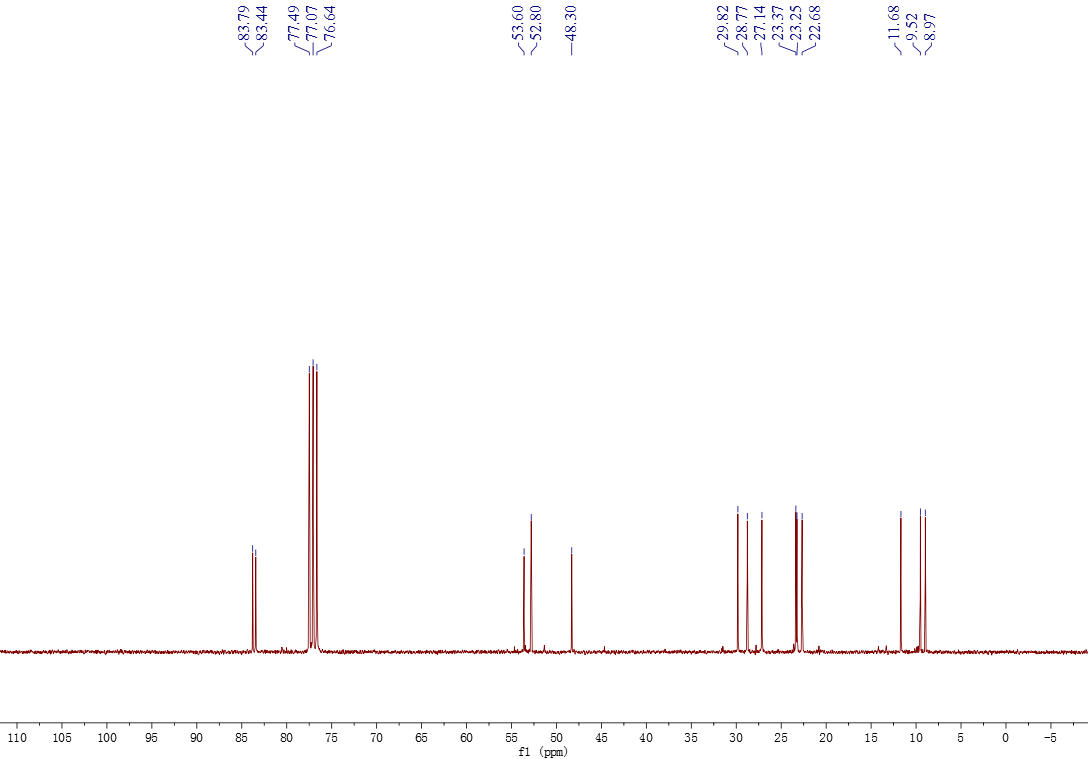


**Compound 1c ^1^H NMR**

**Compound 1c ^13^C NMR**

**Compound 1d ^1^H NMR**

**Compound 1d ^13^C NMR**

**Compound 1e ^1^H NMR**

**Compound 1e ^13^C NMR**

**Compound 3b ^1^H NMR**

**Compound 3b ^13^C NMR**

**Compound 3b ^31^P NMR**

**Compound 3c ^1^H NMR**

**Compound 3c ^13^C NMR**

**Compound 3c ^31^P NMR**

**Compound camphor epoxide ^1^H NMR**

**Compound camphor epoxide ^13^C NMR**

**Compound 4ce ^1^H NMR**

**Compound 4ce ^13^C NMR**

**Compound 4ce ^31^P NMR**

**Compound 4be ^1^H NMR**

**Compound 4be ^13^C NMR**

**Compound 4be ^31^P NMR**

**Compound 5aa ^1^H NMR**

**Compound 5aa ^13^C NMR**

**Compound 5aa ^31^P NMR**

**Compound 5ab ^1^H NMR**

**Compound 5ab ^13^C NMR**

**Compound 5ab ^31^P NMR**

**Compound 5ac ^1^H NMR**

**Compound 5ac ^13^C NMR**

**Compound 5ac ^31^P NMR**

**Compound 5ad ^1^H NMR**

**Compound 5ad ^13^C NMR**

**Compound 5ad ^31^P NMR**

**Compound 5ae ^1^H NMR**

**Compound 5ae ^13^C NMR**

**Compound 5ae ^31^P NMR**

**Compound 5af ^1^H NMR**

**Compound 5af ^13^C NMR**

**Compound 5af ^31^P NMR**

**Compound 5ag ^1^H NMR**

**Compound 5ag ^13^C NMR**

**Compound 5ag ^31^P NMR**

**Compound 5ah ^1^H NMR**

**Compound 5ah ^13^C NMR**

**Compound 5ah ^31^P NMR**

**Compound 5ai ^1^H NMR**


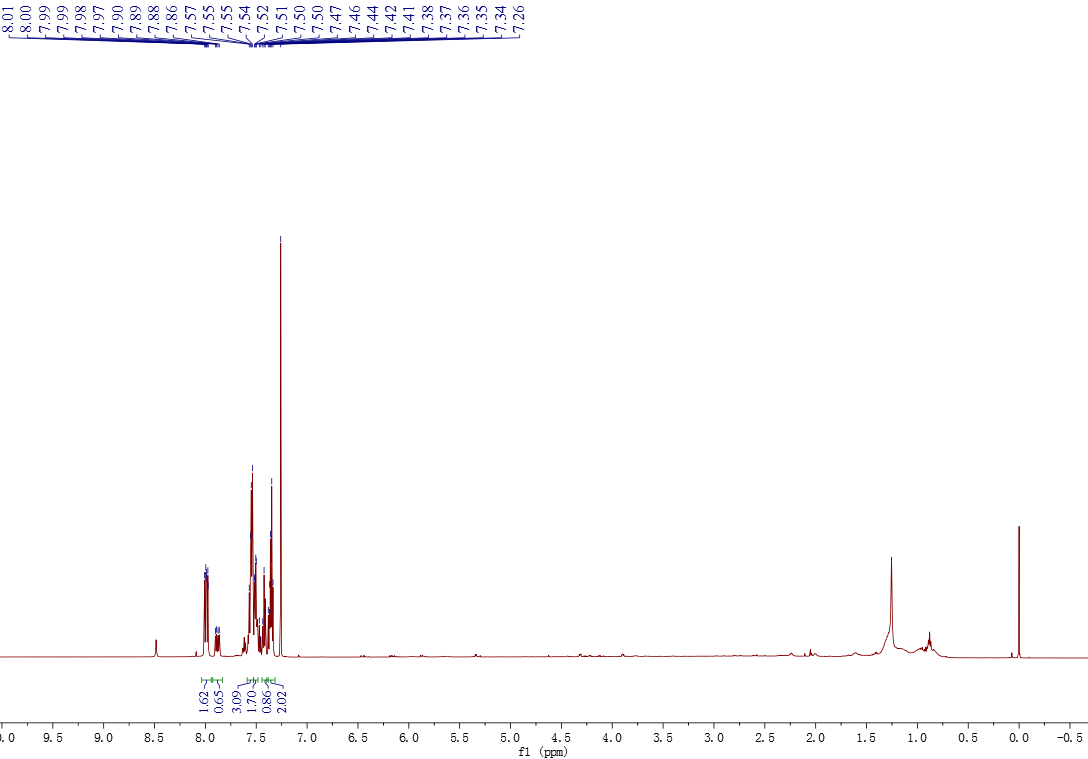

**Compound 5ai ^13^C NMR**


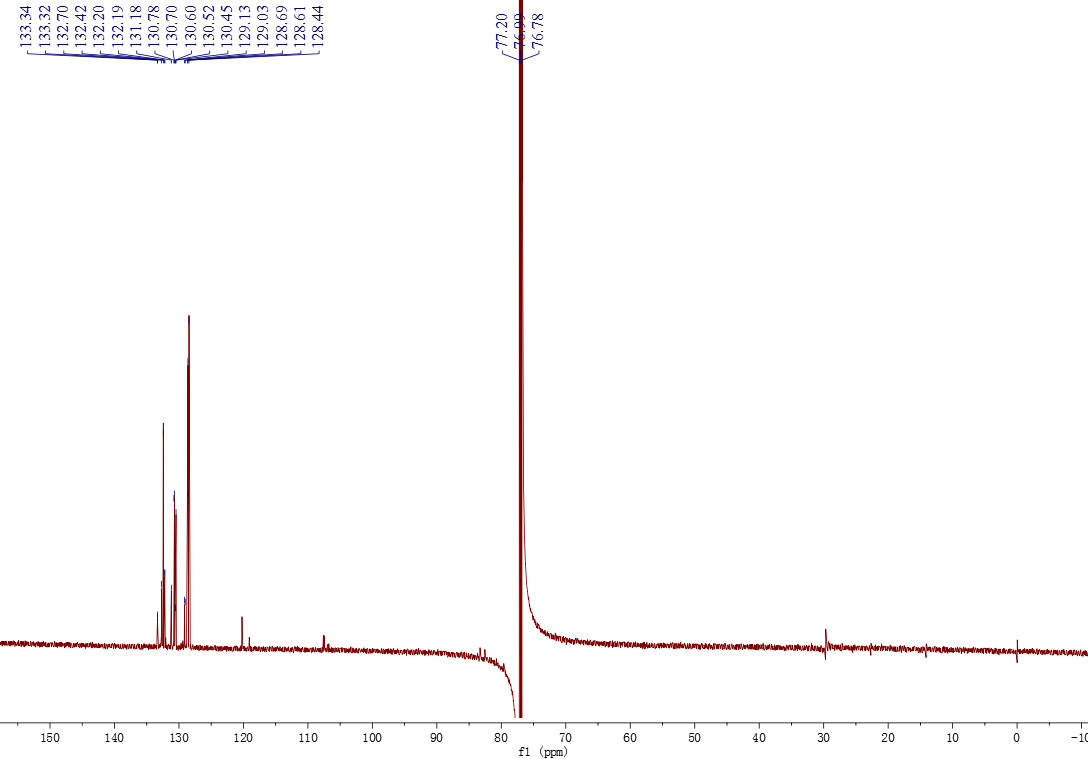

**Compound 5ai ^31^P NMR**


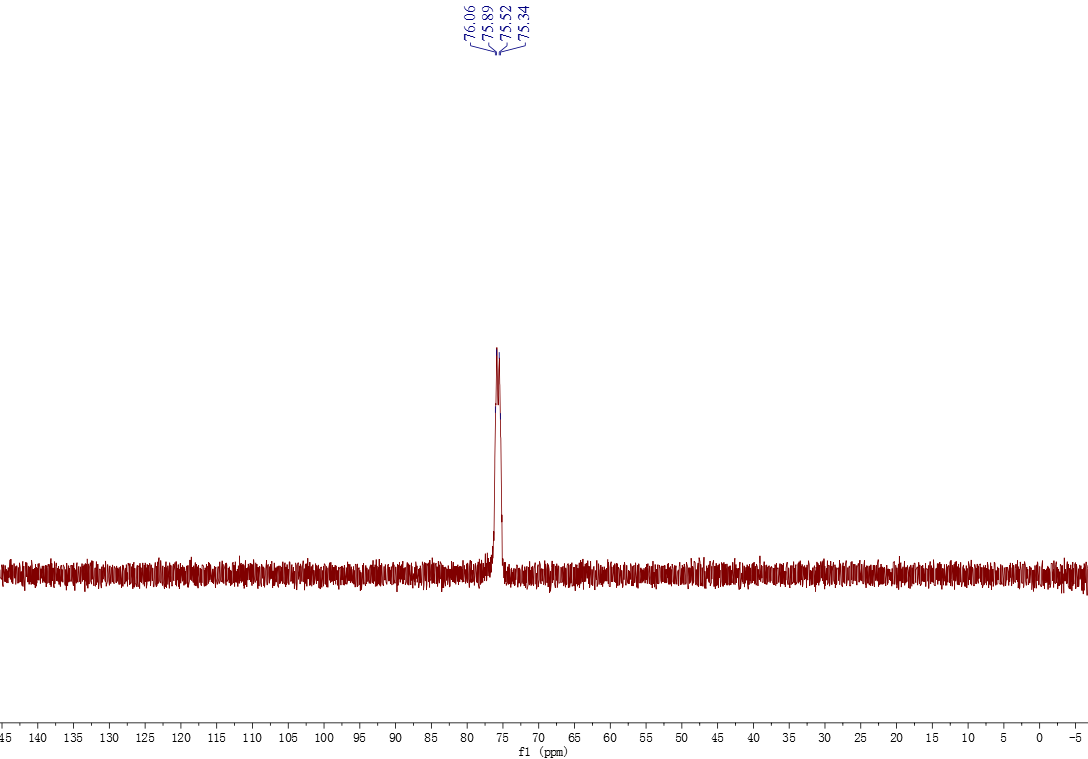

**Compound 5aj ^1^H NMR**

**Compound 5aj ^13^C NMR**

**Compound 5aj ^31^P NMR**

**Compound 5ak ^1^H NMR**

**Compound 5ak ^13^C NMR**

**Compound 5ak ^31^P NMR**

**Compound 5ba ^1^H NMR**

**Compound 5ba ^13^C NMR**

**Compound 5ba ^31^P NMR**

**Compound 5bb ^1^H NMR**

**Compound 5bb ^13^C NMR**

**Compound 5bb ^31^P NMR**

**Compound 5bc ^1^H NMR**

**Compound 5bc ^13^C NMR**

**Compound 5bc ^31^P NMR**

**Compound 5bd ^1^H NMR**

**Compound 5bd ^13^C NMR**

**Compound 5bd ^31^P NMR**

**Compound 5be ^1^H NMR**

**Compound 5be ^13^C NMR**

**Compound 5be ^31^P NMR**

**Compound 5bf ^1^H NMR**

**Compound 5bf ^13^C NMR**

**Compound 5bf ^31^P NMR**

**Compound 5bg ^1^H NMR**

**Compound 5bg ^13^C NMR**

**Compound 5bg ^31^P NMR**

**Compound 5bh ^1^H NMR**

**Compound 5bh ^13^C NMR**

**Compound 5bh ^31^P NMR**

**Compound 5bi ^1^H NMR**

**Compound 5bi ^13^C NMR**

**Compound 5bi ^31^P NMR**

**Compound 5bj ^1^H NMR**

**Compound 5bj ^13^C NMR**

**Compound 5bj ^31^P NMR**

**Compound 5bk ^1^H NMR**

**Compound 5bk ^13^C NMR**

**Compound 5bk ^31^P NMR**

**Compound 5bl ^1^H NMR**

**Compound 5bl ^13^C NMR**

**Compound 5bl ^31^P NMR**

**Compound 5bm ^1^H NMR**

**Compound 5bm ^13^C NMR**

**Compound 5bm ^31^P NMR**

**Compound 5bn ^1^H NMR**

**Compound 5bn ^13^C NMR**

**Compound 5bn ^31^P NMR**

**Compound 5bo ^1^H NMR**

**Compound 5bo ^13^C NMR**

**Compound 5bo ^31^P NMR**

**Compound 5bp ^1^H NMR**

**Compound 5bp ^13^C NMR**

**Compound 5bp ^31^P NMR**

**Compound 5bq ^1^H NMR**

**Compound 5bq ^13^C NMR**

**Compound 5bq ^31^P NMR**

**Compound 5br ^1^H NMR**

**Compound 5br ^13^C NMR**

**Compound 5br ^31^P NMR**

.

**Compound 5ca ^1^H NMR**

**Compound 5ca ^13^C NMR**

**Compound 5ca ^31^P NMR**

**Compound 5cb ^1^H NMR**

**Compound 5cb ^13^C NMR**

**Compound 5cb ^31^P NMR**

**Compound 5cc ^1^H NMR**

**Compound 5cc ^13^C NMR**

**Compound 5cc ^31^P NMR**

**Compound 5cc ^19^F NMR**

**Compound PAMP ^1^H NMR**

**Compound PAMP ^13^C NMR**

**Compound PAMP ^31^P NMR**

**Compound 6a ^1^H NMR**

**Compound 6a ^13^C NMR**

**Compound 6a ^31^P NMR**

**Compound 6b ^1^H NMR**

**Compound 6b ^13^C NMR**

**Compound 6b ^31^P NMR**

**Compound 7a ^1^H NMR**

**Compound 7a ^13^C NMR**

**Compound 7a ^31^P NMR**

**Compound 7b ^1^H NMR**

**Compound 7b^31^P NMR**

**Compound 7b ^13^C NMR**

**Compound 7b ^31^P NMR**

**Compound 8a ^1^H NMR**

**Compound 8a ^13^C NMR**

**Compound 8a ^31^P NMR**

**Compound 8a ^1^H NMR**

**Compound 8a ^13^C NMR**

**Compound 8a ^31^P NMR**

**Compound 9b ^1^H NMR**

**Compound 9b ^13^C NMR**

**Compound 9b ^31^P NMR**

**Compound 10b ^1^H NMR**

**Compound 10b ^13^C NMR**

**Compound 10b ^31^P NMR**

**Compound 11b ^1^H NMR**

**Compound 11b ^13^C NMR**

**Compound 11b ^31^P NMR**

**Compound 12a ^1^H NMR**


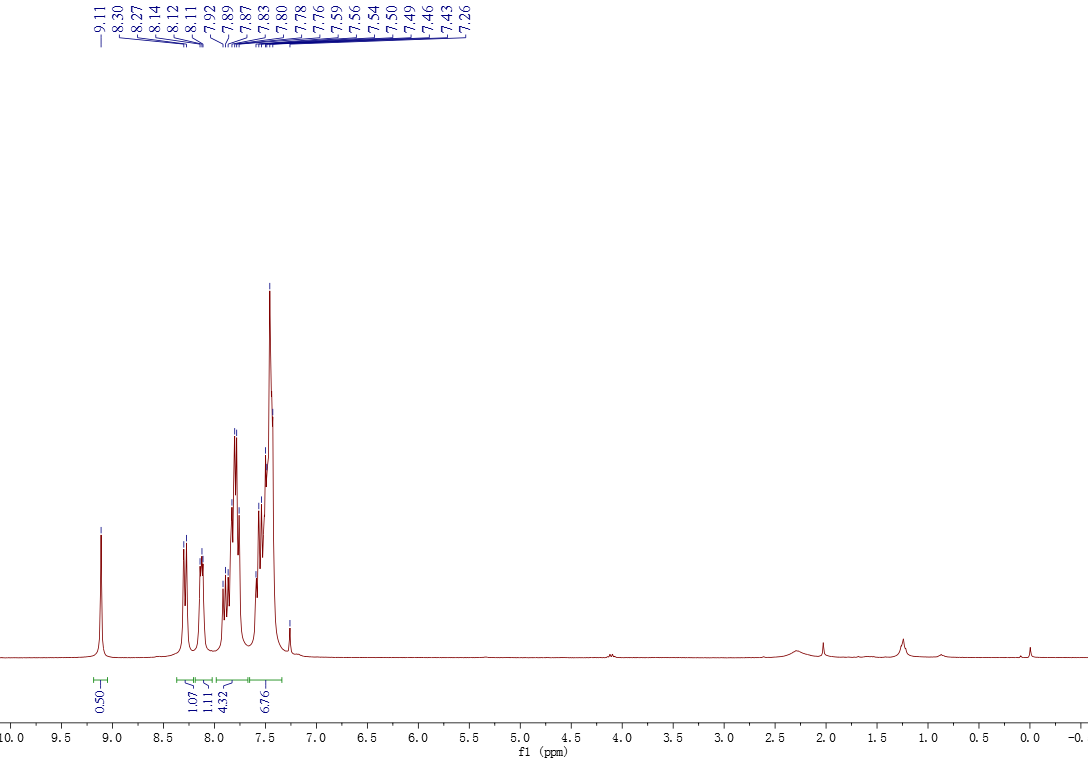


**Compound 12a ^13^C NMR**


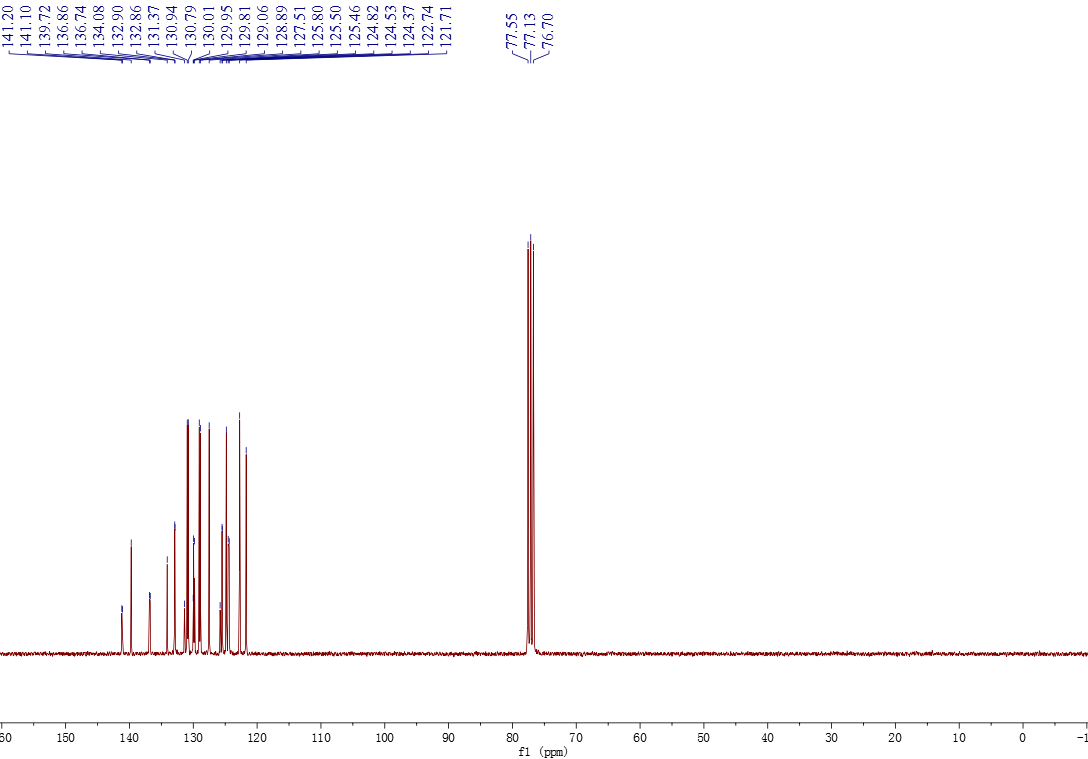


**Compound 12a ^31^P NMR**


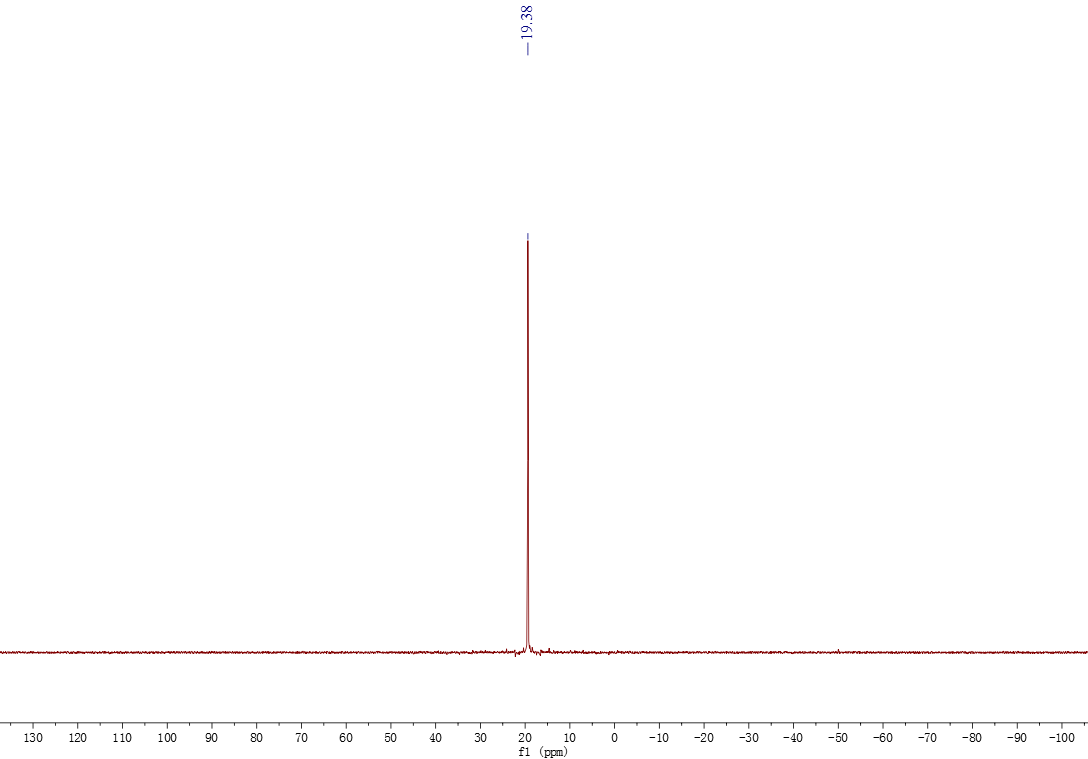


**Compound 12b ^1^H NMR**


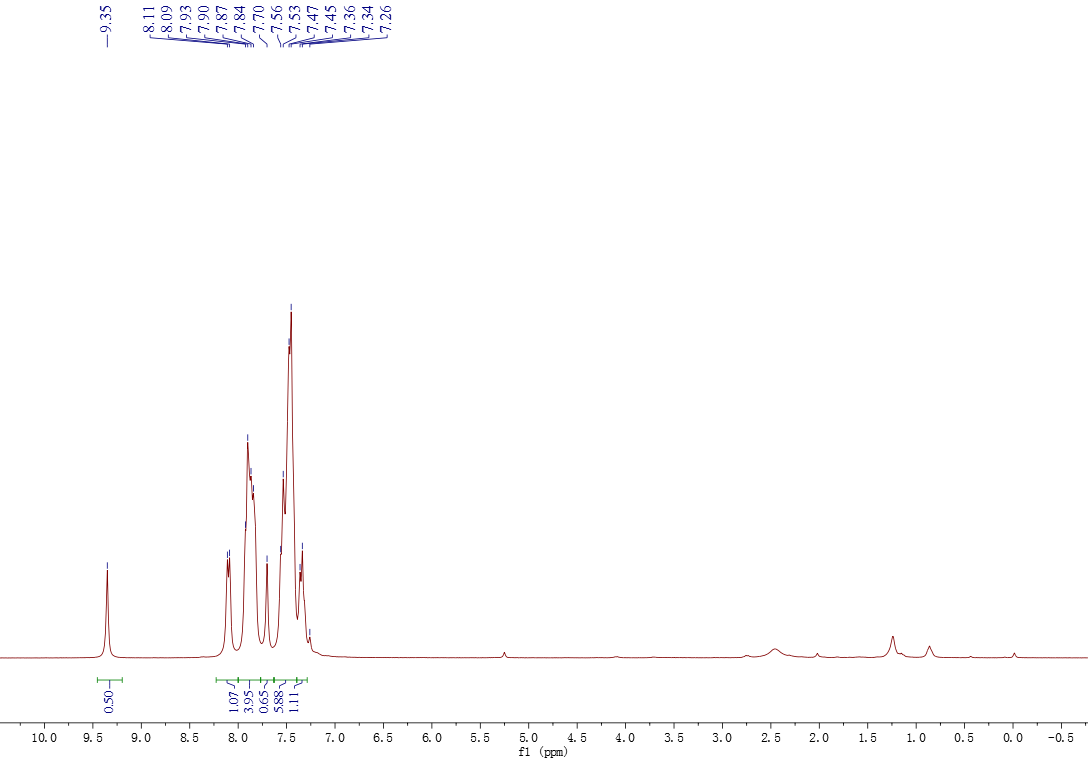


**Compound 12b ^13^C NMR**


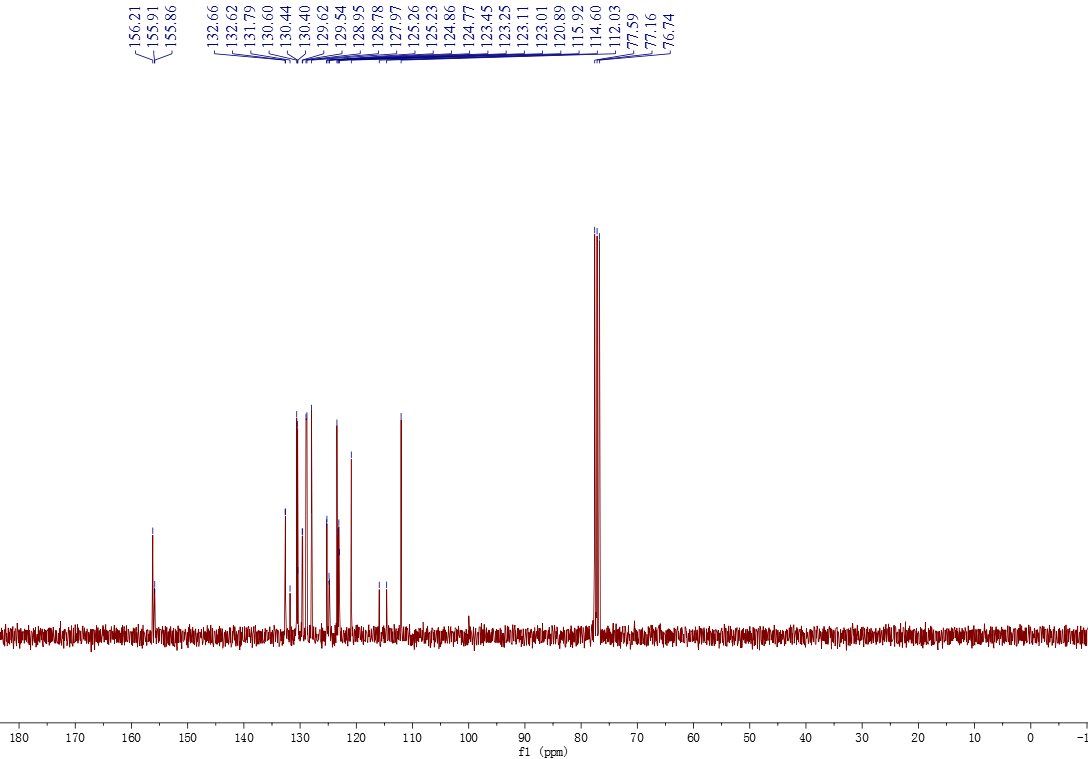


**Compound 12b ^31^P NMR**


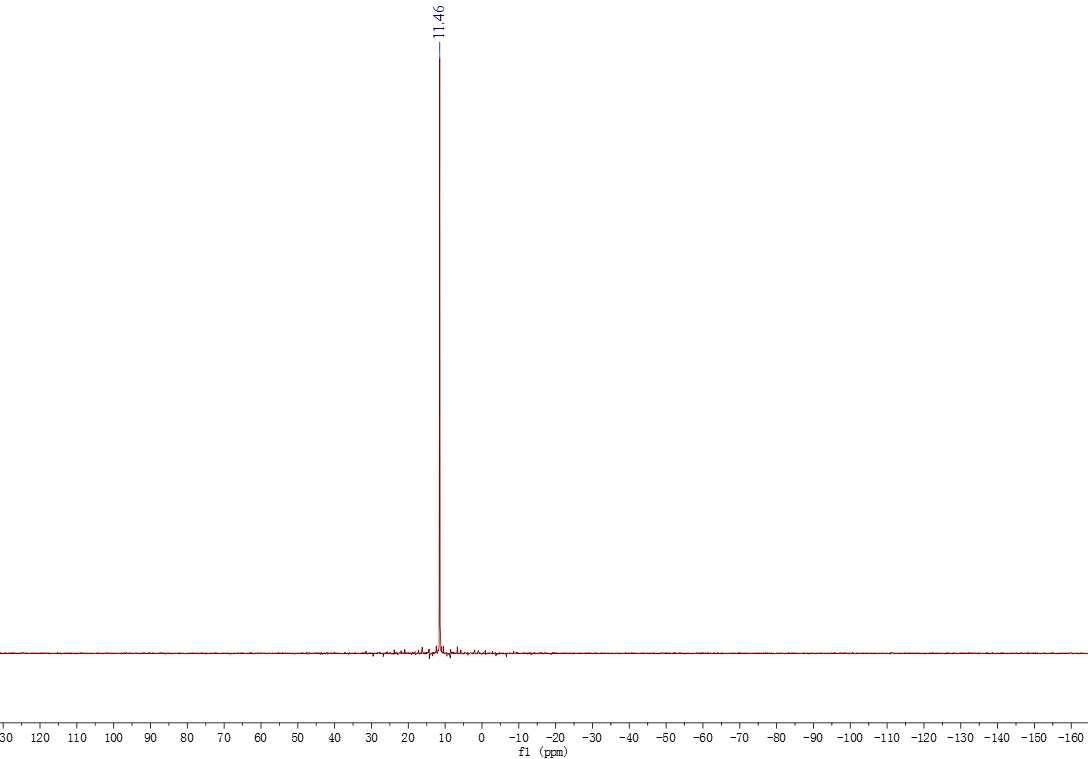


**Compound 12c ^1^H NMR**


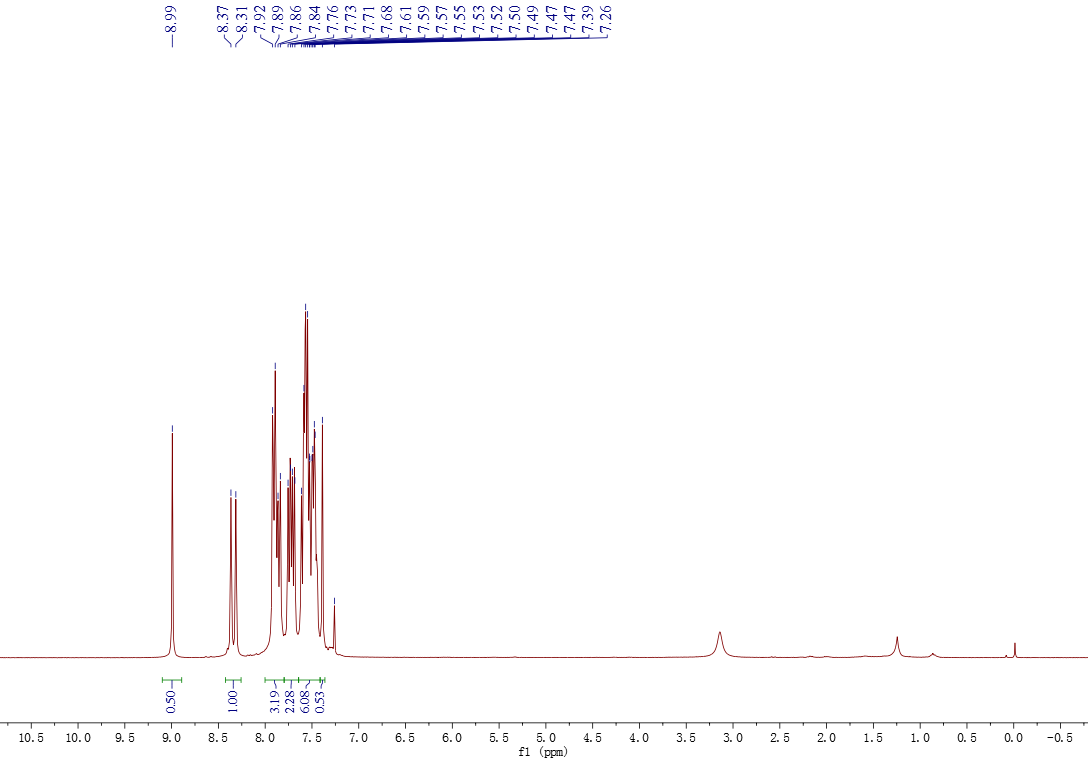


**Compound 12c ^13^C NMR**


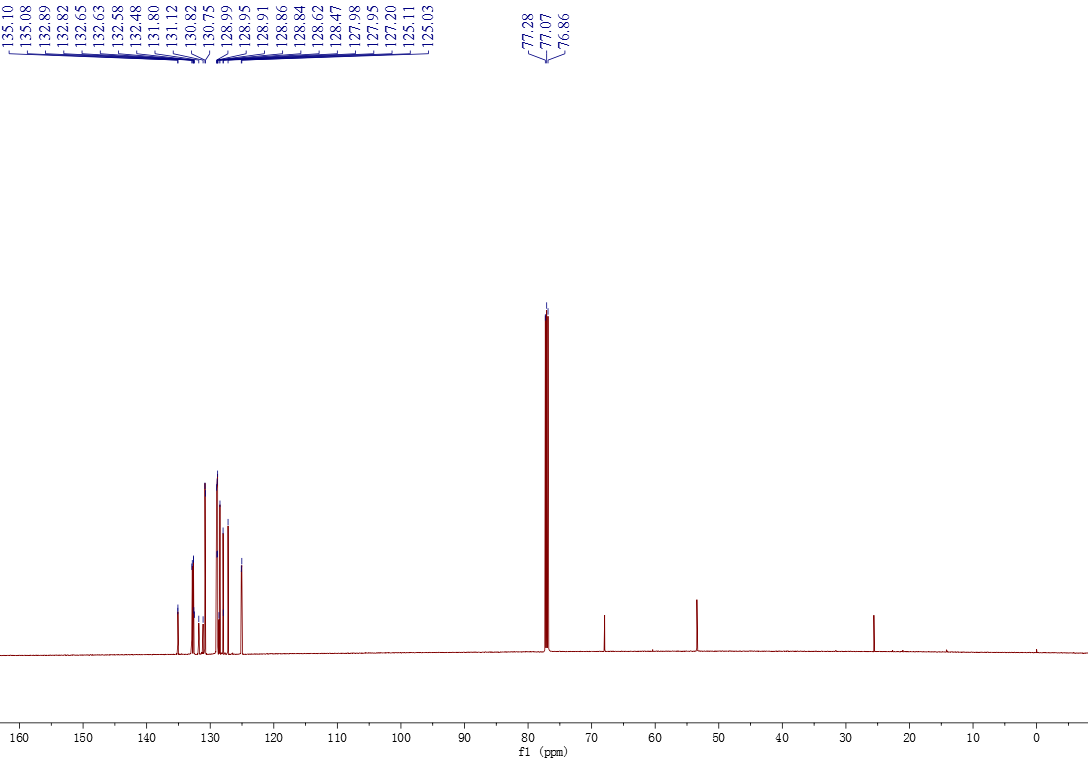


**Compound 12c ^31^P NMR**


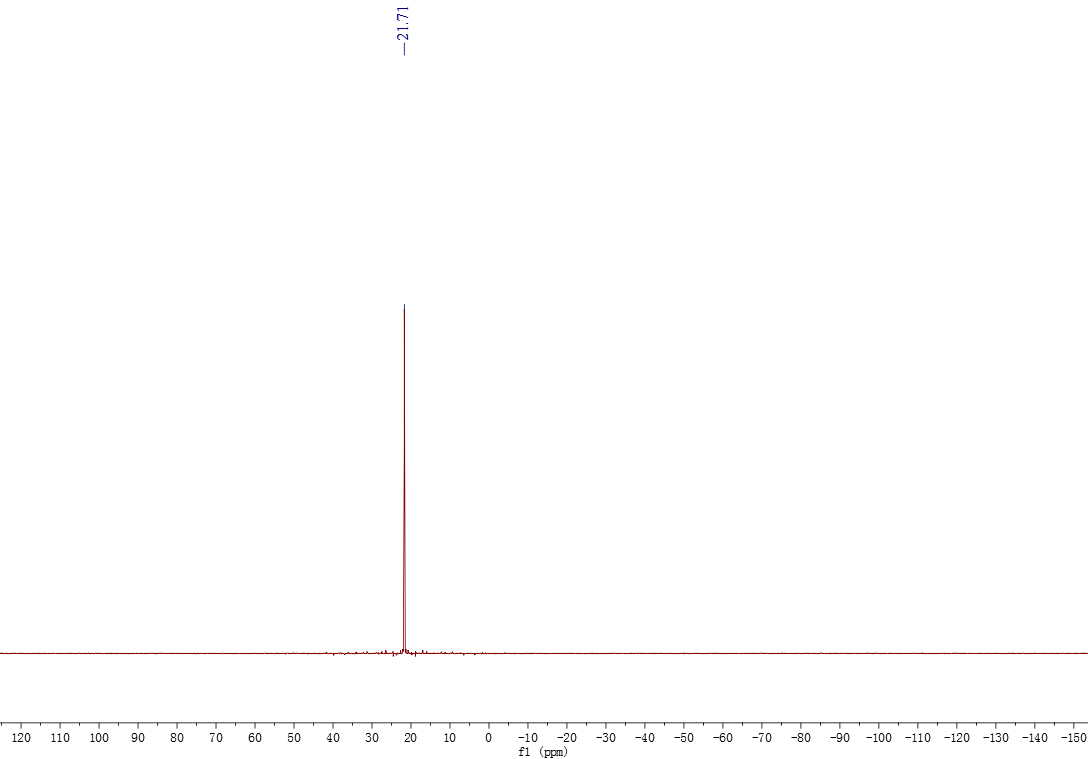


**Compound 12d ^1^H NMR**


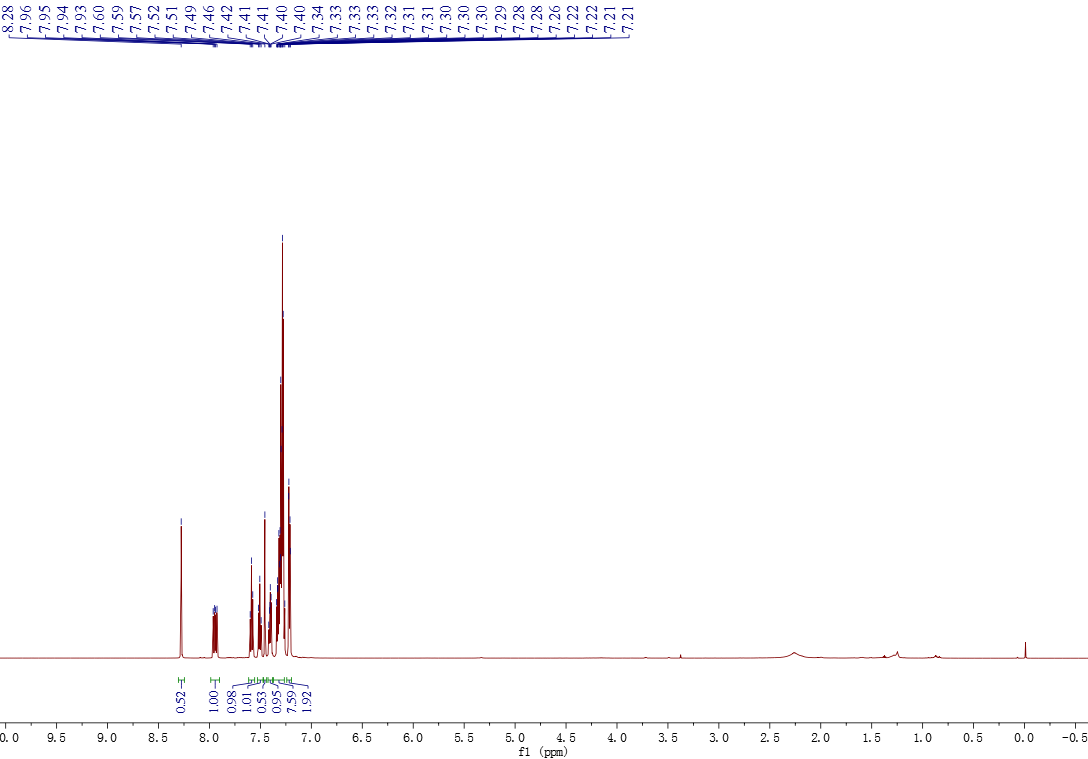


**Compound 12d ^13^C NMR**


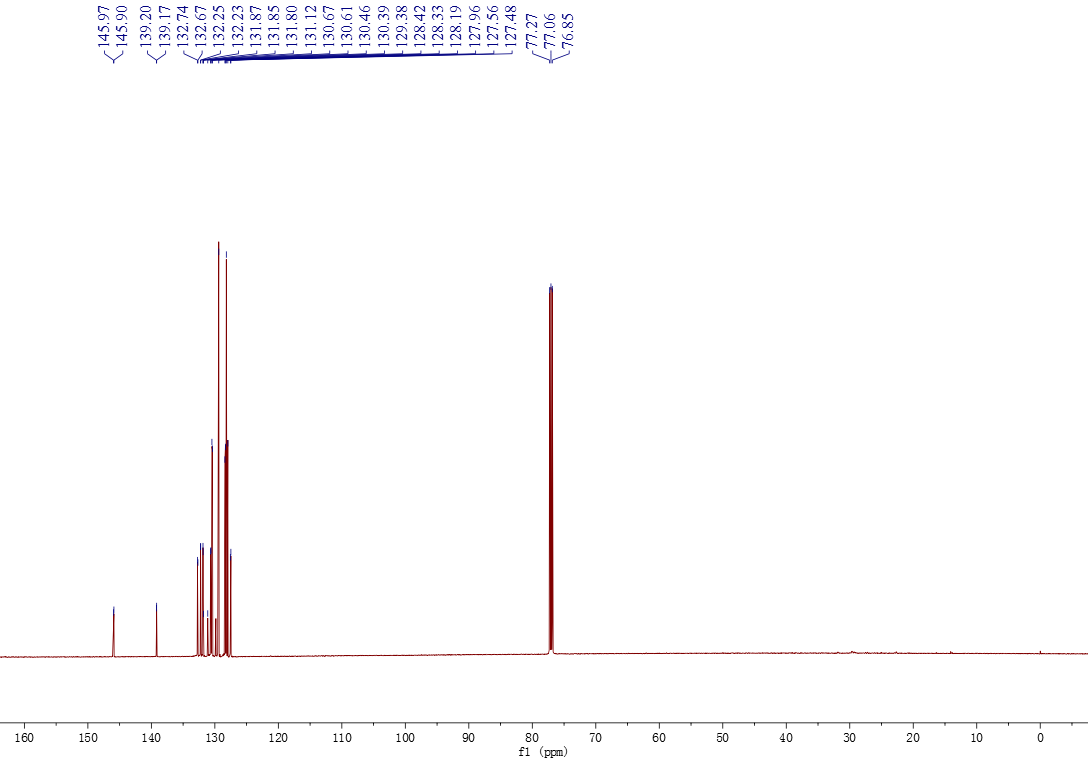


**Compound 12d ^31^P NMR**


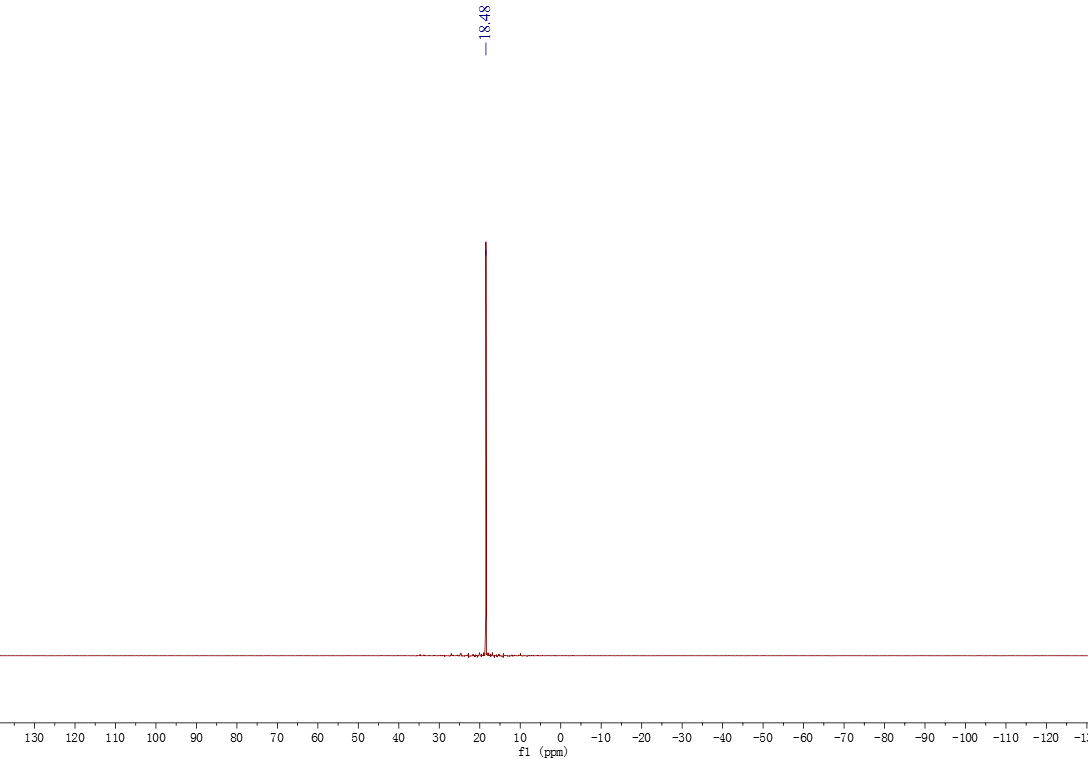

Supplement: Supplementary file 4 — Supplementary Data 1–4 [file 42004_2023_935_MOESM4_ESM.zip › Supplementary Data 1-4/Supplementary Data 1- COMMSCHEM-23-0218B.docx]
